# Novel indole-based scaffolds: Design, synthesis, molecular modeling, and anti-proliferative evaluation

**DOI:** 10.1186/s13065-026-01776-3

**Published:** 2026-04-09

**Authors:** Reham A. Mohamed-Ezzat, Aisha A. K. Al-Ashmawy, Aladdin M. Srour

**Affiliations:** 1 Chemistry of Natural and Microbial Products Department, Pharmaceutical and Drug Industries Research Institute, National Research Centre, Cairo, Egypt; 2 Department of Therapeutic Chemistry, Pharmaceutical and Drug Industries Research Institute, National Research Centre, Dokki, Cairo, 12622 Egypt

**Keywords:** Synthesis, Indole, Sulfonate, Antitumor, NCI 60-cell, CDK2.

## Abstract

Two novel series of indole-based scaffolds have been designed, synthesized, and screened for their anti-proliferative activities on the NCI-60 cell line panel. The novel structures of the indole-sulfonate and indole-aspirin mimic conjugates were designed as cyclin-dependent kinase 2 (CDK-2) inhibitors. The design approach depends on molecular hybridization between the indole scaffold and alkanesulfonates or with aspirin mimic analogs. The synthesized compounds were screened for their cytotoxic activity at a concentration of 10 *µ*M. Compound **8a** exhibited the most potent anticancer activity among the tested series and was further selected for the five-dose stage. At sub-micromolar doses, compound **8a** showed anti-cancer potency against a panel of 60 tumor cell lines, with log GI_50_ values in the range from − 4.50 *µ*M to -6.45 µM. With log GI_50_ values of -6.45, -6.42, -6.31, -5.96, and − 5.87 *µ*M, respectively, the most sensitive cell lines were leukemia (SR), melanoma (MDA-MB-435), CNS cancer (SNB-75), ovarian cancer (OVCAR-3), and non-small cell lung cancer (NCI-H522). All the compounds were tested in an *insilico* study, encompassing drug likeness and absorption, distribution, metabolism, and excretion (ADME) prediction. A molecular modelling simulation study of the promising compound **8a** in CDK-2 revealed the potential in vitro CDK-2 inhibitory assay result. The results demonstrate that, upon optimization, these novel indole-sulfonates could potentially act as new anticancer drugs.

**Supplementary Information:**

The online version contains supplementary material available at 10.1186/s13065-026-01776-3.

## Introduction

The cyclin-dependent kinases (CDKs) are protein kinases that regulate transcription in response to various intracellular and extracellular stimuli and are essential for controlling cell division [1]. They serve as key regulatory elements necessary for the progression of the cell cycle. Cell cycle CDK levels are typically stable, while cyclins-proteins whose levels fluctuate during each cell cycle, control their activity. Beyond their traditional roles, it has been demonstrated that other members of the CDK family are important for diverse processes such as regulating gene transcription, metabolism, and neuronal function [2]. The evolutionary expansion of the CDK family in mammals resulted in the division of CDKs into five transcriptional subfamilies (Cdk7, Cdk8, Cdk9, Cdk11, and Cdk20) and three cell-cycle-related subfamilies (Cdk1, Cdk4, and Cdk5) [1]. Structurally, the serine/threonine-specific catalytic core of cyclin-dependent kinases (Cdks) is associated with regulatory subunits called cyclins, which determine substrate specificity and kinase activity [3], as well as provide domains essential for enzymatic function [1]. It is well established that cyclins and their catalytic partners, cyclin-dependent kinases (CDKs), are vital components that drive the cell cycle forward. In addition, mammalian cyclins and CDKs play important roles in transcription, DNA damage repair, regulation of cell death, differentiation, immune responses, and metabolism [4]. A highly conserved family of protein kinases that regulate the eukaryotic cell cycle includes cyclin-dependent kinase 2 (CDK2) [5]. Cells enter the S- and M-phases of the cell cycle under the influence of CDK2. Although CDK2 activity is often considered non-essential for normal development, it is closely associated with tumor growth in various cancers. Despite ongoing debates about CDK2’s role in cancer development, recent research suggests that targeted inhibition of CDK2 could be therapeutically beneficial for certain malignancies, making it a promising target for anticancer drug development. Several small-molecule CDK2 inhibitors have progressed to clinical trials; however, a truly selective CDK2 inhibitor has yet to be identified [6]. Due in significant part to difficulties in obtaining proper selectivity and excellent therapeutic indices, there is currently no approved selective CDK2 inhibitor [7]. None have been authorized for the clinical use, while several are in preclinical or clinical development [8, 9]. Improved selectivity profiles and promising early efficacy and tolerability findings are demonstrated by next-generation selective inhibitors (e.g., INX-315, BLU-222, PF-07104091, INCB123667), especially in malignancies with CCNE1 amplification or CDK4/6 inhibitor resistance [10–14]. These compounds are moving through early clinical trials, and some biomarker techniques (e.g., CCNE1, p16/Biomarkers, Rb status, ) are being assessed to optimize the patient selection and the response prediction [15]. Therefore, targeting CDK2 remains an attractive strategy in anticancer drug discovery [16].

In this article, novel indoles were designed, synthesized, and biologically evaluated as potential anti-cancer agents. The in vitro anti-proliferative activities of most of the novel compounds were evaluated on the NCI-60 cell line panel. The design of the compound depended on important pharmacophores. On one front, a common scaffold in drug design and development studies, indole is a favored moiety with a variety of bioactivities [17]. Indole and its derivatives are important in medical chemistry. Due to its physiological action, which includes anti-inflammatory, antiviral, antimalarial, anticancer, antitubercular, antimicrobial, antileishmanial, anti-cholinesterase, and enzyme inhibitory properties [18]. Indole-based compounds as a substantial class of chemicals emphasize their tasks in the treatment of cancer, the control of infectious diseases, anti-inflammatory medications, treatments for metabolic disorders, and the control of neurodegenerative diseases. Derivatives of indole have demonstrated notable effectiveness in addressing a variety of biological processes. Notably, these compounds offer prospective treatment possibilities for chronic disorders, including diabetes and hypertension, and have shown promise in fighting drug-resistant cancer cells and pathogens [19].

**Fig. 1:**
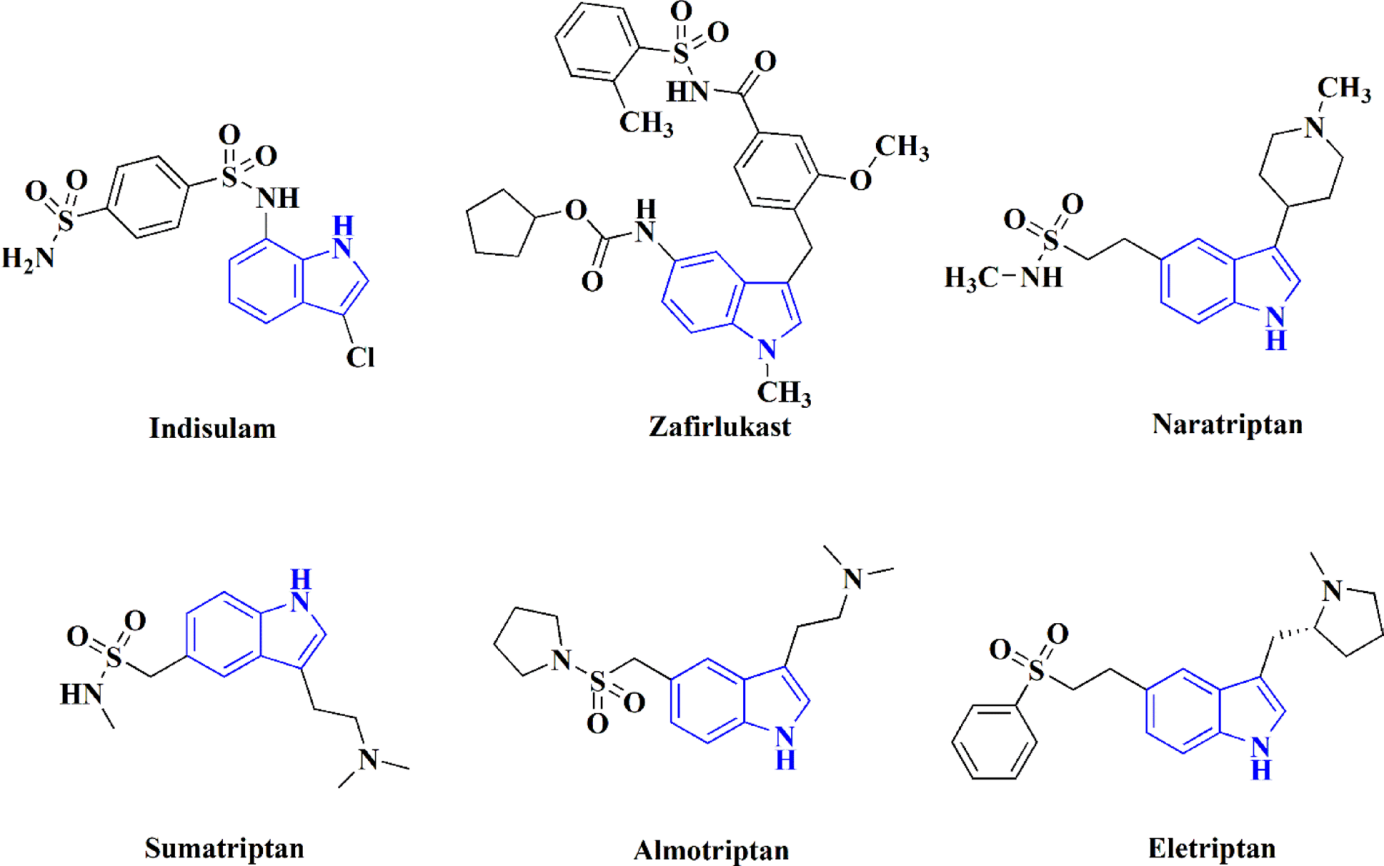
Representative examples of some indole-sulfonyl-based drugs

**Fig. 2:**
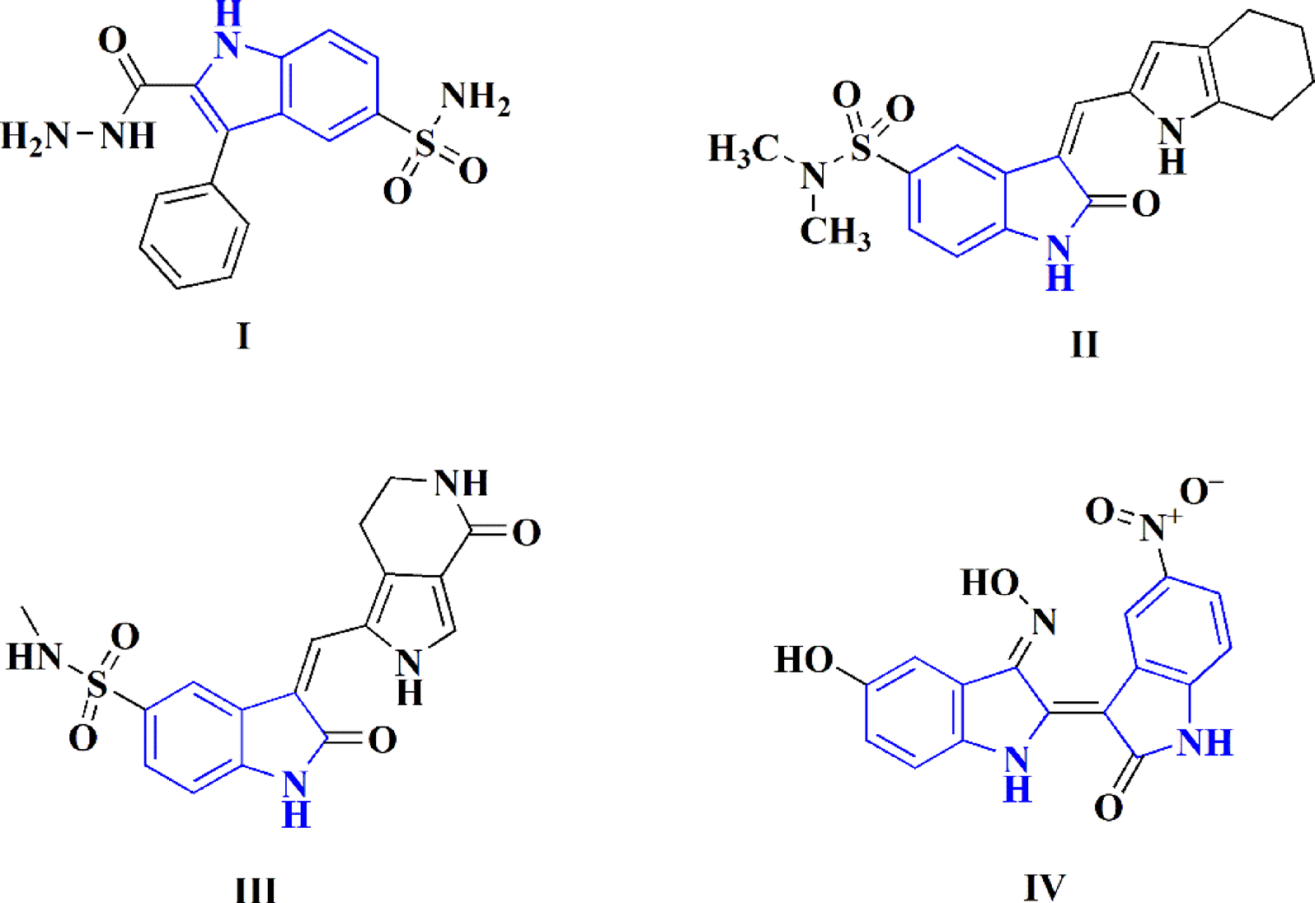
Some investigational and experimental indole-containing compounds

Several structurally diverse indole-based structures like indole-2-carboxamides, 3-hydrazonoindolin-2-ones and oxindole derivatives have been reported as cyclin-dependent kinase 2 inhibitors that can fight cancer [20–23]. In addition, marketed and investigational drugs comprising indole and sulfonyl functionalities, such as Zafirlukast (Fig. 1) and 2-(hydrazinocarbonyl)-3-phenyl-1 H-indole-5-sulfonamide (**I**), indicate the therapeutic significance of combining these pharmacophores [24, 25]. Previous investigations also verified highly potent CDK2-targeting indolinone derivatives, as the pyrrolyllactone indolinone compound **III** (IC50 = 0.009 µM) (Fig. 2) reported by Xiaoyuan Li et al. [26], as well as AMG-130 (**IV**), a clinically investigated indirubin-derived multi-CDK inhibitor indicating efficiency in imatinib-resistant chronic myeloid leukemia [27]. Collectively, these findings indicate that the indole core, particularly when functionalized with sulfonamide or structurally related bioactive moieties, represents a privileged scaffold for CDK2 inhibition and anticancer activity. Therefore, our designed compounds build upon these precedents to explore structurally optimized indole-based hybrids as potential CDK2 inhibitors with enhanced anticancer potential.

On the other hand, sulfonyl-containing derivatives are pharmacologically active agents that elicit a wide range of biological activities (i.e., anticancer, antimalarial, antimicrobial, and antiviral activities) [28–36]. It is worth noting that many anticancer activities of indole-sulfonamide derivatives were investigated [37]. For example, novel indole-3-sulfonamide-heteroaryl hybrids were developed as carbonic anhydrase inhibitors, which have induced anti-cancer potencies [38]. Novel sulfonamidoindole-based hydrazones were active against the tumor-associated carbonic anhydrase isoforms [39]. Also, indole-based sulfonamide derivatives act against α-glucosidase and α-amylase enzymes [40]. Some biologically important new series of indole-based sulfonamide derivatives [41], and another series of indoles bearing sulfonamides can potentially act as novel aromatase inhibitors for therapeutics [42].

In completing our programs in synthesizing aspirin analogs as potent anticancer agents [43, 44], and for further optimization, aspirin analogs are also utilized to be conjugated with the indoles to form novel active scaffolds. In this study, two series of indole derivatives were synthesized to explore complementary design strategies. The first series incorporated motifs bearing aspirin-like functional groups to investigate potential polar interactions within the ATP-binding pocket of CDK2. In the second series, alkanesulfonate substituents were introduced to explore functionalities that may enhance aqueous solubility, promote electrostatic interactions, and improve binding affinity.

**Fig. 3:**
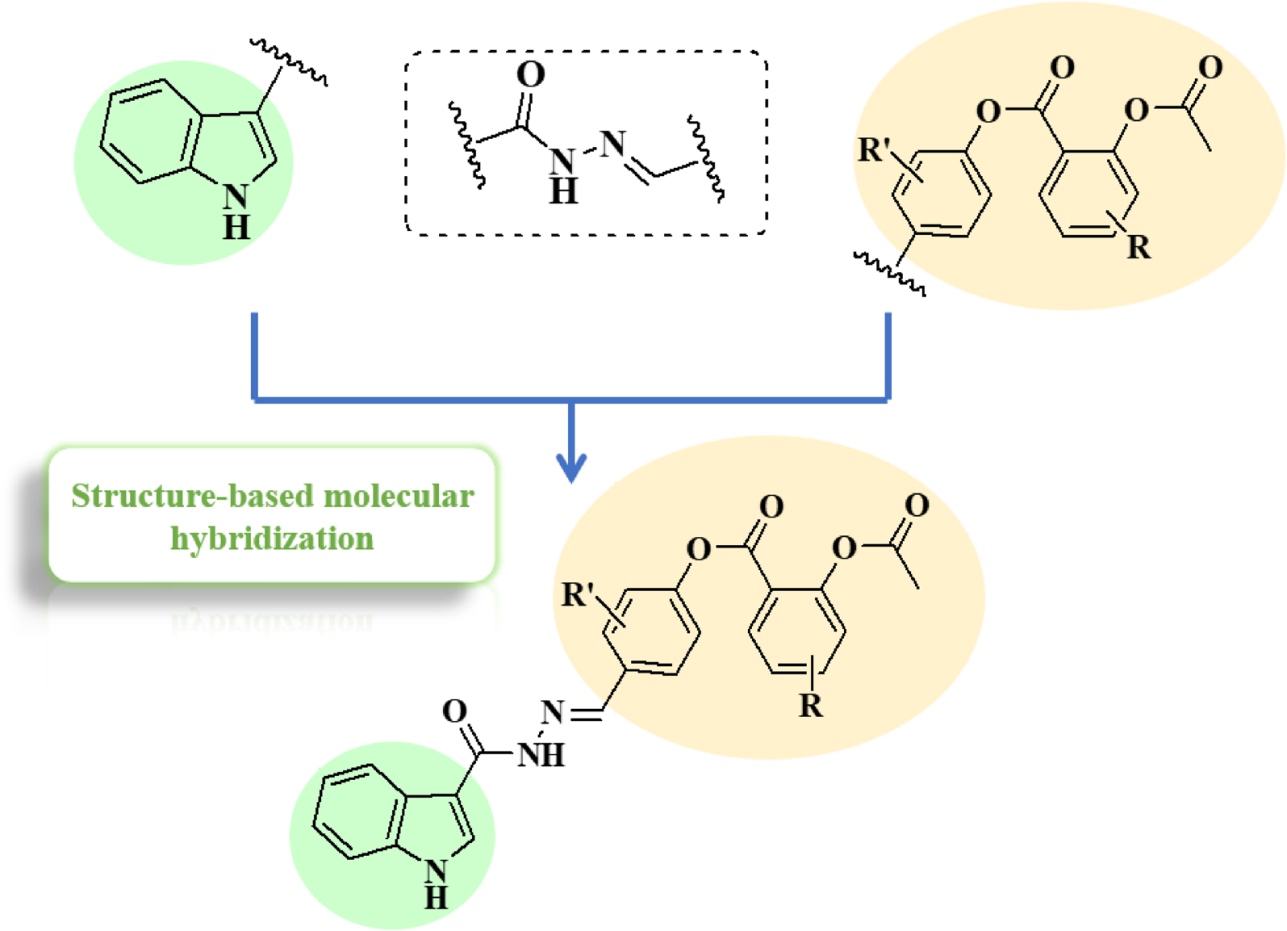
The rational design of the 1st approach generating the substituted indoles containing aspirin mimic moieties

**Fig. 4:**
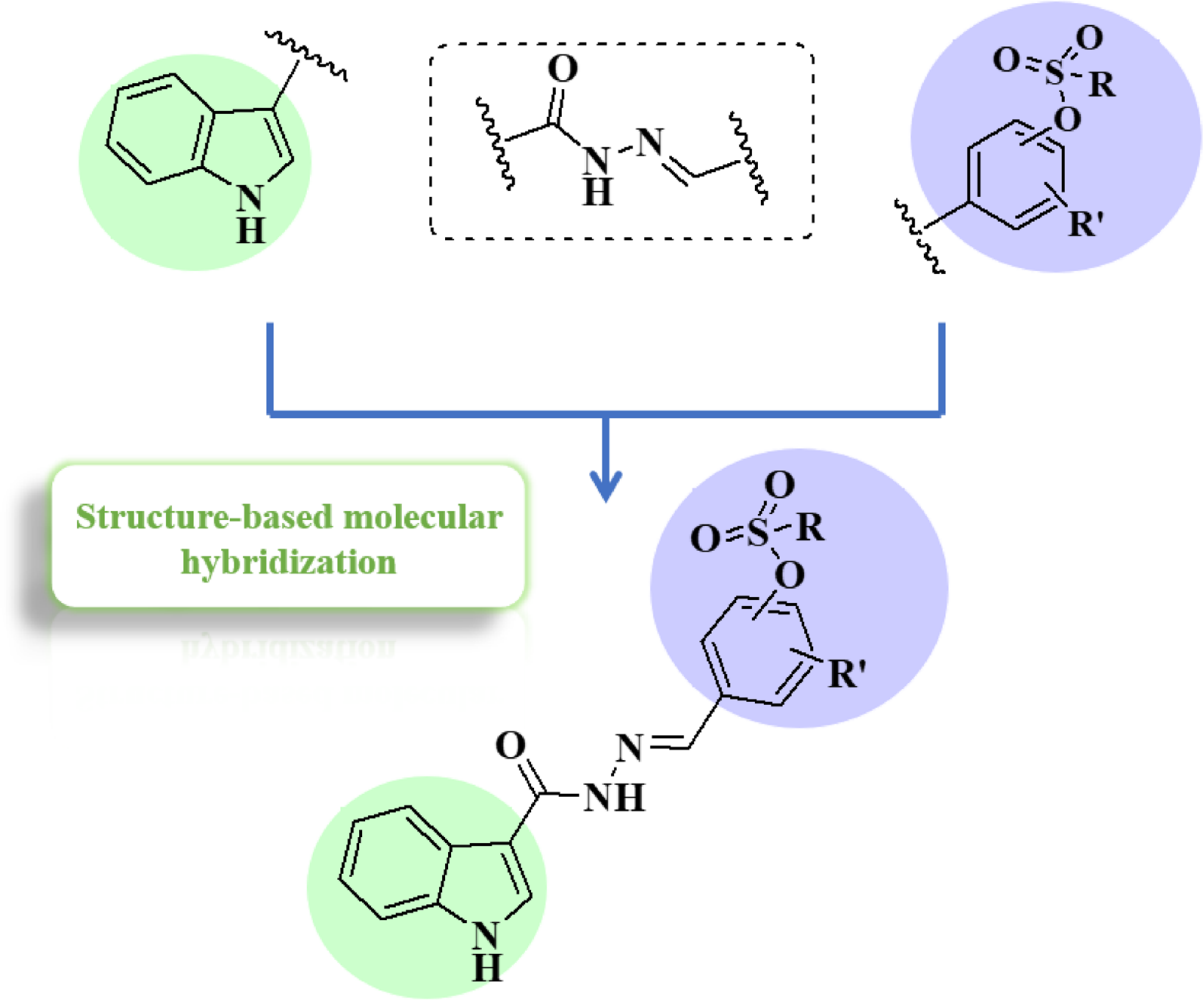
The rational design of the 2nd approach generating the indole-sulfonate analogs

From another perspective, hydrazides act as enabling linkers in anti-cancer drug discovery [45] and act as powerful tools in medicinal chemistry and possess many biological applications [46–49]. Thus, here we used the hydrazide moiety as a linker between the active pharmaophores. Combining all those active pharmacophores with the hydrazide linker generates the new potent structures (Figs. 3 and 4).

## Results and discussion

### Chemistry

**Scheme 1:**
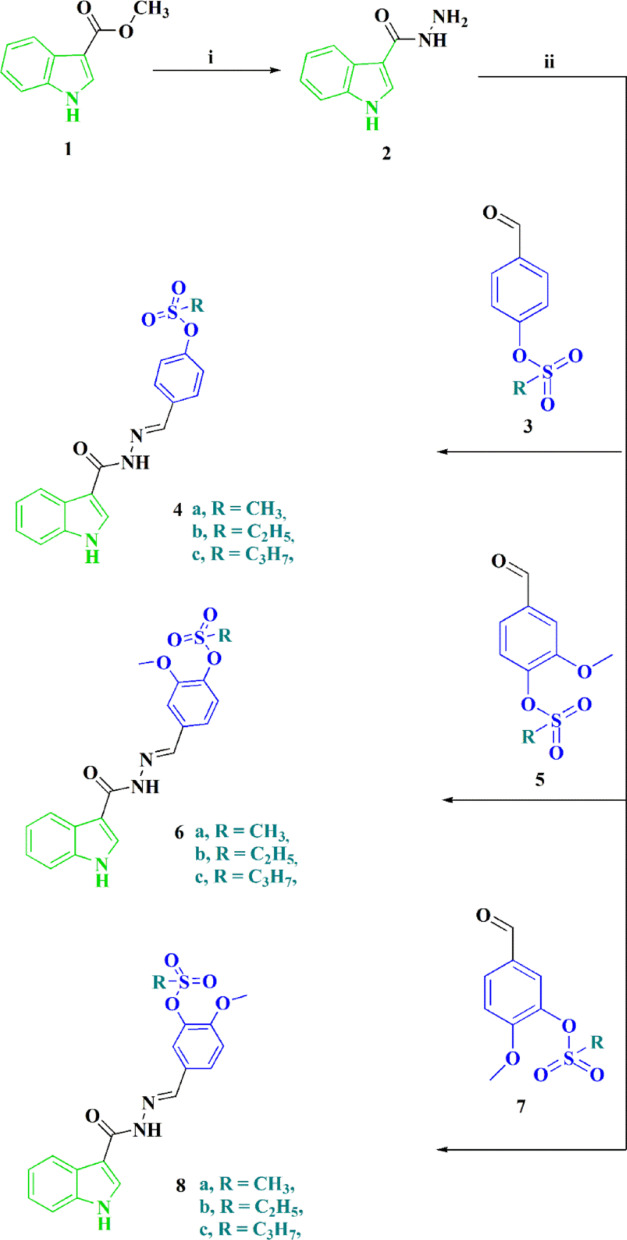
Synthetic route toward the preparation of indole-sulfonate analogs **4a-c**, **6a-c** & **8a-c**. Reagents & conditions: (i) Hydrazine hydrate, EtOH, 1h. (ii) AcOH/EtOH (1:2), reflux, 1h

The synthetic approach adopted for producing the novel targeted indole-sulfonate analogs **4a-c**, **6a-c** & **8a-c** started from methyl 1*H*-indole-3-carboxylate **1**. The latter compound was reacted under reflux with hydrazine hydrate in the presence of ethanol to afford 1*H*-indole-3-carbohydrazide (**2**). Indole carbohydrazide **2** was then allowed to react with various substituted alkane sulfonyl aryl aldehydes **3**, **5** & **7** to give the targeted indole-sulfonate analogs **4a-c**, **6a-c** & **8a-c** in high yields (Scheme 1). The structures of the newly synthesized compounds were confirmed using various spectroscopic techniques (^1^H NMR and ^13^C NMR), further supported by elemental analysis data.

**Scheme 2:**
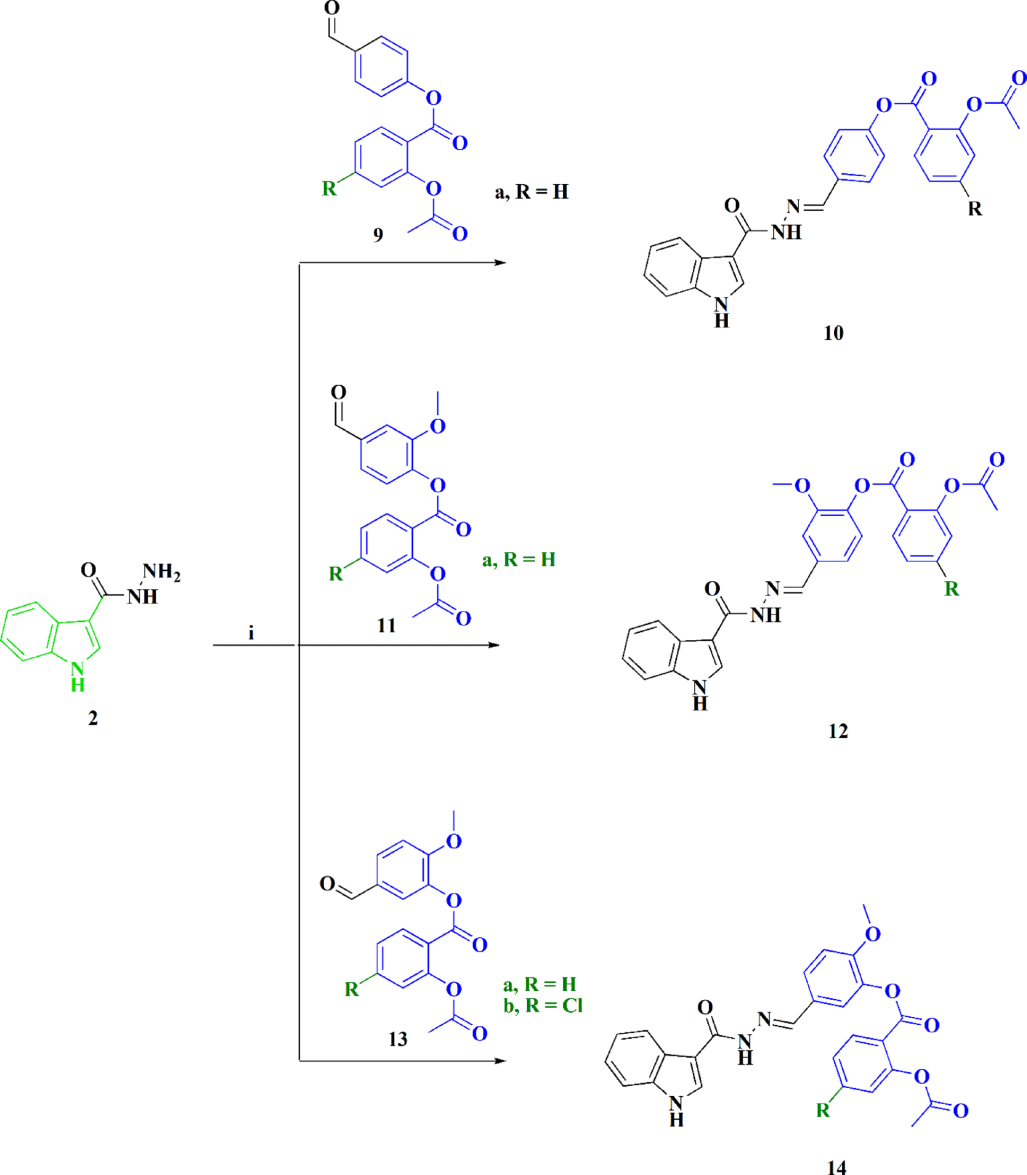
Synthetic route toward the preparation of indole-containing aspirin mimic moieties **10**, **12**, and **14**. Reagents & conditions: i) AcOH/EtOH (1:2), reflux, 1h

**Fig. 5:**
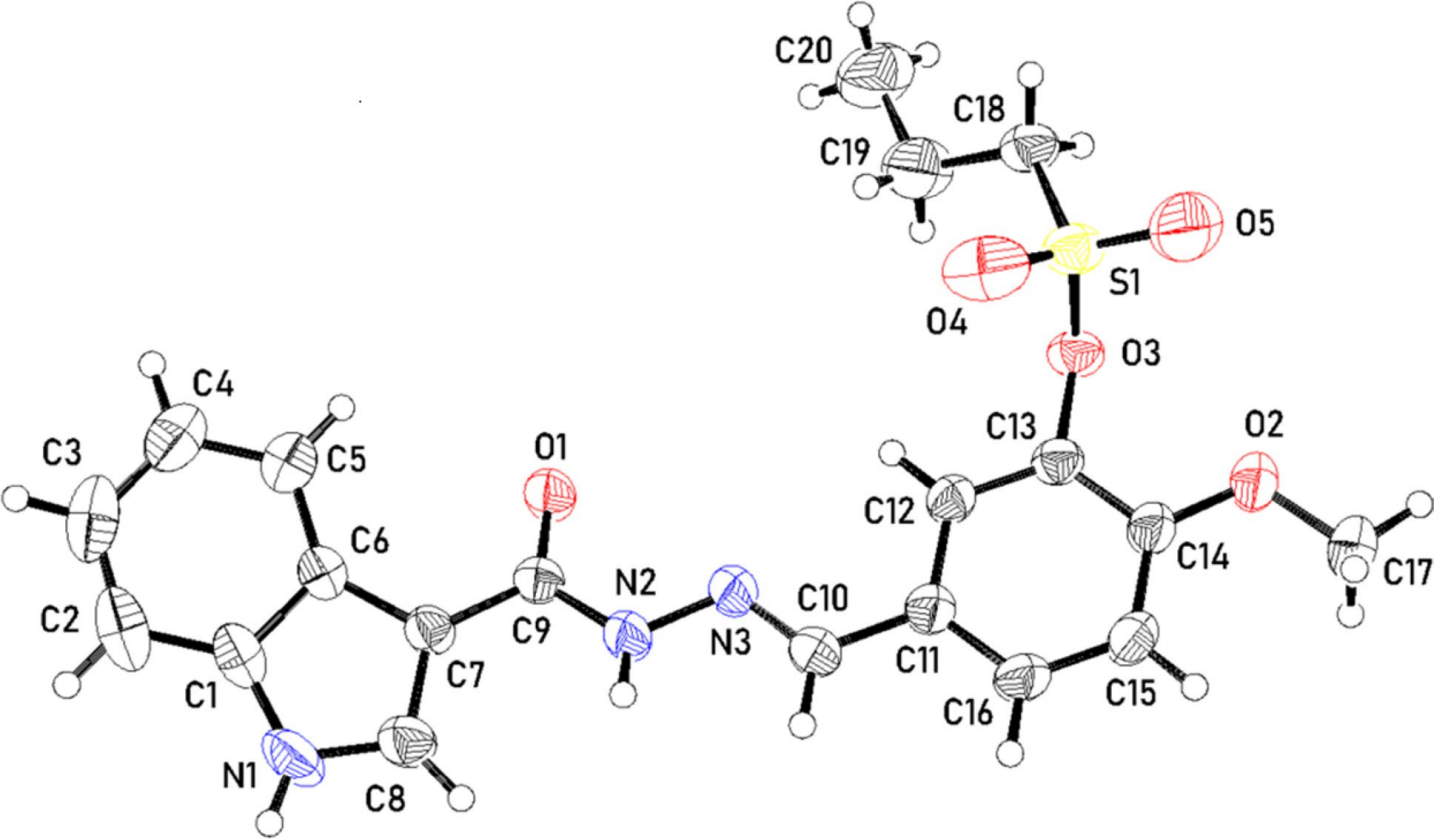
Molecular structure of compound **8c**. The International Union of Crystallography granted permission for the reproduction of the figure that illustrates the structure under an open-access license [50]

As a representative example of the indole-sulfonate analogs, the ^1^H NMR spectrum of compound **8c** revealed that the alkane sulfonate group (CH_2_CH_2_CH_3_) is represented by triplet peaks attributed to methyl groups, multiplet, and triplet signals depicting propyl side chains. The azomethine protons appear as a singlet broad signal at *δ*_H_ = 8.21 ppm, the NH protons are observed as a singlet peak at *δ*_H_ = 11.42 and 11.76 ppm; also, the aromatic signals appeared in the range from δ_H_ 7.13 to 7.64 ppm. ^13^C NMR exhibited signals at *δ*_C_ = 12.38 ppm, & 17.01 ppm for the aliphatic groups, at *δ*_C_ = 111.98-133.86 ppm representing aromatic carbons and at *δ*_C_ = 149.45 ppm for aromatic carbon bound to a heteroatom. Confirmation of the compound’s structure was verified via single-crystal X-ray diffraction, as demonstrated in Fig. 5 [50]. To explore the potential range of this approach, the 1*H*-indole-3-carbohydrazide (**2**) was reacted with aspirin mimic derivatives **9a**, **11** & **13a**,**b** to afford the substituted indoles containing aspirin mimic moieties **10**, **12**, & **14** (Scheme 2). The ^1^H NMR spectrum of compound **10** showed the existence of a singlet signal at δ_H_ = 3.44 ppm for the characteristic COCH_3_ protons, as well as singlets at δ_H_ = 10.50 & 11.79 ppm signifying NH signals in addition to the aromatic signals appearing in the range from δ_H_ 7.09 to δ 7.96 ppm. In addition, the azomethine protons appear as a singlet broad signal at *δ*_H_ = 8.24 ppm. The ^13^C NMR spectrum of compound **10** indicates the presence of the signal *δ*_*C*_ = 20.59 ppm for the methyl carbon, signals at *δ*_*C*_ = 164.82 ppm & 158.48 ppm reveal the carbonyl carbon of CH-CO, respectively. The spectral data and corresponding charts are provided in the Supplementary file, Figs. S1-S24.

### Biological studies

#### In vitro screening of the tumor cell growth inhibition at a single-dose assay (10 µM concentration)

At the National Cancer Institute, NIH, USA, estimates of the in vitro antiproliferative efficacy against the NCI 60-cell line panel were carried out using a selection of the Developmental Therapeutic Program (DTP) (www.dtp.nci.nih.gov) was used to select the final compounds. Computer-aided design-based compounds having drug-like modes of action ought to be given preference in the NCI screening program. The ability of the produced compounds to diversify the NCI small molecule compound collection has led to their selection for screening.

This screen’s approach uses 60 various human tumor cell lines representative of leukemia, colon, lung, brain, ovary, melanoma, prostate, kidney and breast cancers.

Compounds **4a-c**, **6c**, **8a-c**, **10**, **12**, **14a**, and **14b** (Figs. S25-S35) were selected by the NCI for in vitro screening using the disease-oriented human cancer cell panel. The modified chemotypes from this study were tested at an initial concentration of 10 µM in a one-dose (percent inhibition) assay across the NCI-60 cell lines.

The data are introduced as cell growth percent (GP) for every NCI cell line panel evaluation. In accordance with the methodology described by the US NCI [51–55], the data of the tested compounds were presented in Table 1, negative GP values indicate stronger growth inhibition.

Compound **4c** exhibited remarkably the lowest cell growth promotion against the renal cancer RXF 393 (GP = -6.26%), and leukemia RPMI-8226 (GP = 29.80%), while compound **4b** demonstrated reasonable activity on leukemia RPMI-8226 (GP = 55.20%), renal cancer RXF 393 (GP = 58.24%), and CNS cancer SF-539 (GP = 63.31%),

On renal cancer, RXF 393 compound **6c** showed the lowest cell growth promotion with GP = -14.11%, it displayed the lowest cell growth promotion against leukemia (GP = 43.37%), and CNS cancer SF-539 (GP = 45.49%).

Compound **8a**, (Fig. S29) showed remarkably lowest cell growth promotion against melanoma MDA-MB-435 (GP = -71.65%), CNS cancer SNB-75 (GP = -47.92%), ovarian cancer OVCAR-3 (GP = -28.56%), breast cancer MDA-MB-468 (GP = -14.75%), renal cancer RXF 393 (GP = -11.61%), and non-small cell lung cancer NCI-H522 (GP = -3.17%). Derivative **8c** disclosed moderate activity towards leukemia RPMI-8226, with cell growth promotion with GP = 41.51%, renal cancer RXF 393 (GP = 55.39%), and breast cancer T-47D (GP = 64.01%).

Whereas compound **8b** showed the lowest cell growth promotion against melanoma MDA-MB-435 with GP = -25.71%, it also showed remarkably the lowest cell growth promotion against CNS cancer SNB-75 (GP = -13.16%), ovarian cancer OVCAR-3 (GP = 5.42%), and leukemia HL-60(TB) (GP = 2.36%). On the other hand, derivative **14b** (fig. S35) displayed the lowest cell growth promotion against leukemia SR (GP = 52.92%), Melanoma MDA-MB-435 (GP = 59.30%), Breast cancer MCF7 (GP = 64.56%), and Renal cancer CAKI-1 (GP = 64.65%).

#### Evaluation of in vitro cytotoxic activity of the compounds against the NCI-60 human cancer cell line panel at five-dose levels

**Fig. 6:**
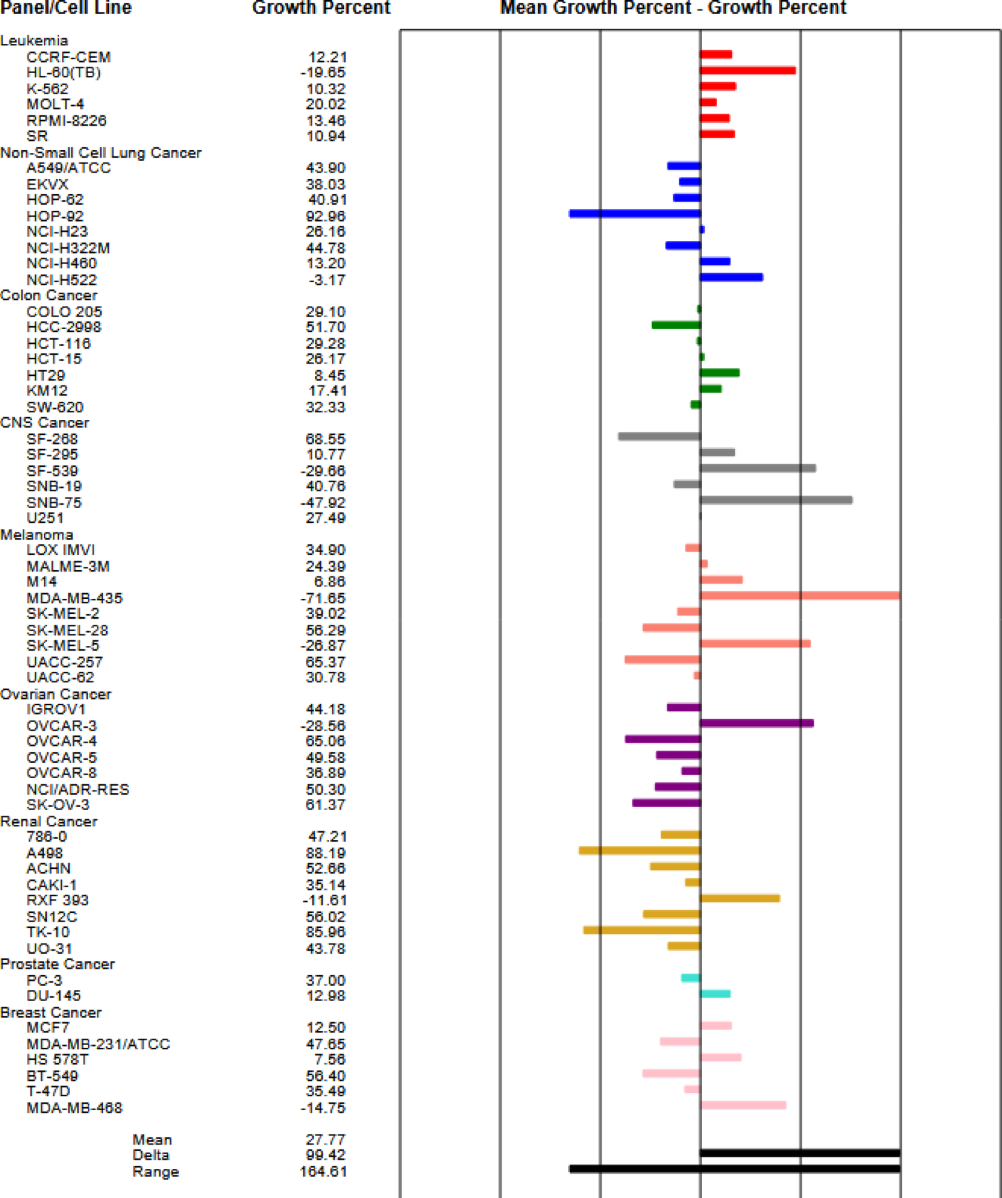
Anti-cancer activity results per single dose assay (10 *µ*M concentration) as percent cell growth of **8a** after 48 h, using sulforhodamine B (SRB) assay

Compound **8a** exhibited potent anticancer activity against a panel of over 60 tumor cell lines (Fig. 6), with GI_50_ values ranging from − 4.50 *µ*M to -6.45 *µ*M at sub-micromolar concentrations. The cell lines exhibiting the highest sensitivity were leukemia (SR), melanoma (MDA-MB-435), CNS Cancer (SNB-75), ovarian cancer (OVCAR-3), and non-small cell lung cancer (NCI-H522) with GI_50_ values of -6.45, -6.42, -6.31, -5.96, and − 5.87 mM, respectively, compound **8c** also demonstrated superior cytotoxic activity against melanoma (MA-MB-435) with TGI values of -5.82*µ*M. Furthermore, TGI values of -5.43, -5.28, -5.05, -5.01, -4.97, and − 4.94 *µ*M were observed against CNS cancer, Non-small cell lung cancer NCI-H522, renal cancer RXF 393, Ovarian cancer OVCAR-3, leukemia HL-60 (TB), and breast cancer MDA-MB-468 cell lines, respectively.

Alternatively, lethal impact (LC_50_) values were more than − 4,00 log_10_ against the cancer cell lines. Moderate cytotoxicity was established against melanoma, particularly the SK-MEL-5 cell line, revealing GI_50_ value = -5.65 *µ*M, TGI values = -5.10 *µ*M, and LC_50_ value = -4.48 log_10_.

**Fig. 7:**
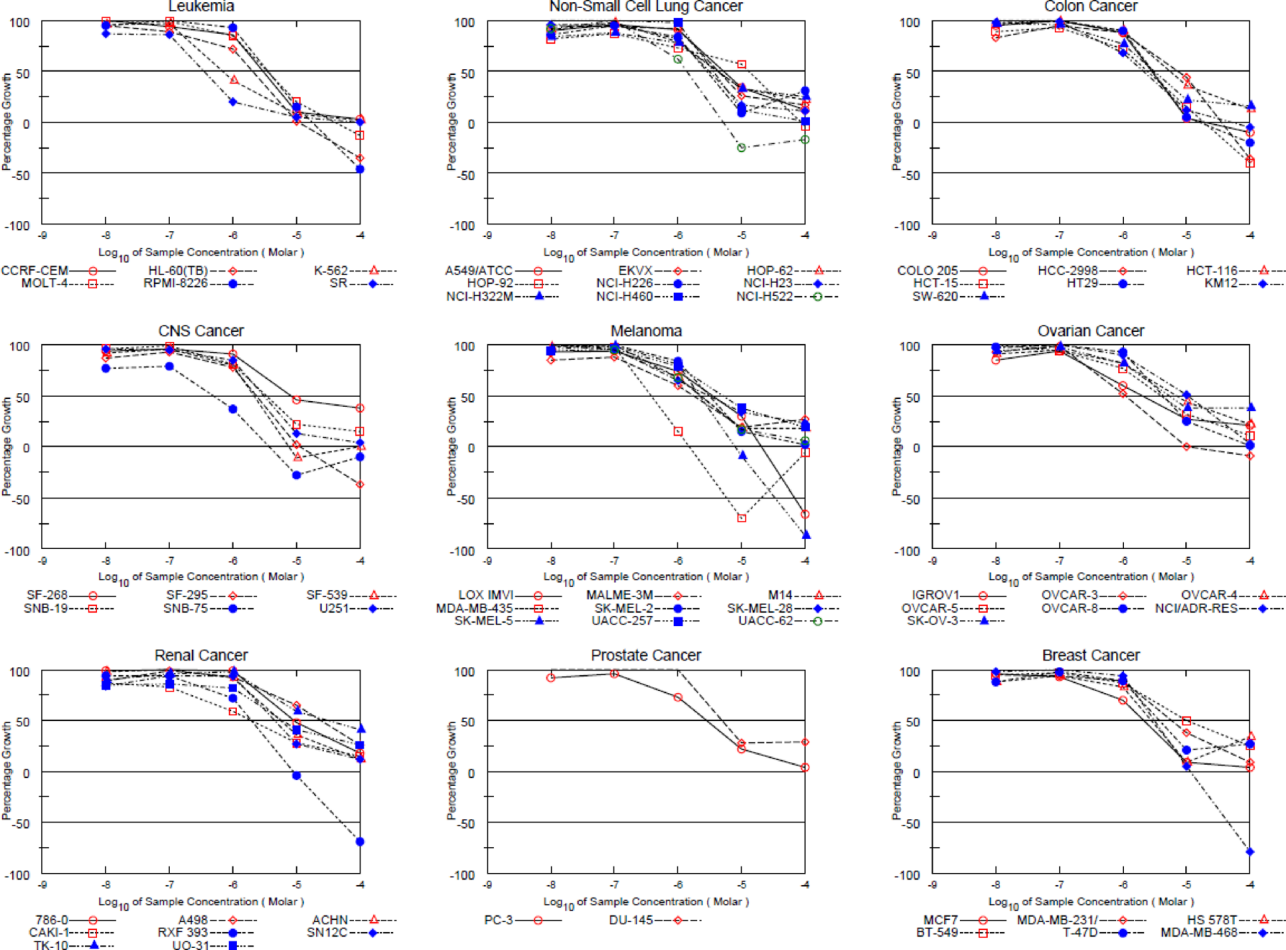
Graphical representation of the growth inhibition of **8a** across five-dose concentrations (0.01, 0.1, 1, 10, & 100 *µ*M) after 48 h, evaluated using SRB assay at NCI

Additionally, renal cancer RXF 393 revealing GI_50_ value = -5.71 *µ*M, TGI values = -5.05 *µ*M, and LC_50_ value = -4.29 log_10_. Also, breast cancer MDA-MB-468 indicated GI_50_ value = -5.51 *µ*M, TGI values = -4.94 *µ*M, and LC_50_ value = -4.35 log_10_.The GI_50_, TGI, and LC_50_ (in *µ*M) values against subpanel cell lines are displayed in Fig. 6. The five dose response curves of **8c** are shown in Fig. 7 & Figs. (S38 and S39).

**Table 1:** The results of the tested compounds in terms of percent growth promotion (GP)

Sample	4a	4b	4c	6c	8a	8b	8c	10a	12a	14a	14b
Cell line											
Leukemia											
CCRF-CEM	105.96	87.95	71.76	74.62	12.21	35.65	66.66	76.99	84.92	58.06	86.13
HL-60(TB)	105.67	89.56	82.25	84.02	-19.65	2.36	49.46	72.62	100.97	74.43	84.61
K-562	103.72	80.46	61.51	75.40	10.32	18.69	79.14	85.19	98.86	34.53	65.45
MOLT-4	107.51	86.87	59.17	64.01	20.02	51.57	87.15	85.47	119.25	75.02	107.24
RPMI-8226	107.70	55.20	29.80	43.37	13.46	41.85	41.51	87.37	91.28	0.76	63.37
SR	100.36	62.31	51.65	78.36	10.94	16.02	69.25	85.83	95.54	12.14	52.92
Non-small cell lung cancer											
A549/ATCC	103.85	90.98	73.03	90.82	43.90	58.58	91.54	80.32	97.98	70.89	91.30
EKVX	93.20	102.57	84.75	74.58	38.03	61.75	82.37	95.35	95.88	60.62	78.39
HOP-62	92.03	93.63	79.32	78.55	40.91	54.30	93.56	83.85	100.56	67.75	83.10
HOP-92	104.33	122.47	110.92	98.15	92.96	94.81	116.92	99.48	89.86	62.86	89.36
NCI-H23	99.77	103.33	88.32	85.77	26.16	51.97	99.95	80.28	94.72	83.14	94.04
NCI-H322M	99.98	98.35	83.60	82.23	44.78	75.18	99.77	90.41	104.75	91.07	92.70
NCI-H460	109.12	88.87	71.84	96.26	13.20	26.28	101.06	93.44	102.54	78.35	94.62
NCI-H522	97.11	100.57	75.50	93.28	-3.17	27.08	85.91	92.65	85.20	78.20	91.05
Colon cancer											
COLO 205	109.72	101.29	84.21	87.17	29.10	57.85	112.04	110.33	114.49	93.84	110.34
HCC-2998	101.10	78.40	67.34	86.09	51.70	59.26	88.04	109.63	106.62	71.20	94.07
HCT-116	106.92	100.84	87.43	87.23	29.28	25.02	94.10	75.51	104.55	51.87	90.97
HCT-15	104.61	72.69	49.19	70.94	26.17	33.33	74.46	98.21	100.17	23.96	68.13
HT29	105.27	93.53	58.24	67.83	8.45	17.59	98.65	104.58	106.78	52.75	100.14
KM12	100.80	73.06	60.29	94.94	17.41	27.03	76.15	94.79	101.22	38.85	79.08
SW-620	102.15	97.71	92.13	103.54	32.33	40.29	102.04	96.88	101.03	87.81	99.55
CNS cancer											
SF-268	86.45	83.77	66.35	96.24	68.55	61.69	107.53	87.80	100.58	78.89	103.50
SF-295	94.08	80.72	60.07	61.04	10.77	24.91	81.77	99.49	98.52	95.02	98.70
SF-539	96.54	63.31	45.69	45.49	-29.66	30.59	79.78	84.78	98.39	70.63	86.67
SNB-19	99.22	100.50	83.80	88.79	40.76	59.17	99.82	92.47	101.68	79.82	90.78
SNB-75	78.61	94.10	59.10	70.91	-47.92	-13.16	95.57	94.91	100.62	91.47	96.53
U251	104.83	94.05	82.03	81.91	27.49	44.61	91.46	82.05	102.20	65.47	95.72
Melanoma											
LOX IMVI	96.10	83.14	53.75	78.36	34.90	35.48	79.55	83.82	92.31	14.90	77.44
MALME-3 M	99.12	88.66	75.64	76.18	24.39	39.21	90.62	90.81	92.67	74.90	92.31
M14	111.75	101.44	86.65	96.50	6.86	31.33	85.90	90.60	100.41	52.48	89.58
MDA-MB-435	104.79	95.63	77.87	98.27	-71.65	-25.71	89.06	98.61	102.33	30.40	59.30
SK-MEL-2	103.74	108.75	86.01	83.46	39.02	27.69	111.41	115.45	115.67	96.41	106.32
SK-MEL-28	107.92	93.25	79.05	87.13	56.29	63.95	102.03	106.77	107.76	77.49	100.44
SK-MEL-5	98.95	93.22	77.54	77.10	-26.87	17.28	90.27	89.70	98.55	31.33	85.01
UACC-257	98.87	101.64	87.27	97.42	65.37	76.92	104.76	90.61	100.90	83.72	87.90
UACC-62	95.62	104.80	89.19	89.41	30.78	28.03	94.53	85.87	87.59	77.72	86.33
Ovarian cancer											
IGROV1	102.89	101.73	94.10	96.23	44.18	55.55	107.96	87.21	102.54	68.80	78.45
OVCAR-3	102.56	101.74	94.52	117.28	-28.56	5.42	111.62	97.36	115.56	86.92	104.63
OVCAR-4	94.84	84.78	69.23	90.24	65.06	69.33	88.28	89.92	98.49	77.56	88.98
OVCAR-5	105.95	98.42	84.32	110.40	49.58	86.43	106.25	104.21	104.03	85.52	104.33
OVCAR-8	101.43	94.29	87.30	98.04	36.89	68.83	91.39	78.41	98.50	86.88	92.88
NCI/ADR-RES	103.20	97.40	78.55	87.46	50.30	71.18	85.64	84.08	93.31	66.90	86.27
SK-OV-3	97.42	107.36	75.08	81.00	61.37	81.38	117.36	88.77	100.04	79.08	82.31
Renal cancer											
786-0	102.08	91.73	81.61	76.36	47.21	66.47	95.48	108.54	113.16	107.40	109.53
A498	103.37	110.02	107.9	114.21	88.19	95.38	117.23	123.15	116.63	116.33	126.24
ACHN	100.54	101.66	86.10	85.63	52.66	64.63	99.30	95.06	101.56	85.88	95.41
CAKI-1	77.97	72.90	62.30	78.32	35.14	42.25	95.29	79.28	91.78	54.07	64.65
RXF 393	112.31	58.24	-6.26	-14.11	-11.61	50.46	55.39	90.64	104.24	74.27	89.26
SN12C	104.58	91.87	82.47	91.33	56.02	71.59	88.28	92.39	95.28	88.82	93.00
TK-10	110.38	122.63	101.64	113.01	85.96	95.04	114.85	121.02	123.12	129.20	134.63
UO-31	83.27	75.10	78.76	79.20	43.78	54.88	81.90	77.01	82.60	65.61	67.13
Prostate cancer											
PC-3	109.19	99.35	82.43	81.48	37.00	53.04	82.19	88.58	93.62	54.54	78.18
DU-145	104.02	96.49	81.58	108.60	12.98	84.65	101.06	101.24	111.12	88.64	109.45
Breast cancer											
MCF7	89.91	80.29	70.81	75.37	12.50	20.23	79.55	84.85	84.36	49.56	64.56
MDA-MB-231/ATCC	92.47	85.69	70.17	80.26	47.65	59.52	93.61	84.49	88.91	53.01	71.99
HS 578T	92.28	93.54	73.02	64.75	7.56	32.65	83.25	86.30	90.87	61.92	82.40
BT-549	116.63	113.61	94.95	76.51	56.40	43.05	77.11	99.85	107.98	71.15	91.22
T-47D	94.23	80.31	59.65	72.72	35.49	46.25	64.01	93.74	96.08	63.55	79.49
MDA-MB-468	109.53	97.64	67.02	84.41	-14.75	36.07	97.95	82.64	101.33	59.03	67.20

#### In vitro CDK-2 inhibitory evaluation of 8a derivative

The promising derivative **8a** was evaluated for CDK-2 inhibitory activity as previously reported [56, 57]. The luminescent detection technique was used with the Bioscience CDK-2/cyclin A, assay Kit in the presence of Kinase-Glo Max detection reagent to detect IC_50_ values (*µ*M) for the tested derivative and roscovitine as a reference drug, as accomplished in Table 2. Interestingly, the tested compound (**8a**) showed approximately 2-fold higher potency than the reference drug, with an IC_50_ equal to 0.48 *µ*M, while that of roscovitine was 1.11 *µ*M. These findings confirmed that one of the mechanisms of the anti-proliferative activity of compound **8a** is via the inhibition of CDK-2. Although the synthesized compounds showed promising in vitro CDK2 inhibitory activity and antiproliferative effects in cancer cell lines, the intracellular mechanism of action remains to be fully elucidated. In order to verify direct target interaction and pathway regulation, future studies will focus on comprehensive cellular and molecular studies.

**Table 2:** In vitro CDK-2/ cyclin A inhibitory activity of the promising derivative **8a** and roscovitine as a standard drug

Compound	IC_50_ (mean ± SEM) (µM)
	CDK-2/cyclin A
**8a**	0.48 ± 0.006
Roscovitine	1.11 ± 0.014

IC_50_: Compound concentration necessary to inhibit the enzyme activity by 50%, SEM: Standard error mean; each value is the mean of three values

#### In vitro cytotoxic effect of 8a on human skin fibroblast (BJ-1) normal cell line

The compound **8a**, selected by NCI for five doses, was tested for in vitro cytotoxicity on normal skin fibroblasts (BJ-1) using the MTT assay, as previously reported [58], with DMSO serving as the negative control. The IC_50_, or the concentration causing 50% of normal cell death, was 66.3 *µ*g/mL, indicating that our compound can be considered safe up to this concentration. This suggests that compound **8a** has a wide safety margin compared to its IC_50_s against both cancer cell lines and CDK2.

#### In silico study of all the newly synthesized derivatives

##### The drug likeness and ADMET prediction studies

All of the final fourteen compounds were tested for in silico study, comprising Drug likeness and absorption, distribution, metabolism, and excretion (ADME) prediction employing the Swiss ADME website (http://www.swissadme.ch) [59] as well as the ProTox.3 server [60] for the toxicity prediction (including predicted LD_50_, organ and endpoint toxicity) as illustrated in Tables 2, 3 and 4. All of the tested compounds adhered to Pfizer Lipinski’s rule without any violation except for compound **14b**, which has a molecular weight slightly higher than 500, as depicted in Table 3. According to Ali’s water solubility classification, it was found that the compounds with phenylalkanesulfonate moiety **4a-c**, **6a-c**, **8a-c** were of moderate aqueous solubility, while the rest of the compounds were of poor water solubility. All of the tested derivatives successfully passed the PAIN filter, which measures the presence of molecular moieties that might interfere with any biological activity.

**Table 3:** The predicted Swiss ADME website for the drug likeness (Lipinski’s rule) and some important physicochemical properties for all the synthesized derivatives

Cpd	Lipiniski rules							violation	Water solubility		PAIN
ID	MW	Heavy atoms	Rotatable bonds	H-bond acceptors	H-bond donors	TPSA	MLOGP	yes or 0	Ali Log S	Ali Class	Alerts
	≤ 500 Lipiniski	20 ≤ atoms ≤ 70	≤ 9	≤ 10	≤ 5		lipophilicity ≤ 4.15				
**4a**	357.38	25	6	5	2	109	1.79	0	-4.86	Moderate	0
**4b**	371.41	26	7	5	2	109	2.03	0	-4.82	Moderate	0
**4c**	385.44	27	8	5	2	109	2.26	0	-5.36	Moderate	0
**6a**	387.41	27	7	6	2	118.23	1.51	0	-4.6	Moderate	0
**6b**	401.44	28	8	6	2	118.23	1.74	0	-4.98	Moderate	0
**6c**	415.46	29	9	6	2	118.23	1.97	0	-5.52	Moderate	0
**8a**	387.41	27	7	6	2	118.23	1.51	0	-4.6	Moderate	0
**8b**	401.44	28	8	6	2	118.23	1.74	0	-4.98	Moderate	0
**8c**	415.46	29	9	6	2	118.23	1.97	0	-5.52	Moderate	0
**10a**	441.44	33	9	6	2	109.85	3.11	0	-6.13	Poorly	0
**12a**	471.46	35	10	7	2	119.08	2.53	0	-6.3	Poorly	0
**14a**	471.46	35	10	7	2	119.08	2.8	0	-6.3	Poorly	0
**14b**	505.91	36	10	7	2	119.08	3.27	1	-6.95	Poorly	0

*MW: Molecular weight ≤ 500; Heavy atoms: 20 ≤ atoms ≤ 70; Rotatable bonds ≤ 9; lipophilicity: MlogP < 4.15; HB acceptor: Hydrogen bond acceptor ≤ 10; HB Donor: Hydrogen bond donor ≤ 5; TPSA (Topological polar surface area) 20–130 A^◦2^. PAIN: Pan-assay interference filter

**Table 4:** The prediction of some Swiss (ADME) pharmacokinetic parameters, such as gastrointestinal absorption, Blood-Brain Barrier permeability, P-glycoprotein substrate, the cytochrome P450 inhibitory profile, the skin permeability, and oral bioavailability score

compound	Swiss ADME			Inhibitors of CYP450					Skin permeability	Oral
	GI absorption	BBB permeant	Pgp substrate	CYP1A2	CYP2C19	CYP2C9	CYP2D6	CYP3A4	log Kp (cm/s)	Bioavailability
**4a**	High	No	No	No	No	Yes	No	No	-6.41	0.55
**4b**	High	No	No	No	Yes	Yes	No	No	-6.53	0.55
**4c**	High	No	No	No	Yes	Yes	No	Yes	-6.24	0.55
**6a**	High	No	No	No	No	Yes	No	Yes	-6.91	0.55
**6b**	High	No	No	No	Yes	Yes	No	Yes	-6.73	0.55
**6c**	High	No	No	No	Yes	Yes	No	Yes	-6.45	0.55
**8a**	High	No	No	No	No	Yes	No	No	-6.91	0.55
**8b**	High	No	No	No	Yes	Yes	No	Yes	-6.73	0.55
**8c**	High	No	No	Yes	Yes	Yes	No	Yes	-6.45	0.55
**10a**	High	No	No	No	Yes	Yes	No	No	-6.07	0.55
**12a**	High	No	No	No	Yes	Yes	No	Yes	-6.27	0.55
**14a**	High	No	No	No	Yes	Yes	No	Yes	-6.27	0.55
**14b**	Low	No	No	No	Yes	Yes	No	Yes	-6.03	0.55

*P-gp: permeability to glycoprotein, the higher the negative log Kp, the more the skin impermeability; the score of bioavailability ≥ 0.55 indicates a good orally bioavailable compound

As illustrated in Table 4, all of the synthesized derivatives were expected to have a high absorption rate in the gastrointestinal tract, except that compound **14b** had a low GIT absorption rate. Favorably, all of the tested compounds were predicted not to cross the blood-brain barrier, so they had fewer CNS side effects and were not a substrate for the active efflux glycoprotein transporter across the cell membrane. The tested compounds’ inhibitory profile for some representative cytochrome P450 enzymes, which play a crucial role in the metabolism and excretion processes. All of the compounds were assumed to be orally bioavailable and to have moderate skin permeability.

**Table 5:**
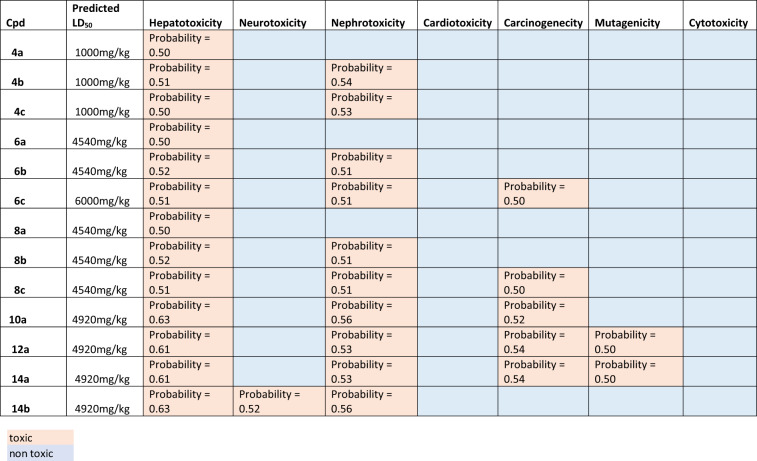
Protox 3 predictions of median lethal dose (LD_50_), organ toxicity (including liver, neuron, kidney, and heart), and endpoint toxicity (including carcinogenicity, mutagenicity, and cytotoxicity)

As presented in Table 5, the Protox-3 toxicity profile of the tested compounds indicated that the predicted median lethal dose (LD_50_) was in the range of 4000 mg/kg, except for **6c**, which was 6000 mg/kg, and **4a-c**, which were 1000 mg/kg. With a probability range of 0.5–0.6, all of the tested derivatives were predicted to be hepatotoxic and nephrotoxic except **4a**, **6a**, and **8a**. All of the tested compounds were predicted to be safe for the heart and neurons except **14b**, which was assumed to be neurotoxic with a probability of 0.52. On the other hand, concerning the endpoint toxicity, all of the examined compounds weren’t cytotoxic or mutagenic except **12a** and **14a**. Out of all the compounds, only five compounds (**6c**, **8c**, **10a**, **12a**, and **14a**) were expected to be carcinogenic with an approximate probability of 0.5. To sum up, when we focused on the promising compound **8a**, it was predicted as an orally bioavailable drug-like candidate with good ADMET properties.

##### Molecular modelling simulation study of the promising compound 8a in CDK-2

**Fig. 8:**
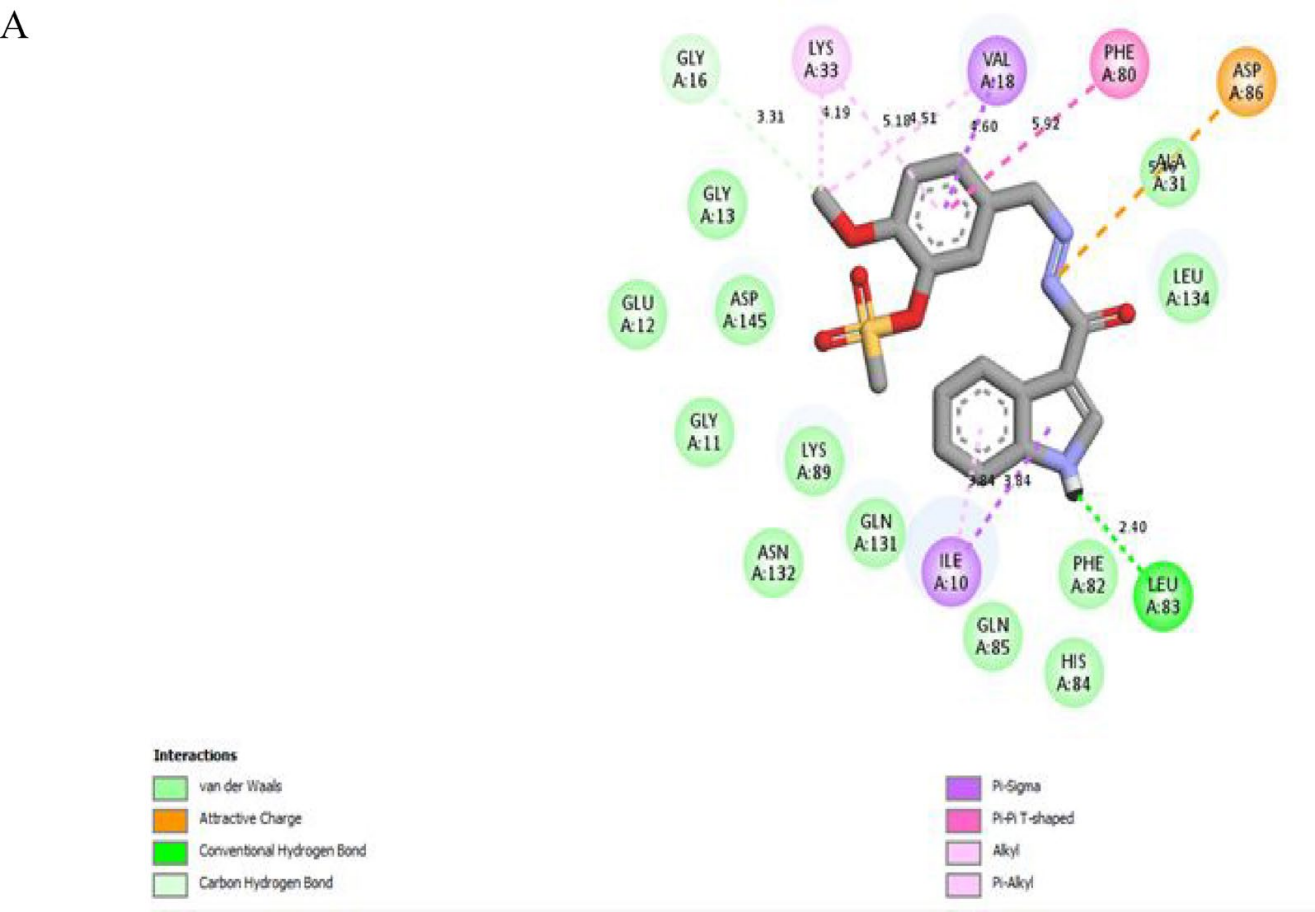

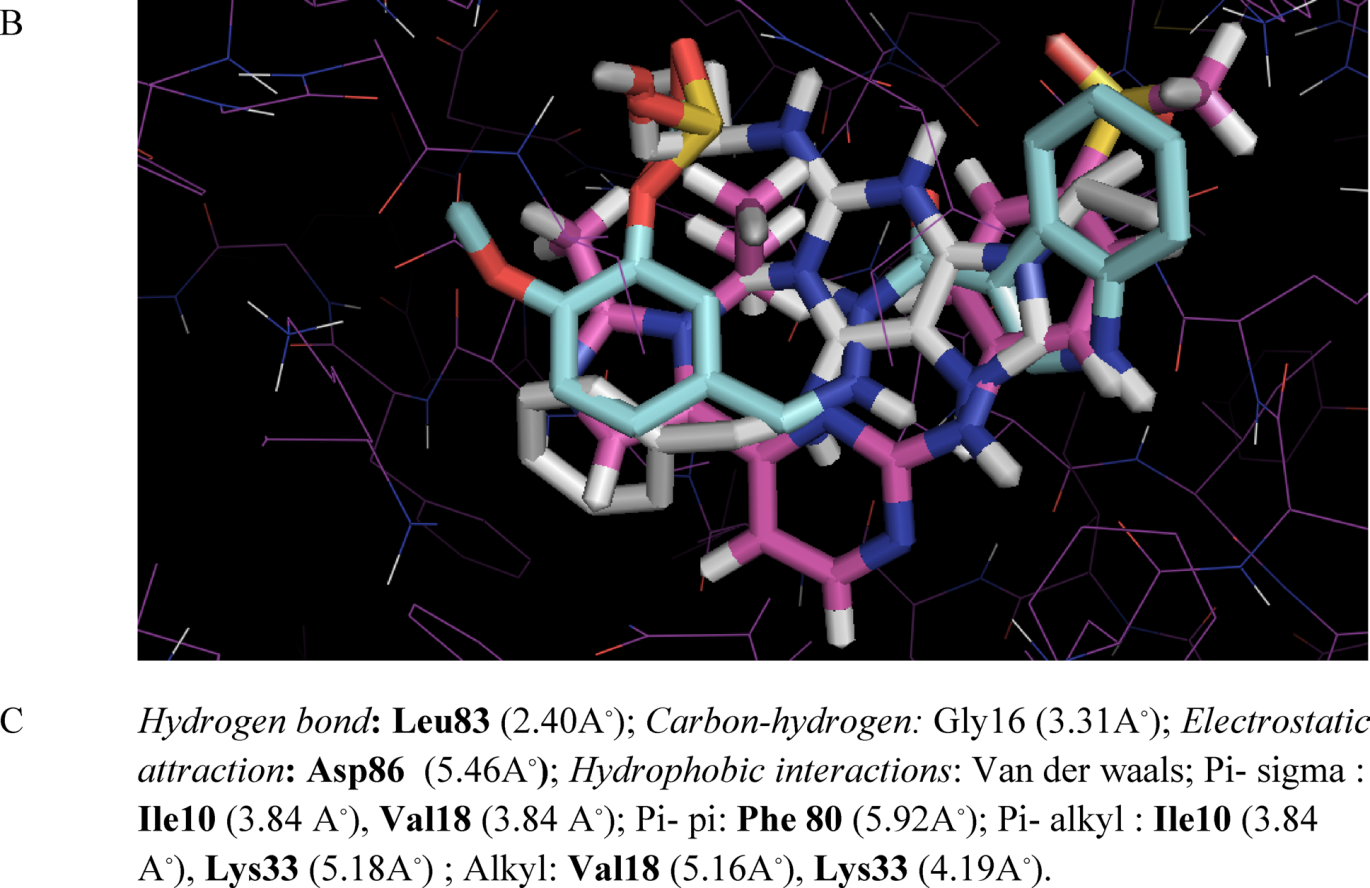
**A**: The predicted 2D-binding interactions of **8a** in the ATP-binding pocket (PDB code: 6GUH). **B**: The expected 3D-binding pose of **8a** (cyan) superimposed on the native ligand AZD5438 (pink) and roscovitine (white) in purple stick presented structure of CDK-2; **C**: Detailed binding interactions types and bond lengths, the bold interactions refer to common amino acid residues with the native ligand

To provide a more comprehensive analysis of the binding affinity and binding interactions, the promising compound **8a**, roscovitine, and the native ligand AZD 5438 were docked in the ATP-binding site of CDK-2 using AutoDock Vina integrated into the PyRx computational platform https://pyrx.sourceforge.io/ [61], in accordance with previously established methodologies [57, 62]. The three-dimensional crystal structure of the target CDK-2, co-crystallized with its respective native ligand inhibitor AZD 5438, was retrieved from the Protein Data Bank (PDB) https://www.rcsb.org under accession (PDB ID: 6GUH) [63]. This PDB file was of better resolution (1.5 A^◦^); therefore, it was selected to perform the docking study instead of (PDB ID: 3DDQ) [64] with roscovitine as a native ligand whose resolution was (1.8 A^◦^). After the protein preparation, energy minimization was carried out using the YASARA energy minimization server [65]. The docking study validation was reliable by re-docking of the native ligand AZD 5438 alongside the tested derivatives, yielding a root-mean-square deviation (RMSD) value of 0.3 Å. Two-dimensional (2D) and three-dimensional (3D) binding interactions were generated by *Biovia Discovery Studio 2021* https://discover.3ds.com. The results were presented in Table 6; Fig. 8.

**Table 6:** The binding affinity (kcal/mol) for the promising derivative **8a**, roscovitine, and the native ligand in CDK2

Compound	Binding affinity (Kcal /mol) CDK2
**8a**	-8.8
Roscovitine	-8.5
AZD 5438	-8.6

**Fig. 9:**
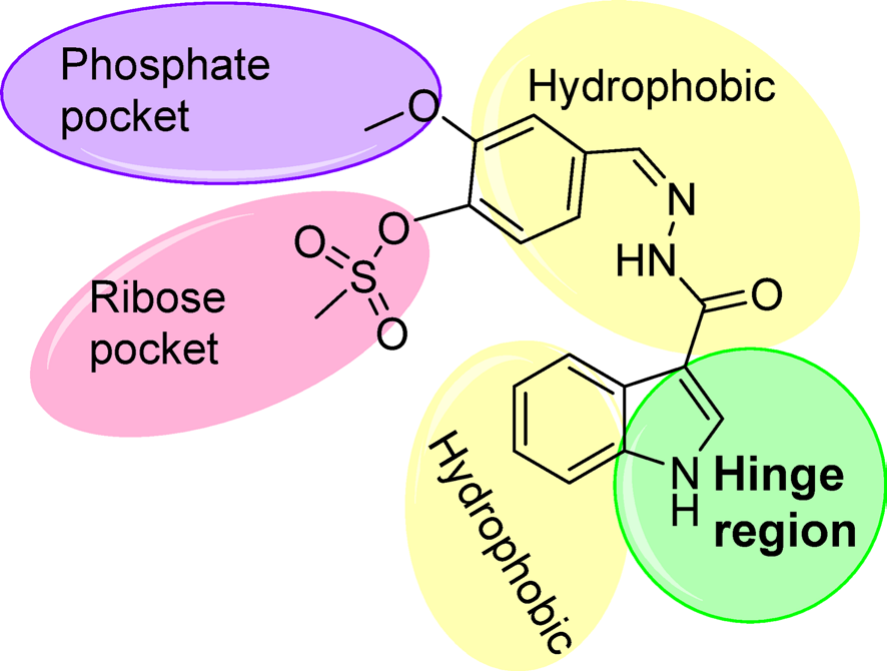
The schematic presentation of compound **8a**, highlighting the interaction in different binding regions in the ATP-binding pocket of CDK-2

Interestingly, the compound **8a** had the highest binding affinity (-8.8 kcal/mol) among roscovitine (-8.5 kcal/mol) and AZD5438 (-8.6 kcal/mol). On investigation of Fig. 8, it was predicted that the NH of the indole moiety of **8a** formed a hydrogen bond with Leu83 in the hinge region, which is an essential interaction that was reported for several CDK-2 inhibitors [66, 67]. Additionally, both the phenyl ring of indole and the phenyl ring attached to methylsulfonate fit into the hydrophobic regions of the ATP binding pocket, forming different hydrophobic interactions with Phe80, Val18, Lys33, and Ile10. It was noticed that **8a** made an electrostatic attraction with Asp86, unlike AZD 5438, which formed a hydrogen bond with the same residue. The terminal methoxy group formed an interaction with Gly16, the glycine-rich motif, which is a part of the phosphate binding pocket [68]. Finally, the methylsulfonate moiety of **8a** faced the ribose binding pocket with several hydrophobic Van der Waals interactions. All of the binding interactions of compound **8a** were with similar previously reported essential amino acid residues that confirmed the ATP-competitive inhibition of CDK-2, the 2D- binding interaction of Roscovitine and AZD 5438 were attached in supp. file S39, S40. As depicted in Fig. 8B, the indole-phenylmethanesulfonate Schiff base (**8a**) was superimposed with roscovitine and AZD in the binding pocket of CDK-2. A schematic presentation of **8a**, highlighting different binding regions of the ATP-binding pocket of CDK-2, was depicted in Fig. 9.

In brief, the docking study has succeeded in explaining the excellent in vitro CDK-2 inhibitory assay result of compound **8a**.

#### Structure-activity relationship (SAR) of compound **8a**

**Fig. 10:**
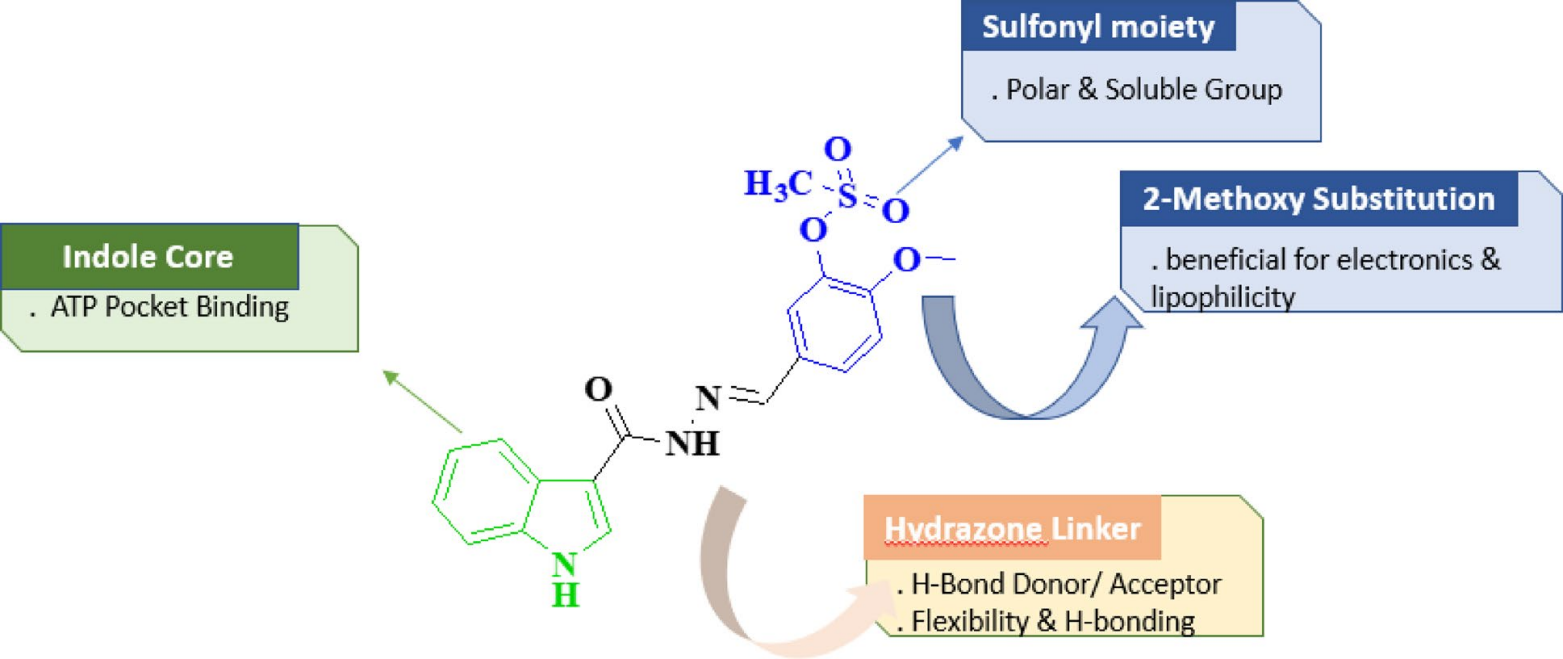
Structure-activity relationship of compound **8a**

According to the SAR study of 5-((2-(1 H-indole-3-carbonyl)hydrazono)methyl)-2-methoxyphenyl methanesulfonate) (Fig. 10), the indole core functions as the principal pharmacophore and contributes crucial π–π stacking and possible hinge-region hydrogen bonding, making it vital to preserve for CDK2 binding. Although stability optimization may be investigated in future analogs, the hydrazone linker’s dual hydrogen-bonding ability and conformational flexibility aid in orienting the pharmacophoric units into the ATP binding site. The 2-methoxy-substituted phenyl ring supports effective cellular permeability and biological activity by improving lipophilicity, electronic stability, and favorable molecular alignment. Lastly, the methanesulfonate moiety enhances solubility, adds polarity, and interacts at the solvent-exposed area. Its steric size provides an optimization handle for adjusting metabolic stability and efficacy. All of these characteristics suggest the compound’s potential as a promising CDK2-oriented anticancer scaffold as well as its broad-spectrum anticancer activity oriented in the NCI-60 panel. Its worthy to note that the acylhydrazone linker (-CONH–N = CH–), which offers both structural flexibility and functional diversity, is incorporated into the compounds. An electrophilic imine carbon (-CH = N–), a nucleophilic imine nitrogen with a lone pair (-N=), and an amino nitrogen (-NH–) with a weak acidic character are all present in this moiety [69]. These characteristics give the linker ambident electrophilic and nucleophilic properties, allowing it to take part in hydrogen-bond interactions that stabilize the active site of the enzyme. According to docking studies, the acylhydrazone moiety aids in the molecule’s ideal orientation in CDK2’s ATP-binding pocket by forming hydrogen bonds with residues in the hinge region, which helps explain the observed in vitro inhibitory effect to balance binding affinity, conformational adaptability.

## Conclusion

In conclusion, using a molecular hybridization approach, two novel series of indole-based sulfonate derivatives were effectively designed and synthesized as possible inhibitors of cyclin-dependent kinase 2 (CDK-2). Indole was combined with alkanesulfonate and aspirin analogs to generate compounds that significantly inhibited the growth of the NCI-60 cancer cell panel. According to molecular docking and ADME predictions, compound **8a** showed the strongest cytotoxic effect and excellent CDK-2 inhibitory activity and this was supported by promising binding mode and interactions in the docking study as well as promising drug like properties in ADMET study. These results suggest that further structural optimization of the indole-sulfonate scaffold may lead to the development of new and potent anticancer drugs that specifically target CDK-2.

## Experimental

### Chemistry

Melting points were determined using a Stuart SMP30 melting point apparatus and are uncorrected. Nuclear magnetic resonance (NMR) spectra were acquired on a JEOL AS 500 spectrometer operating at 500 MHz for ^1^H and 125 MHz for ^13^C nuclei. Mass spectra were recorded on a Shimadzu GC-MS-QP 1000 EX (EI, 70 eV) spectrometer. Elemental microanalyses were recorded on a Vario El Elementar analyzer.

#### *General procedure for the synthesis of compounds*** 4a-c**, **6a-c** & **8a-c**

A mixture of 10 mmol of 1*H*-indole-3-carbohydrazide (**1**) and 10 mmol of *para*-substituted alkane sulfonyl aryl aldehydes **3**, **5** & **7** in 20 ml of acetic acid/ethanol (1:2) was reacted under reflux for 1 h. The resulting precipitate was then separated by filtration, dried, and recrystallized from ethanol to yield the novel compounds **4a-c**, **6a-c** & **8a-c**.

##### (E)-4-((2-(1H-indole-3-carbonyl)hydrazono)methyl)phenyl methanesulfonate (**4a**)

Yield: 87%; m.p. 198–200 °C; Color: brown; ^1^H-NMR (500 MHz, DMSO-d_6_) δ (ppm): 3.43 (s, 3 H, *CH*_*3*_), 7.15–7.22 (m, 2 H, arom. H), 7.43 (d, 2 H, *J* = 8.6 Hz, Ar-H), 7.49 (d, 1H, *J* = 7.6 Hz, Ar-H), 7.82 (d, 2 H, *J* = 8.6 Hz, Ar-H), 8.23–8.35 (m, 3 H, CH = N + arom. H), 11.46 (s, 1H, NH), 11.74 (s, 1H, NH); ^13^C NMR (125 MHz, (DMSO-d_6_) δ (ppm): 37.53, 38.89, 39.10, 39.31, 39.73, 39.94, 40.15, 111.95, 120.77, 121.16, 122.24, 122.68, 128.29, 133.95, 135.95, 149.61; Analysis % for C_17_H_15_N_3_O_4_S (357.38) Calcd. (Found) C, 57.13 (57.25), H, 4.61 (4.56), N, 11.76 (11.82).

##### (E)-4-((2-(1H-indole-3-carbonyl)hydrazono)methyl)phenyl ethanesulfonate (**4b**)

Yield: 88%; m.p. 232–234 °C; Color: buff; ^1^H-NMR (500 MHz, DMSO-d_6_) δ (ppm): 1.40 (t, 3 H, *J* = 7.3 Hz, CH_2_*CH*_*3*_), 3.56 (q, 2 H, *J* = 7.3 Hz, *CH*_*2*_CH_3_), 7.15–7.20 (m, 2 H, Ar-H), 7.41–7.50 (m, 4 H, Ar-H), 7.82 (d, 2 H, *J* = 8.6 Hz, Ar-H), 8.22 (br. s, 2 H, CH = N + Ar-H), 11.49 (s, 1H, NH), 11.76 (s, 1H, NH); ^13^C NMR (125 MHz, DMSO-d_6_) δ (ppm): 8.00, 44.75, 99.39, 111.90, 120.71, 121.11, 122.19, 122.52, 128.24, 133.81, 149.42; Analysis % for C_18_H_17_N_3_O_4_S (371.41) Calcd. (Found) C, 58.21 (57.98), H, 4.61 (4.54), N, 11.31 (11.38).

##### (E)-4-((2-(1 H-indole-3-carbonyl)hydrazono)methyl)phenyl propane-1-sulfonate (**4c**)

Yield: 92%; m.p. 196–197 °C; Color: buff; ^1^H-NMR (500 MHz, DMSO-*d*_*6*_) δ (ppm): 1.05 (t, 3 H, *J* = 7.5 Hz, CH_2_CH_2_*CH*_*3*_), 1.86 (q, 2 H, *J* = 7.5 Hz, CH_2_*CH*_*2*_CH_3_), 3.55 (m, 2 H, *CH*_*2*_CH_2_CH_3_), 7.15–7.22 (m, 2 H, Ar-H), 7.41 (d, 2 H, *J* = 8.7 Hz, Ar-H), 7.49 (d, 2 H, *J* = 7.9 Hz, Ar-H), 7.82 (d, 2 H, *J* = 8.8 Hz, Ar-H), 8.23 (br. s, 2 H, CH = N + Ar-H), 11.49 (s, 1H, NH), 11.76 (s, 1H, NH); ^13^C-NMR (125 MHz, DMSO-*d*_6_) δ (ppm): 12.38, 17.01, 51.45, 111.98, 120.80, 122.27, 122.65, 128.32, 133.86, 149.45; Analysis % for C_19_H_19_N_3_O_4_S (385.44) Calcd. (Found) C, 59.21 (59.34), H, 4.97 (5.04), N, 10.90 (10.76).

##### (E)-4-((2-(1H-indole-3-carbonyl)hydrazono)methyl)-2-methoxyphenyl methanesulfonate (**6a**)

Yield: 89%; m.p. 232–233 °C; Color: off white; ^1^H-NMR (500 MHz, DMSO-*d*_*6*_) δ (ppm): 2.46 (s, 3 H, SO_2_CH_3_), 2.99 (s, 3 H, OCH_3_), 6.24–6.29 (m, 3 H, Ar-H), 6.38–6.40 (m, 1H, Ar-H), 6.45 (d, 1H, *J* = 8.2 Hz, Ar-H), 6.57–6.59 (m, 2 H, Ar-H), 7.30–7.37 (m, 2 H, CH = N + Ar-H), 10.64 (s, 1H, NH), 10.89 (s, 1H, NH); ^13^C-NMR (125 MHz, DMSO-*d*_6_) δ (ppm): 38.40, 55.96, 110.40, 111.95, 119.84, 120.78, 121.15, 122.23, 124.21, 134.99, 135.96, 138.60, 151.72; Analysis % for C_18_H_17_N_3_O_5_S (387.41) Calcd. (Found) C, 55.81 (55.72), H, 4.42 (4.38), N, 10.85 (10.68).

##### (E)-4-((2-(1H-indole-3-carbonyl)hydrazono)methyl)-2-methoxyphenyl ethanesulfonate (**6b**)

Yield: 90%; m.p. 223–224 °C; Color: buff; ^1^H-NMR (500 MHz, DMSO-*d*_*6*_) δ (ppm): 1.41 (t, 3 H, *J* = 7.3 Hz, CH_2_*CH*_*3*_), 3.52 (q, 2 H, *J* = 7.3 Hz, *CH*_*2*_CH_3_), 3.93 (s, 3 H, *OCH*_*3*_), 7.14–7.22 (m, 2 H, arom. H), 7.30 (d, 1H, *J* = 8.3 Hz, arom. H), 7.37 (d, 1H, *J* = 8.3 Hz, arom. H), 7.49 (d, 2 H, *J* = 11.6 Hz, arom. H), 8.21 (d, 1H, *J* = 7.4 Hz, arom. H), 8.28 (br. s, 1H, CH = N), 11.46 (s, 1H, NH), 11.75 (s, 1H, NH); ^13^C NMR (125 MHz, DMSO-*d*_6_) δ (ppm): 8.04, 38.89, 39.10, 39.31, 39.52, 39.73, 39.94, 40.15, 45.67, 55.98, 111.93, 120.75, 122.21, 124.17, 134.86, 138.50, 151.67; Analysis % for C_19_H_19_N_3_O_5_S (401.44) Calcd. (Found) C, 56.85 (56.91), H, 4.77 (4.69), N, 10.47 (10.52).

##### (E)-4-((2-(1 H-indole-3-carbonyl)hydrazono)methyl)-2-methoxyphenyl propane-1-sulfonate (**6c**)

Yield: 79%; m.p. 160–161 °C; Color: off-white; ^1^H-NMR (500 MHz, DMSO-*d*_*6*_) δ (ppm): 1.50 (t, 3 H, *J* = 7.4 Hz, CH_2_CH_2_*CH*_*3*_), 1.89 (m, 2 H, CH_2_*CH*_*2*_CH_3_), 3.48 (t, 2 H, *J* = 7.6 Hz, *CH*_*2*_CH_2_CH_3_), 3.92 (s, 3 H, *OCH*_*3*_), 7.14–7.22 (m, 2 H, arom. H), 7.30 (d, 1H, *J* = 8.2 Hz, arom. H), 7.36 (d, 1H, *J* = 8.3 Hz, arom. H), 7.49 (d, 2 H, *J* = 11.6 Hz, arom. H), 8.21 (d, 1H, *J* = 7.3 Hz, arom. H), 8.28 (br. s, 1H, CH = N), 11.46 (s, 1H, NH), 11.75 (s, 1H, NH); ^13^C NMR (125 MHz, DMSO-*d*_6_) δ (ppm): 12.39, 17.03, 38.89, 39.10, 39.31, 39.73, 39.94, 40.15, 52.39, 55.99, 111.93, 120.74, 122.20, 124.22, 134.85, 138.49, 151.66; Analysis % for C_20_H_21_N_3_O_5_S (415.46) Calcd. (Found) C, 57.82 (57.90), H, 5.10 (5.04), N, 10.11 (10.23).

##### (E)-5-((2-(1H-indole-3-carbonyl)hydrazono)methyl)-2-methoxyphenyl methanesulfonate (**8a**)

Yield: 93%; m.p. 205–206 °C; Color: off-white; ^1^H-NMR (500 MHz, DMSO-*d*_*6*_) δ (ppm): 3.39 (s, 3 H, SO_2_CH_3_), 3.90 (s, 3 H, OCH_3_), 7.13–7.21 (m, 2 H, arom. H), 7.28 (d, 1H, *J* = 6.8 Hz, Ar-H), 7.46 (d, 1H, *J* = 9.4 Hz, arom. H), 7.64 (d, 2 H, *J* = 9.4 Hz, arom. H), 8.20 (d, 1H, *J* = 7.4 Hz, arom. H), 8.27 (br. s, 1H, CH = N), 11.35 (s, 1H, NH), 11.73 (s, 1H, NH); ^13^C-NMR (125 MHz, DMSO-*d*_*6*_) δ (ppm): 38.34, 38.89, 39.10, 39.31, 39.73, 39.94, 40.15, 56.22, 111.90, 113.77, 120.69, 121.31, 122.18, 127.14, 127.96, 138.12, 152.36.; Analysis % for C_18_H_17_N_3_O_5_S (387.41) Calcd. (Found) C, 55.81 (56.04), H, 4.42 (4.34), N, 10.85 (10.98).

##### (E)-5-((2-(1H-indole-3-carbonyl)hydrazono)methyl)-2-methoxyphenyl ethanesulfonate (**8b**)

Yield: 93%; m.p. 203–204 °C; Color: off-white; ^1^H-NMR (500 MHz, DMSO-*d*_*6*_) δ (ppm): 1.41 (t, 3 H, *J* = 7.3 Hz, CH_2_*CH*_*3*_), 3.52 (q, 2 H, *J* = 7.3 Hz, *CH*_*2*_CH_3_), 3.90 (s, 3 H, OCH_3_), 7.16–7.20 (m, 2 H, Ar-H), 7.27 (d, 1H, *J* = 8.6 Hz, Ar-H), 7.48 (d, 1H, *J* = 8.0 Hz, Ar-H), 7.63–7.69 (m, 2 H, Ar-H), 8.21 (br. s, 3 H, CH = N + Ar-H), 11.44 (s, 1H, NH), 11.78 (s, 1H, NH); ^13^C-NMR (125 MHz, DMSO-*d*_6_) δ (ppm): 8.03, 45.66, 56.21, 111.91, 113.69, 120.70, 120.79, 121.16, 122.19, 127.10, 127.93, 135.97, 138.06, 152.33; Analysis % for C_19_H_19_N_3_O_5_S (401.44) Calcd. (Found) C, 56.85 (56.96), H, 4.77 (4.83), N, 10.47 (10.64).

##### (E)-5-((2-(1 H-indole-3-carbonyl)hydrazono)methyl)-2-methoxyphenyl propane-1-sulfonate (**8c**)

Yield: 91%; m.p. 211–212 °C; Color: buff crystals; ^1^H-NMR (500 MHz, DMSO-*d*_*6*_) δ (ppm): 1.01 (t, 3 H, *J* = 7.4 Hz, CH_2_CH_2_*CH*_*3*_), 1.86 (m, 2 H, CH_2_*CH*_*2*_CH_3_), 3.46 (t, 2 H, *J* = 7.6 Hz, *CH*_*2*_CH_2_CH_3_), 3.86 (s, 3 H, OCH_3_), 7.13–7.19 (m, 2 H, Ar-H), 7.22 (d, 1H, *J* = 8.6 Hz, Ar-H), 7.46 (d, 1H, *J* = 7.8 Hz, Ar-H), 7.59–7.64 (m, 2 H, Ar-H), 8.21 (br. s, 3 H, CH = N + Ar-H), 11.42 (s, 1H, NH), 11.76 (s, 1H, NH); ^13^C-NMR (125 MHz, DMSO-*d*_6_) δ (ppm): 12.38, 17.01, 51.45, 111.98, 120.80, 122.27, 122.65, 128.32, 133.86, 149.45; Analysis % for C_20_H_21_N_3_O_5_S (415.46) Calcd. (Found) C, 57.82 (57.78), H, 5.10 (5.18), N, 10.11 (9.96).

#### General procedure for the synthesis of compounds **10a**,** 12** & **14a**,** b**

A mixture of 10 mmol of 1*H*-indole-3-carbohydrazide (**1**) and 10 mmol of the formyl acetoxy benzoates **9**,**11**, & **13** in 20 ml of acetic acid/ethanol (1:2) was reacted under reflux for 1 h. The resulting precipitate was then separated by filtration, dried, and recrystallized from ethanol to yield the novel compounds **10a**,** 12** & **14a**,** b**.

##### (E)-4-((2-(1H-indole-3-carbonyl)hydrazono)methyl)phenyl 2-acetoxybenzoate (**10a**)

Yield: 89%; m.p. 207–209 °C; Color: buff; ^1^H-NMR (500 MHz, DMSO-*d*_*6*_) δ (ppm): 3.44 (s, 3 H, COCH_3_), 7.09 (d, 1H, *J* = 8.8 Hz, Ar-H), 7.15–7.22 (m, 3 H, Ar-H), 7.40 (d, 2 H, *J* = 8.4 Hz, Ar-H), 7.49 (d, 2 H, *J* = 7.9 Hz, Ar-H), 7.60 (d, 1H, *J* = 9.2 Hz, Ar-H), 7.82 (d, 2 H, *J* = 8.3 Hz, Ar-H), 7.96 (s, 1H, Ar-H), 8.24 (br. s, 2 H, CH = N + Ar-H), 10.50 (s, 1H, NH), 11.79 (s, 1H, NH); ^13^C-NMR (125 MHz, DMSO-*d*_6_) δ (ppm): 20.59, 108.36, 111.93, 115.29, 115.69, 119.67, 120.73, 121.18, 122.21, 122.38, 122.54, 122.77, 126.38, 127.84, 128.79, 129.86, 132.89, 135.25, 135.98, 150.89, 158.48, 164.82; Analysis % for C_25_H_19_N_3_O_5_ (441.44) Calcd. (Found) C, 68.02 (67.87), H, 4.34 (4.26), N, 9.52 (9.43).

##### (E)-4-((2-(1H-indole-3-carbonyl)hydrazono)methyl)-2-methoxyphenyl 2-acetoxybenzoate (**12**)

Yield: 87%; m.p. 231–233 °C; Color: off-white; ^1^H-NMR (500 MHz, DMSO-*d*_*6*_) δ (ppm): 2.75 (s, 3 H, COCH_3_), 4.11 (s, 3 H, OCH_3_), 7.27–7.33 (m, 1H, Ar-H), 7.41–7.61 (m, 3 H, Ar-H), 7.68 (d, 1H, *J* = 7.8 Hz, Ar-H), 7.77–7.79 (m, 2 H, Ar-H), 7.85 (t, 1H, *J* = 7.9 Hz, Ar-H), 7.96 (d, 1H, *J* = 5.05 Hz, Ar-H), 8.05 (t, 1H, *J* = 7.9 Hz, Ar-H), 8.26 (d, 1H, *J* = 7.85 Hz, Ar-H), 8.42 (d, 1H, *J* = 7.5 Hz, Ar-H), 9.03 (s, 1H, CH = N); ^13^C-NMR (125 MHz, DMSO-*d*_6_) δ (ppm): δ 21.17, 56.54, 111.97, 120.22, 122.36, 122.57, 123.97, 124.92, 127.16, 131.49, 132.32, 133.50, 135.79, 142.04, 151.06, 161.45, 162.41, 169.77; Analysis % for C_26_H_21_N_3_O_6_ (471.47) Calcd. (Found) C, 66.24 (65.98), H, 4.49 (4.46), N, 8.91 (8.84).

##### (E)-5-((2-(1H-indole-3-carbonyl)hydrazono)methyl)-2-methoxyphenyl 2-acetoxybenzoate (**14a**)

Yield: 90%; m.p. 214–216 °C; Color: pale yellow; ^1^H-NMR (500 MHz, DMSO-*d*_*6*_) δ (ppm): 3.42 (s, 3 H, COCH_3_), 3.84 (s, 3 H, OCH_3_), 7.04–7.08 (m, 2 H, Ar-H), 7.14–7.21 (m, 3 H, Ar-H), 7.26 (d, 1H, *J* = 8.6 Hz, Ar-H), 7.31 (d, 1H, *J* = 8.5 Hz, Ar-H), 7.48 (d, 1H, *J* = 7.9 Hz, Ar-H), 7.59–7.67 (m, 2 H, Ar-H), 7.81 (d, 1H, *J* = 9.3 Hz, Ar-H), 8.03 (d, 1H, *J* = 7.7 Hz, Ar-H), 8.24 (br. s, 1H, CH = N), 10.29 (s, 1H, NH), 11.76 (s, 1H, NH); ^13^C NMR (125 MHz, DMSO-*d*_6_) δ (ppm): 18.48, 56.01, 111.87, 112.65, 113.06, 117.63, 119.55, 120.66, 121.16, 122.14, 126.34, 126.86, 127.92, 130. 83, 136.15, 139.16, 151.94, 153.35, 160.26, 166.16; Analysis % for C_26_H_21_N_3_O_6_ (471.47) Calcd. (Found) C, 66.24 (66.32), H, 4.49 (4.58), N, 8.91 (9.04).

##### (E)-5-((2-(1H-indole-3-carbonyl)hydrazono)methyl)-2-methoxyphenyl 2-acetoxy-4-chlorobenzoate (**14b**)

Yield: 83%; m.p. 216–217 °C; Color: off-white; ^1^H-NMR (500 MHz, DMSO-*d*_*6*_) δ (ppm): 3.36 (s, 3 H, COCH_3_), 3.84 (s, 3 H, SO_2_CH_3_), 7.09–7.20 (m, 2 H, Ar-H), 7.26–7.28 (m, 2 H, Ar-H), 7.31–7.32 (d, 1H, Ar-H), 7.47 (d, 1H, *J* = 10 Hz, Ar-H), 7.62 (d, 1H, *J* = 10 Hz, Ar-H), 7.66 (s, 1H, Ar-H), 7.80–8.21 (m, 2 H, Ar-H), 8.66 (s, 1H, Ar-H), 10.45 (br. s, 1H, CH = N), 11.40 (br.s, 1H, NH), 11.73 (s, 1H, NH); ^13^C NMR (125 MHz, DMSO-*d*_6_) δ (ppm): 39.52, 56.12, 99.40, 111.84, 113.06, 114.84, 119.76, 120.61, 121.12, 122.10, 122.74, 126.36, 127.88, 129.85, 135.34, 139.12, 151.85, 158.51; Analysis % for C_26_H_20_ClN_3_O_6_ (505.91) Calcd. (Found) C, 61.73 (61.85), H, 3.98 (3.90), N, 8.31 (8.29).

## Supplementary Information

Below is the link to the electronic supplementary material.

Supplementary Material 1.

## Data Availability

The datasets generated during and/or analyzed during the current study are available from the corresponding author on reasonable request.

## References

[1] Malumbres M. Cyclin-dependent kinases. Genome Biol. 2014;15:122. 10.1186/gb4184. 25180339 10.1186/gb4184 PMC4097832

[2] Roskoski R. Cyclin-dependent protein kinase inhibitors, including palbociclib as anticancer drugs. Pharmacol Res. 2019;139:471–88. 10.1016/j.phrs.2018.11.035. 26995305 10.1016/j.phrs.2016.03.012

[3] Lim S, Kaldis P. Cdks, cyclins and CKIs: roles beyond cell cycle regulation. Development. 2013;140(15):3079–93. 10.1242/dev.091744. 23861057 10.1242/dev.091744

[4] Hydbring P, Malumbres M, Sicinski P. Non-canonical functions of cell cycle cyclins and cyclin-dependent kinases. Nat Rev Mol Cell Biol. 2016;17:280–92. 10.1038/nrm.2016.27. 27033256 10.1038/nrm.2016.27 PMC4841706

[5] De Bondt HL, Rosenblatt J, Jancarik J, Jones HD, Morgan DO, Kim SH. Crystal structure of cyclin-dependent kinase 2. Nature. 1993;363:595–602. 10.1038/363595a0. 8510751 10.1038/363595a0

[6] Tadesse S, Caldon EC, Tilley W, Wang S. Cyclin-dependent kinase 2 inhibitors in cancer therapy: an update. J Med Chem. 2019;62(9):4233–51. 10.1021/acs.jmedchem.8b01729. 30543440 10.1021/acs.jmedchem.8b01469

[7] House I, Valore-Caplan M, Maris E, Falchook GS. Cyclin Dependent Kinase 2 (CDK2) Inhibitors in Oncology Clinical Trials: A Review. J Immunother Precis Oncol. 2025;8(1):47–54. 10.36401/JIPO-24-22. 39811424 10.36401/JIPO-24-22 PMC11728386

[8] Jansa J, Jorda R, Škerlová J, Pachl P, Peřina M, Řezníčková E, Heger T, Gucký T, Řezáčová P, Lyčka A, Kryštof V. Imidazo[1,2-c]pyrimidin-5(6H)-one inhibitors of CDK2: Synthesis, kinase inhibition and co-crystal structure. Eur J Med Chem. 2021;216:113309. 33711765 10.1016/j.ejmech.2021.113309

[9] Chen W, Zhuang X, Chen Y, Shen L, Yang H, Wang M, Pan G, Tan J, Pan X, Feng S, Yuan K, Zhang XY, Yang P. Discovery of potent and selective CDK2 inhibitors with high safety and favorable bioavailability for the treatment of cancer. Eur J Med Chem. 2025;290:117503. 10.1016/j.ejmech.2025.117503. 40107208 10.1016/j.ejmech.2025.117503

[10] Dietrich C, Trub A, Ahn A, Taylor M, Ambani K, Chan KT, Lu KH, Mahendra CA, Blyth C, Coulson R, Ramm S, Watt AC, Matsa SK, Bisi J, Strum J, Roberts P, Goel S. INX-315, a Selective CDK2 Inhibitor, Induces Cell Cycle Arrest and Senescence in Solid Tumors. Cancer Discov. 2024;14(3):446–67. 10.1158/2159-8290.CD-23-0954. 38047585 10.1158/2159-8290.CD-23-0954 PMC10905675

[11] Shen C, Qiu M, Huser N et al. PF-07104091, a first-in-class CDK2-selective inhibitor for the treatment of HR+/HER2- breast cancer and CCNE1high ovarian cancer. Cancer Research 2024;84(6_Supplement):Abstract 5709. 10.1158/1538-7445.AM2024-5709

[12] National Cancer Institute. Definition of CDK2 Inhibitor PF-07104091. NCI Drug Dictionary; 2025. Cancer.gov.

[13] National Cancer Institute. Definition of CDK2 Inhibitor BLU-222. NCI Drug Dictionary; 2025. Cancer.gov.

[14] National Cancer Institute. Definition of CDK2 Inhibitor INCB123667. NCI Drug Dictionary; 2025.

[15] House NC, Chen MM, Khazaei S, Brown V, Ramsden P, Peng DH, Moore S, Yuan L, Wu R, Wang F, Luo L, Feng N, Bristow CA, Yap TA, Keyomarsi K, Marszalek JR, Ribich S, Rinne ML, Muthuswamy LB, Faia KL. CDK2 inhibition enhances CDK4/6 inhibitor antitumor activity in comprehensive breast cancer PDX model screen. NPJ Breast Cancer. 2025;11(1):135. 10.1038/s41523-025-00851-7. 41339342 10.1038/s41523-025-00851-7 PMC12675586

[16] Tadesse S, Yu M, Kumarasiri M, Le BT, Wang S. Targeting CDK2 for cancer therapy: discovery of selective CDK2 inhibitors from a library of 2-anilino-4-(thiazol-5-yl)pyrimidines. J Med Chem. 2015;58(10):4713–24. 10.1021/acs.jmedchem.5b00136.25961334

[17] Singh P, Choli A, Swain B, Angeli A, Sahoo SK, Yaddanapudi VM, Supuran CT, Arifuddin M. Design and development of novel series of indole-3-sulfonamide ureido derivatives as selective carbonic anhydrase II inhibitors. Arch Pharm (Weinheim). 2022;355(1):e2100333. 10.1002/ardp.202100333. Epub 2021 Oct 25. PMID: 34694638. 34694638 10.1002/ardp.202100333

[18] Agrawal K, Patel T, Patel R. Synthesis, biological activity of newly designed sulfonamide based indole derivative as anti-microbial agent. Futur J Pharm Sci. 2023;9:17. 10.1186/s43094-023-00466-4.

[19] Mo X, Rao DP, Kaur K, Hassan R, Abdel-Samea AS, Farhan SM, Bräse S, Hashem H. Indole Derivatives: A Versatile Scaffold in Modern Drug Discovery-An Updated Review on Their Multifaceted Therapeutic Applications (2020–2024). Molecules. 2024;29(19):4770. 10.3390/molecules29194770. 39407697 10.3390/molecules29194770 PMC11477627

[20] Al-Wahaibi LH, Mostafa YA, Abdelrahman MH, El-Bahrawy AH, Trembleau L, Youssif BGM. Synthesis and Biological Evaluation of Indole-2-Carboxamides with Potent Apoptotic Antiproliferative Activity as EGFR/CDK2 Dual Inhibitors. Pharmaceuticals. 2022;15(1006). 10.3390/ph15081006. 10.3390/ph15081006 PMC9414584 36015154

[21] Al-Sanea MM, Obaidullah AJ, Shaker ME, Chilingaryan G, Alanazi MM, Alsaif NA, Alkahtani HM, Alsubaie SA, Abdelgawad MA. A New CDK2 Inhibitor with 3-Hydrazonoindolin-2-One Scaffold Endowed with Anti-Breast Cancer Activity: Design, Synthesis, Biological Evaluation, and In Silico Insights. Molecules. 2021;26(2):412. 10.3390/molecules26020412. 33466812 10.3390/molecules26020412 PMC7830330

[22] Bramson HN, Corona J, Davis ST, Dickerson SH, Edelstein M, Frye SV, Gampe RT Jr, Harris PA, Hassell A, Holmes WD, Hunter RN, Lackey KE, Lovejoy B, Luzzio MJ, Montana V, Rocque WJ, Rusnak D, Shewchuk L, Veal JM, Walker DH, Kuyper LF. Oxindole-based inhibitors of cyclin-dependent kinase 2 (CDK2): design, synthesis, enzymatic activities, and X-ray crystallographic analysis. J Med Chem. 2001;44(25):4339-58. 10.1021/jm010117d 10.1021/jm010117d 11728181

[23] Mohamed FAM, Alakilli SYM, Azab E, Baawad EF, Shaaban FAM, Alrub EIA, Hendawy HA, Gomaa O, Bakr HAM, Abdelrahman AG, Trembleau MH, Mohammed L, A. F., Youssif BGM. Discovery of new 5-substituted-indole-2-carboxamides as dual epidermal growth factor receptor (EGFR)/cyclin-dependent kinase-2 (CDK2) inhibitors with potent antiproliferative action. RSC Med Chem. 2023;14(4):734–44. 10.1039/d3md00038a. 37122549 10.1039/d3md00038a PMC10131667

[24] (a) https://go.drugbank.com/drugs/DB00549?utm; (b) https://go.drugbank.com/drugs/DB08659; (c) https://go.drugbank.com/drugs/DB08039

[25] (a) https://go.drugbank.com/drugs/DB00918; (b) https://go.drugbank.com/drugs/DB00216; (c) https://go.drugbank.com/drugs/DB00669 ; (d) https://go.drugbank.com/drugs/DB06370

[26] Li X, Huang P, Cui JJ, Zhang J, Tang C. Novel pyrrolyllactone and pyrrolyllactam indolinones as potent cyclin-dependent kinase 2 inhibitors. Bioorg Med Chem Lett. 2003;13:1939–42. 10.1016/S0960-894X(03)00312-3. 12749903 10.1016/s0960-894x(03)00312-3

[27] Li H, Liu L, Zhuang J, Liu C, Zhou C, Yang J, Gao C, Liu G, Sun C. Deciphering the mechanism of Indirubin and its derivatives in the inhibition of Imatinib resistance using a drug target prediction-gene microarray analysis-protein network construction strategy. BMC Complement Altern Med. 2019;19:1–13. 10.1186/s12906-019-2471-2. 30909944 10.1186/s12906-019-2471-2 PMC6434895

[28] Mohamed-Ezzat RA, Elgemeie GH. Novel synthesis of the first new class of triazine sulfonamide thioglycosides and the evaluation of their anti-tumor and anti-viral activities against human coronavirus. Nucleosides Nucleotides Nucleic Acids. 2024;43(12):1511–28. 38753464 10.1080/15257770.2024.2341406

[29] Mohamed-Ezzat RA, Elgemeie GH. Novel synthesis of new triazine sulfonamides with antitumor, anti-microbial and anti-SARS-CoV-2 activities. BMC Chem 2024, 18, 58. 38532431 10.1186/s13065-024-01164-9 PMC10967038

[30] Mohamed-Ezzat RA, Elgemeie GH, Jones PG. An unexpected tautomer: synthesis and crystal structure of N-[6-amino-4-(methylsulfanyl)-1, 2-dihydro-1, 3, 5-triazin-2-ylidene]benzenesulfonamide. Acta Crystallogr Sect E: Crystallographic Commun. 2024;80(2):120–4. 10.1107/S2056989023011076 PMC10848973 38333139

[31] Mohamed-Ezzat RA, Kariuki BM, Azzam RA. Synthesis and crystal structure of N-(5-acetyl-4-methylpyrimidin-2-yl) benzenesulfonamide. Acta Crystallogr Sect E: Crystallographic Commun. 2023;79(4):331–4. 10.1107/S2056989023001871 PMC10088322 37057009

[32] Mohamed-Ezzat RA, Kariuki BM, Azzam RA. Morpholin-4-ium [5-cyano-6-(4-methylphenyl)-4-(morpholin-4-yl) pyrimidin-2-yl](phenylsulfonyl) amide. IUCrData. 2022;7(11):x221033.

[33] Mohamed-Ezzat RA, Kariuki B, Elgemeie G. Synthesis and crystal structure of N-phenyl-2-(phenylsulfanyl) acetamide. Acta Crystallogr Sect E: Crystallographic Commun. 2024;80(4):392–5. 10.1107/S2056989024002573 PMC10993607 38584738

[34] Mohamed-Ezzat RA, Kariuki BM, Azzam RA. Discovery of promising sulfadiazine derivatives with anti-proliferative activity against tumor cell lines. J Heterocycl Chem. 2024;61(12):1980–98.

[35] Mohamed-Ezzat RA, Elgemeie GH, Jones PG. Crystal structure of 4-chloro-*N*-{5-(4-methoxyphenyl)-4-[(4-methoxyphenyl)amino]-6-sulfanylidene-1,2,5,6-tetrahydro-1,3,5-triazin-2-ylidene}benzenesulfonamide dimethyl sulfoxide disolvate. Acta Crystallogr Sect E: Crystallographic Commun. 2025;81(12):1178–81. 10.1107/S2056989025010400 PMC12810283 41551399

[36] Fatahala SS, Sayed AI, Mahgoub S, Taha H, El-Sayed MK, El-Shehry MF, Awad SM, Abd El-Hameed RH. Synthesis of Novel 2-Thiouracil-5-Sulfonamide Derivatives as Potent Inducers of Cell Cycle Arrest and CDK2A Inhibition Supported by Molecular Docking. Int J Mol Sci. 2021;22(21):11957. 10.3390/ijms222111957. PMID: 34769385; PMCID: PMC8584424. 34769385 10.3390/ijms222111957 PMC8584424

[37] Pingaew R, Mandi P, Prachayasittikul V, Thongnum A, Prachayasittikul S, Ruchirawat S, Prachayasittikul V. Investigations on Anticancer and Antimalarial Activities of Indole-Sulfonamide Derivatives and *In Silico* Studies. ACS Omega. 2021;6(47):31854–68. 10.1021/acsomega.1c04552. 34870008 10.1021/acsomega.1c04552 PMC8638007

[38] Chinchilli KK, Singh P, Swain B, Goud NS, Sigalapalli DK, Choli A, Angeli A, Nanduri S, Yaddanapudi VM, Supuran CT, Arifuddin M. Development of Novel Indole-3-sulfonamide-heteroaryl Hybrids as Carbonic Anhydrase Inhibitors: Design, Synthesis and *in-vitro* Screening. Anticancer Agents Med Chem. 2023;23(11):1225–1233. 10.2174/1871520623666230227092821 10.2174/1871520623666230227092821 36847230

[39] Demir-Yazıcı K, Bua S, Akgüneş NM, Akdemir A, Supuran CT, Güzel-Akdemir Ö. Indole-Based Hydrazones Containing A Sulfonamide Moiety as Selective Inhibitors of Tumor-Associated Human Carbonic Anhydrase Isoforms IX and XII. Int J Mol Sci. 2019;20:2354. 10.3390/ijms20092354. 31083645 10.3390/ijms20092354 PMC6539891

[40] Wasi Ullah F, Rahim S, Hayat H, Ullah M, Taha S, Khan A, Khaliq S, Bibi O, Gohar N, Iqbal. Syed Adnan Ali Shah, Khalid Mohammed Khan, Synthesis of Indole Based Sulfonamide Derivatives as potent inhibitors of α-glucosidase and α-amylase in management of type-II diabetes. Chem Data Collections. 2024;50:101122. 10.1016/j.cdc.2024.101122.

[41] Ibrahim M, Taha M, Almandil NB, et al. Synthesis, characterization and electrochemical properties of some biologically important indole-based-sulfonamide derivatives. BMC Chem. 2020;14:38. 10.1186/s13065-020-00691-5. 32514499 10.1186/s13065-020-00691-5 PMC7254745

[42] Kassem AF, Mounier MM, Abdelglil MI, Abdelwahed S, El-Rashedy AA, Saleh A, Saleh MGA, Srour AM. Dual Anticancer and COX-2 Inhibitory Activity of Tailored Paracetamol-Alkanesulfonate Conjugates: A Promising Therapeutic Approach. Arch Pharm (Weinheim). 2025;358(10):e70125. 10.1002/ardp.70125. 41103087 10.1002/ardp.70125

[43] Mohamed-Ezzat RA, Srour AM. Design and Synthesis of Aspirin-chalcone Mimic Conjugates as Potential Anticancer Agents. Anticancer Agents Med Chem. 2024;24(7):544–57. 38204260 10.2174/0118715206280025231213065519

[44] Mohamed-Ezzat RA, Kariuki BM, Srour AM. Synthesis, crystal structure and *in vitro* anti-proliferative activity of 2-[(4-acetylphenyl)carbamoyl]phenyl acetate. Acta Cryst. 2023;E79:999–1002. 10.1107/S2056989023008526 PMC10626967 37936857

[45] Altwaijry NA, Omar MA, Mohamed HS, Mounier MM, Afifi AH, Srour AM. Design, synthesis, molecular docking and anticancer activity of benzothiazolecarbohydrazide-sulfonate conjugates: insights into ROS-induced DNA damage and tubulin polymerization inhibition. RSC Adv. 2025;15(8):5895–905. 10.1039/d4ra07810a. 39990814 10.1039/d4ra07810a PMC11843914

[46] Kassem AF, Ragab SS, Omar MA, Altwaijry NA, Abdelraof M, Temirak A, Saleh A, Srour AM. New Quinazolone-Sulfonate Conjugates with Acetohydrazide Linker as Potential Antimicrobial Agents: Design, Synthesis and Molecular Docking Simulations. RSC Adv. 2025;15:1033–48. 39807202 10.1039/d4ra07563c PMC11726445

[47] Mohamed-Ezzat RA, Kariuki BM, Elgemeie GH. Unexpected products of the reaction of cyanoacetylhydrazones of aryl/heteryl ketones with hydrazine: a new route to aryl/heteryl hydrazones, x-ray structure, and in vitro anti-proliferative activity against nci 60-cell line panel. Egypt J Chem. 2023;66(13):225–39.

[48] Elgemeie GH, Salah AM, Mohamed RA, Jones PG. Crystal structure of (E)-2-amino-4-methylsulfanyl-6-oxo-1-{[(thiophen-2-yl)methylidene]amino}-1,6-dihydropyrimidine-5-carbonitrile. Acta Crystallogr Sect E: Crystallogr Commun. 2015;71:1319–21. 26594500 10.1107/S205698901501885X PMC4644996

[49] Mohamed-Ezzat RA, Elgemeie GH. Discovery and Synthesis of Novel Bio-Isostere of Purine Analogues Inhibiting SARS-CoV-2. Egypt J Chem. 2023;66(13):167–85.

[50] Mohamed-Ezzat RA, Kariuki B, Al-Ashmawy AK, Srour AM. Synthesis and crystal structure of 5-{(*E*)-[(1*H*-indol-3-ylformamido)imino]methyl}-2-methoxyphenyl propane-1-sulfonate. Acta Crystallogr Sect E: Crystallographic Commun. 2025;81(4):310–3. 10.1107/S2056989025002087 PMC11974325 40201003

[51] Monks A, Scudiero D, Skehan P, Shoemaker R, Paull K, Vistica D, Hose C, Langley J, Cronise P, Vaigro-Wolff A, Gray-Goodrich M, Campbell H, Mayo J, Boyd M. Feasibility of a high-flux anticancer drug screen using a diverse panel of cultured human tumor cell lines. J Nat Cancer Inst. 1991;83(11):757–66. 2041050 10.1093/jnci/83.11.757

[52] Shoemaker RH. The NCI60 human tumor cell line anticancer drug screen. Nat Rev Cancer. 2006;6:813–23. 16990858 10.1038/nrc1951

[53] Boyd MR, Pauli KD. Some practical considerations and applications of the National-Cancer-Institute *in vitro* anticancer drug discovery screen. Drug Dev Res. 1995;34:91–109.

[54] DTP Developmental Therapeutics Program. https://dtp.cancer.gov/databases_tools/docs/compare/compare_methodology.htm (Accessed on 25 Sept 2022).

[55] Holbeck SL, Collins JM, Doroshow JH. Analysis of FDA-Approved Anti-Cancer Agents in the NCI60 Panel of Human Tumor Cell Lines. Mol Cancer Ther. 2010;9(5):1451–60. 20442306 10.1158/1535-7163.MCT-10-0106 PMC2868078

[56] Asghar U, Witkiewicz AK, Turner NC, Knudsen ES. The history and future of targeting cyclin-dependent kinases in cancer therapy. Nat Rev Drug Discov. 2015;14:130–46. 10.1038/nrd4504. 25633797 10.1038/nrd4504 PMC4480421

[57] Kassem AF, Sediek AA, Omran MM, Foda DS, Al-Ashmawy AAK. Design, synthesis and in vitro anti-proliferative evaluation of new pyridine-2,3-dihydrothiazole/thiazolidin-4-one hybrids as dual CDK2/GSK3β kinase inhibitors. RSC Adv. 2024;14:31607–23. 10.1039/d4ra06146b. 39376524 10.1039/d4ra06146b PMC11456921

[58] Abdelazeem NM, Aboulthana WM, Elshahid ZA, El-Hussieny M, Al-Ashmawy AAK. Synthesis and biological (in vitro and in silico) screening of the 4-aryl-fused pyranopyrazole derivatives as enzyme (α-amylase, α-glucosidase, acetylcholinesterase & proteinase) inhibitors with anti-oxidant and cytotoxic activities. J Mol Struct. 2024;1310:138224. 10.1016/j.molstruc.2024.138224.

[59] Daina A, Michielin O, Zoete V. SwissADME: A free web tool to evaluate pharmacokinetics, drug-likeness and medicinal chemistry friendliness of small molecules. Sci Rep. 2017;7:1–13. 10.1038/srep42717. 28256516 10.1038/srep42717 PMC5335600

[60] Banerjee P, Kemmler E, Dunkel M, Preissner R. ProTox 3.0: A webserver for the prediction of toxicity of chemicals. Nucleic Acids Res. 2024;52:W513–20. 10.1093/nar/gkae303. 38647086 10.1093/nar/gkae303 PMC11223834

[61] Trott O, Olson AJ. AutoDock Vina: Improving the speed and accuracy of docking with a new scoring function, efficient optimization, and multithreading. J Comput Chem. 2010;31:455–61. 10.1002/jcc.21334. 19499576 10.1002/jcc.21334 PMC3041641

[62] Al-Ashmawy AM, Abdelraof AAK. Mohamed, Saleh, Asmaa, Srour, Novel Benzimidazole ‐ Pyridine ‐ Phenylalkanesulfonate Hybrids: Design, Synthesis, Antimicrobial Screening, Lanosterol 14 α ‐ Demethylase Inhibition Properties and in Silico Studies. Drug Dev Res. 2025;86:e70122. 10.1002/ddr.70122. 40551522 10.1002/ddr.70122

[63] Wood DJ, Korolchuk S, Tatum NJ, Wang LZ, Endicott JA, Noble MEM, Martin MP. Differences in the Conformational Energy Landscape of CDK1 and CDK2 Suggest a Mechanism for Achieving Selective CDK Inhibition. Cell Chem Biol. 2019;26:121–e1305. 10.1016/j.chembiol.2018.10.015. 30472117 10.1016/j.chembiol.2018.10.015 PMC6344228

[64] Bettayeb K, Oumata N, Echalier A, Ferandin Y, Endicott JA, Galons H, Meijer L. CR8, a potent and selective, roscovitine-derived inhibitor of cyclin-dependent kinases. Oncogene. 2008;27:5797–807. 10.1038/onc.2008.191. 18574471 10.1038/onc.2008.191

[65] Krieger E, Joo K, Lee J, Lee J, Raman S, Thompson J, Tyka M, Baker D, Karplus K. Improving physical realism, stereochemistry, and side-chain accuracy in homology modeling: Four approaches that performed well in CASP8. Proteins. 2009;77:114–22. 10.1002/PROT.22570. 19768677 10.1002/prot.22570 PMC2922016

[66] O.A.E.F.M. Fathalla MAH, Ismail MM, Anwar KAM, Abouzid AAK, Ramadan. Novel 2-thiopyrimidine derivatives as CDK2 inhibitors: Molecular modeling, synthesis, and anti-tumor activity evaluation. Med Chem Res. 2013;22:659–73. 10.1007/s00044-012-0051-9.

[67] Xie F, Zhou L, Ge C, Song X, Yan H. Development of pyrazolo[3,4-d]pyrimidin-4-one scaffold as novel CDK2 inhibitors: Design, synthesis, and biological evaluation, Bioorganic Med. Chem Lett. 2022;70:128803. 10.1016/j.bmcl.2022.128803. 10.1016/j.bmcl.2022.128803 35598793

[68] Tadesse S, Anshabo AT, Portman N, Lim E, Tilley W, Caldon CE, Wang S. Targeting CDK2 in cancer: challenges and opportunities for therapy. Drug Discov Today. 2020;25:406–13. 10.1016/j.drudis.2019.12.001. 31839441 10.1016/j.drudis.2019.12.001

[69] Bai S, Wan S, Wu R, Li M, Tang S, Wang F, Chen L, Lv X. Novel acylhydrazone derivatives incorporating purine units: Design, synthesis, X-ray crystal structure, bioactivity evaluation, molecular docking and DFT calculations. J Mol Struct, (2026), p.144715. 10.1007/s11030-025-11190-x 40221614

