# Supplementary material for: Novel indole-based scaffolds: Design, synthesis, molecular modeling, and anti-proliferative evaluation
Source: BMC Chem. 2026 Apr 9;20(1):90. doi: 10.1186/s13065-026-01776-3 (PMC13094098; doi:10.1186/s13065-026-01776-3)
Supplement: Supplementary file 1 — Supplementary Material 1. [file 13065_2026_1776_MOESM1_ESM.docx]

**Novel Indole-Based Scaffolds: Design, Synthesis, Molecular Modeling, and Anti-Proliferative Evaluation**

**[Reham A. Mohamed-Ezzat](https://scripts.iucr.org/cgi-bin/citedin?search_on=name&author_name=Mohamed%2DEzzat%2C%20R%2EA%2E)^a*^**, [**Aisha A. K. Al-Ashmawy**](https://pubs.rsc.org/en/results?searchtext=Author%3AAisha%20A.%20K.%20Al-Ashmawy)**^b^**, [**Aladdin M. Srour**](https://scripts.iucr.org/cgi-bin/citedin?search_on=name&author_name=Srour%2C%20A%2EM%2E)[**^b^**](https://journals.iucr.org/e/issues/2023/11/00/dj2060/index.html#oidc)**^*^**

**^a^**Chemistry of Natural & Microbial Products Department, National Research Centre, Cairo, Egypt, and **^b^**Department of Therapeutic Chemistry, National Research Centre, Dokki, Cairo, 12622, Egypt

1. *US NCI methodology*

Assays for primary anticancer were carried out [1,2]. The cell culture was cultured for 48 hours after the compounds were administered at a single concentration. Endpoints were identified using the protein-binding dye sulforhodamine B (SRB). The efficacy of each chemical is indicated by the percentage of growth (GP%) of treated cells, which is then contrasted with the untreated control cells. Among the different cancer cell lines, the range of growth (%) showed the highest and lowest growth. Regarding sensitivity at a primary single high dose (10-5M) against cell lines. The panel of 60 human cancer cell lines received a single dosage (10 µM) of every compound that was evaluated. Lethality and in vitro growth inhibition were calculated as growth inhibition percentages. The in vitro growth inhibition and lethality were demonstrated as percentages of growth inhibition (G%) (values in the range of 0 to 100) and lethality (values ˂ 0). The percent growth inhibition (GI%) and lethality of each tested compound were shown and illustrated.

1. *Five-dose*

Compound **8a** fulfilled the threshold inhibition criterion in the single-dose screening and was eligible for assessment in the full-panel five-dose in vitro anticancer screening at 10-fold dilutions in the range of 0.01-100 µM. The biological potential of compound **8a** was determined using the response parameters (50% cell growth inhibition (growth inhibitory activity) (GI_50_), total cell growth inhibition (TGI) (cytostatic activity), and cytotoxic activity (50% cell death) (LC_50_)). While the TGI indicates complete growth inhibition, the LC_50_ indicates the dose at which 50% of cancer cells are destroyed. An indicative concentration at 50% growth inhibitory action is known as the GI_50_.

This assay utilized panels of human cancer cell lines, and dose-response curves were generated by calculating three parameters for each cell line: log concentration versus percent growth inhibition curves [3,4]. GI_50_ is the test drug’s concentration where 100 × (T-T0)/(C-T0) = 50. Following 48 hours of treatment with the test drug, T describes the optical density of the test well; T0 elucidates the optical density at time zero; ultimately, C is the control (nondrug) optical density.

The “50” is known as GI_50_PRCNT, a T/C-like parameter that can have values between +100 and −100. The TGI is the test drug concentration at which 100 × (T-T0)/(C-T0) = 0. The drug’s concentration at which 100 × (T-T0)/T0 = −50 is known as the LC_50_.


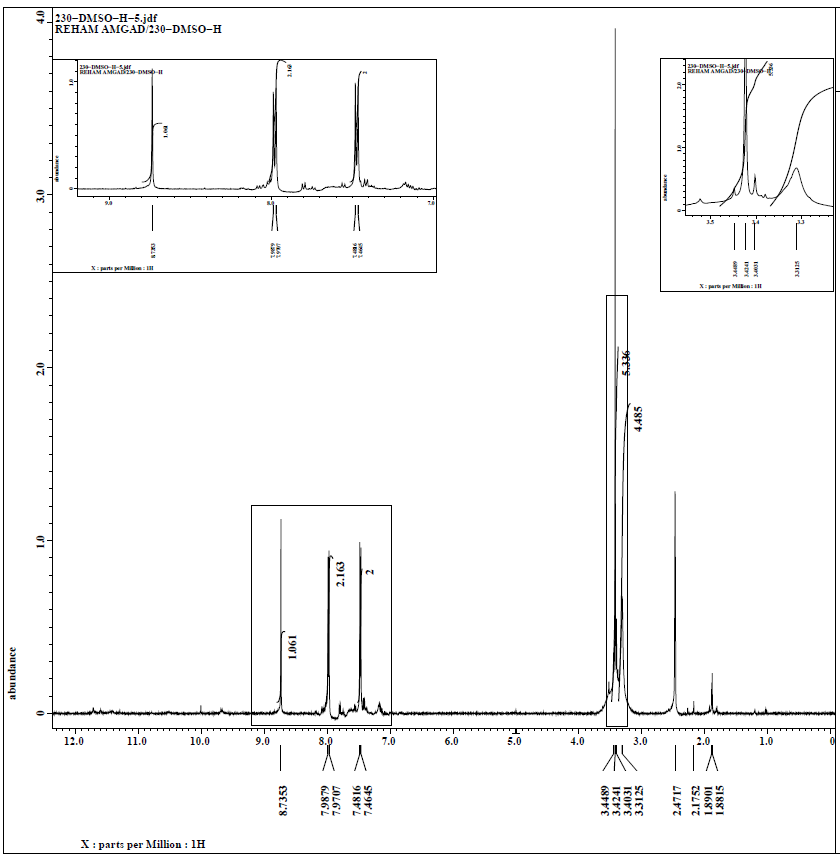


**Fig. S1.** ^1^H NMR spectrum of compound **4a**


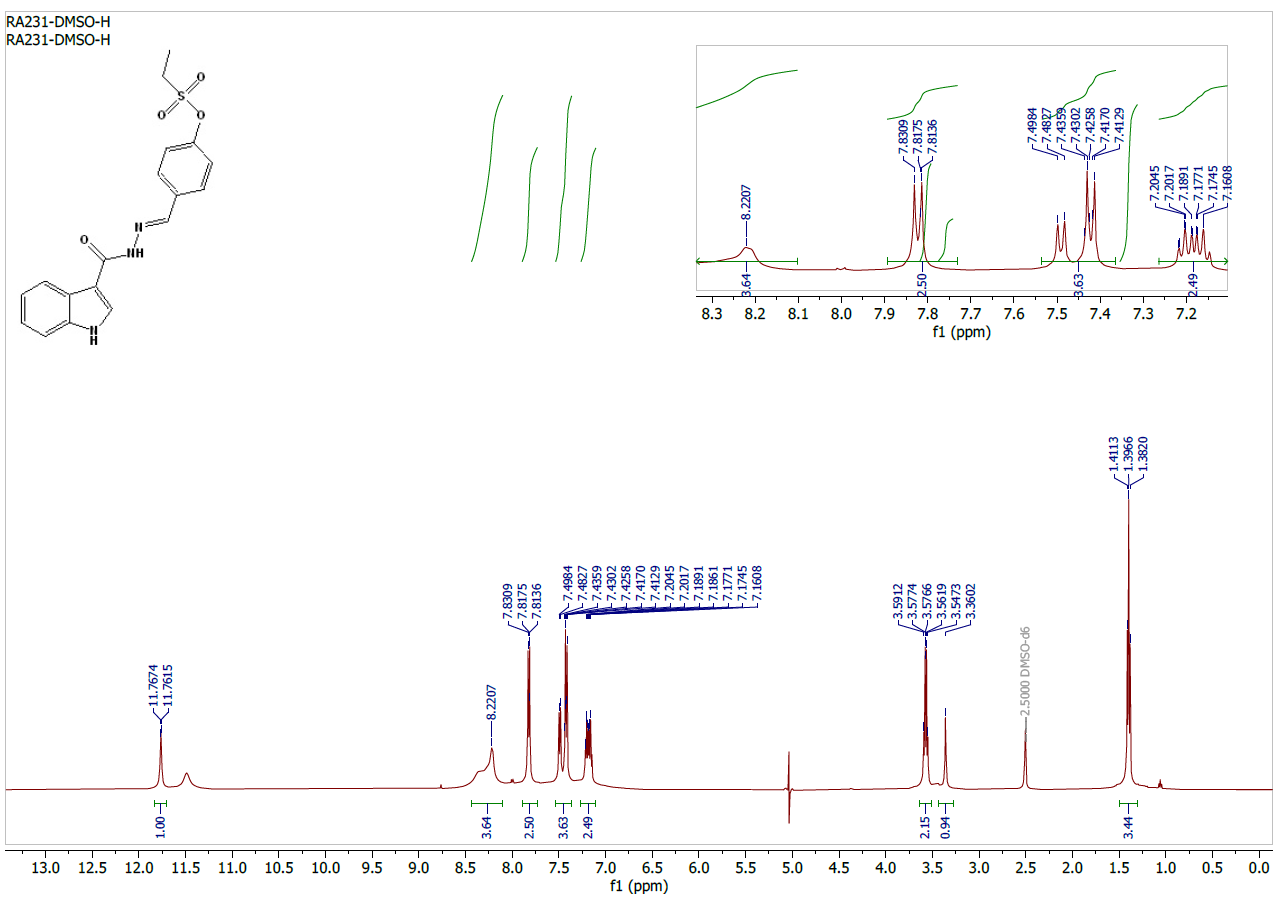


**Fig. S2.** ^1^H NMR spectrum of compound **4b**


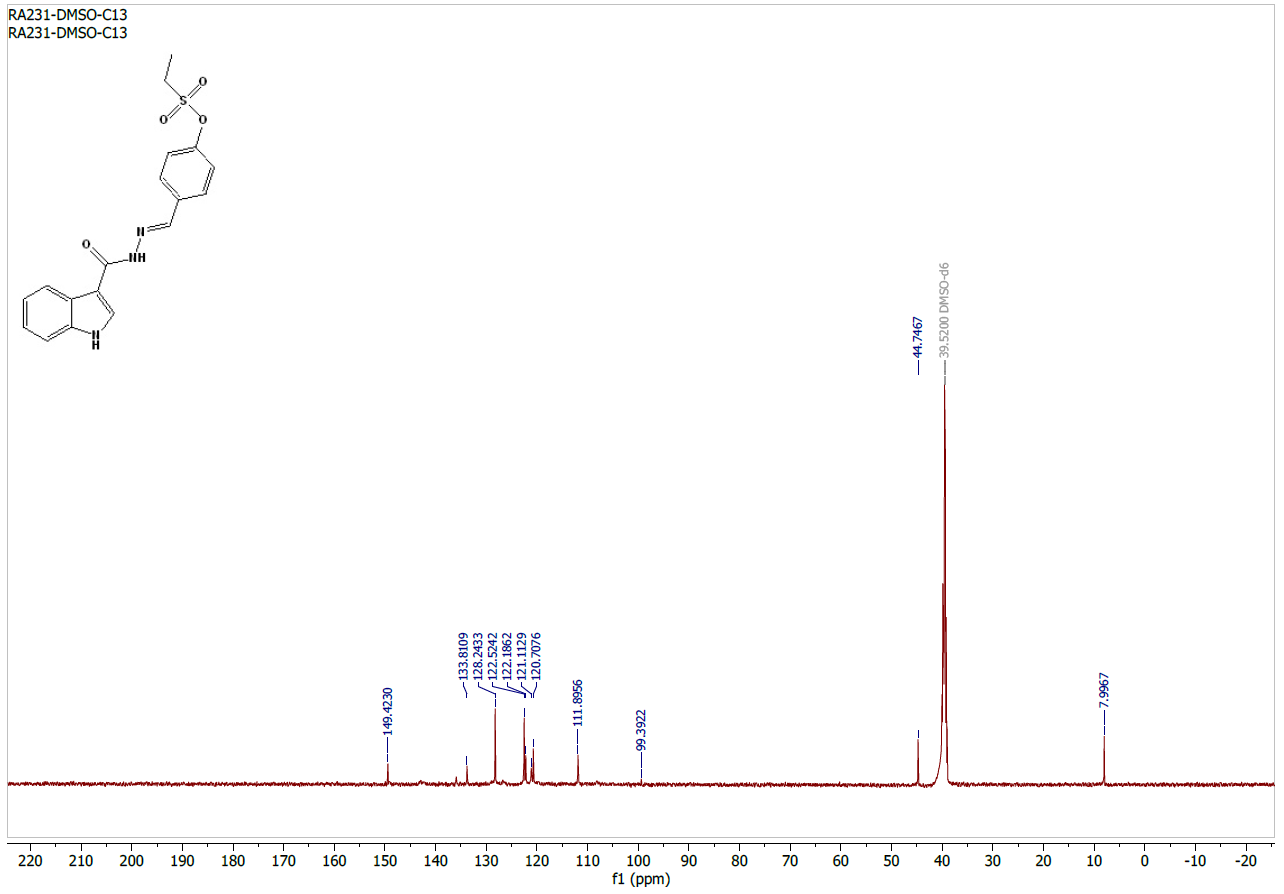


**Fig. S3.** ^13^C NMR spectrum of compound **4b**


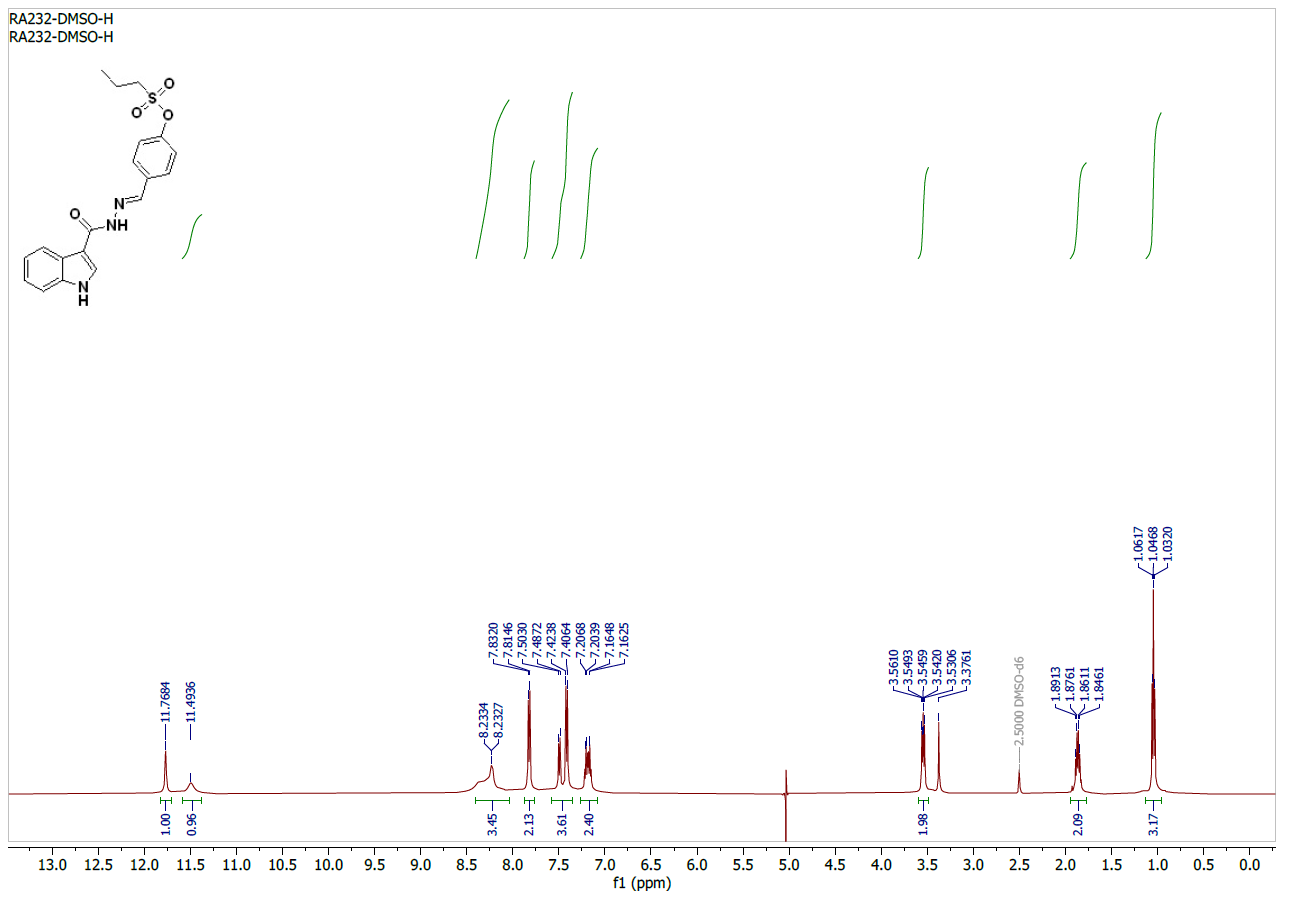


**Fig. S4.** ^1^H NMR spectrum of compound **4c**


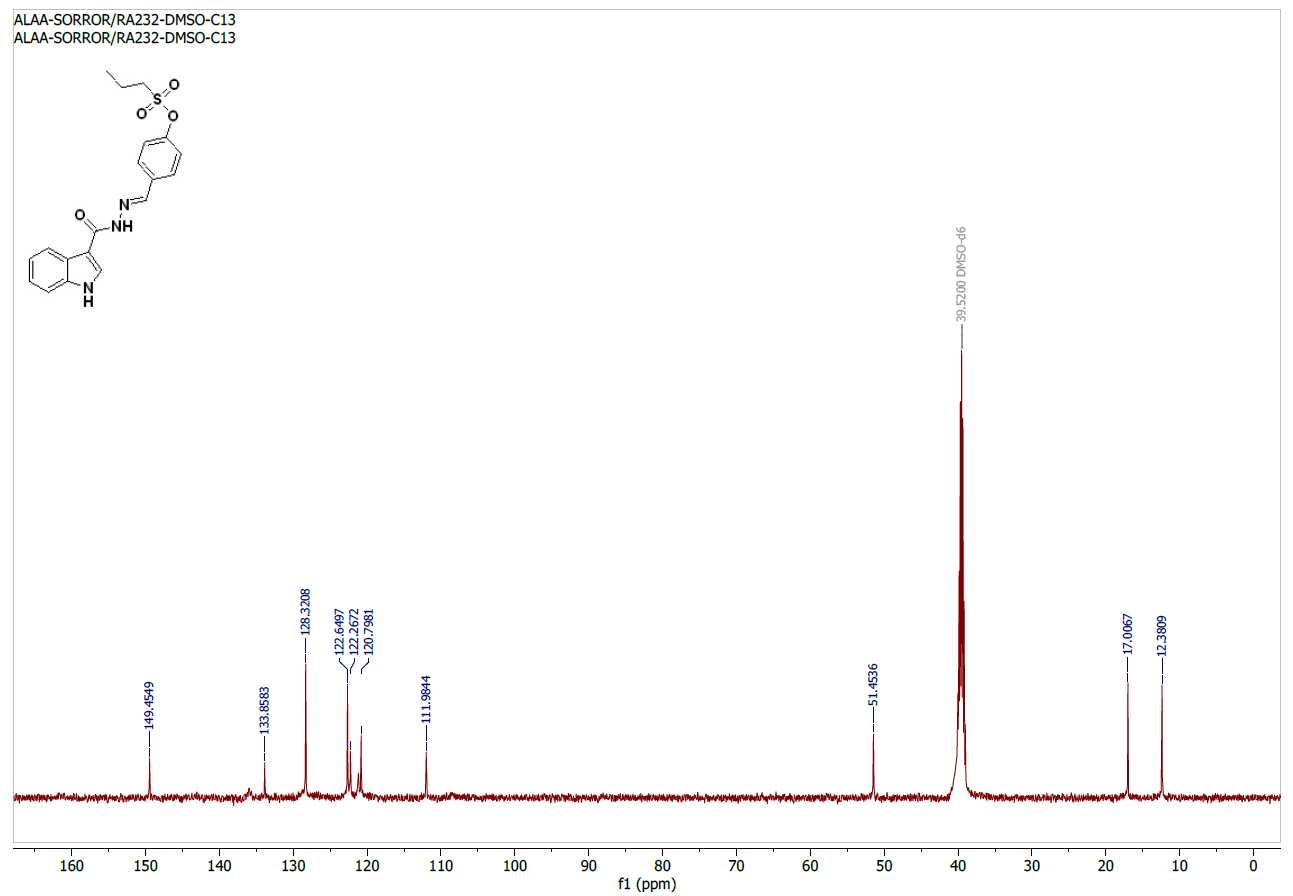


**Fig. S5.** ^13^C NMR spectrum of compound **4c**


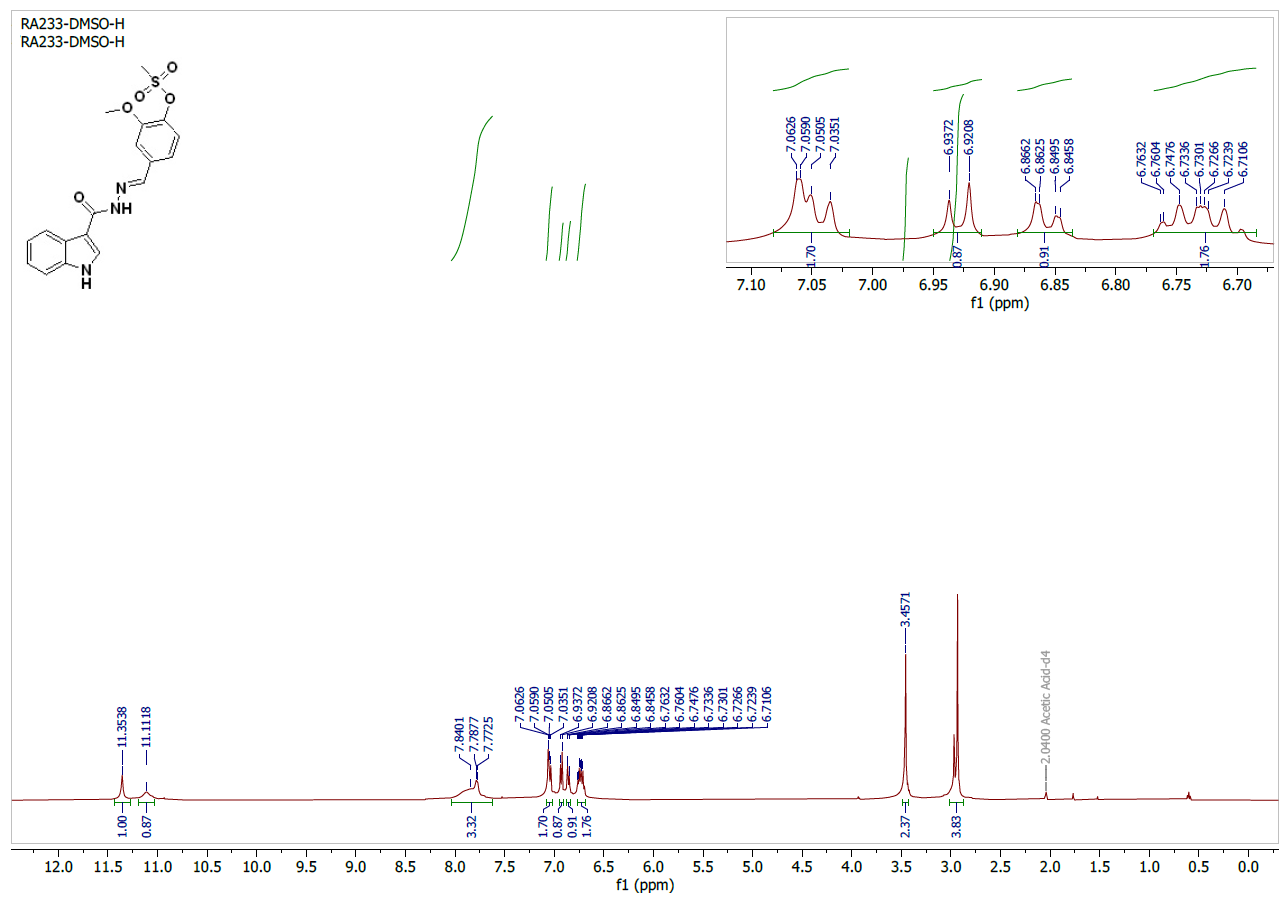


**Fig. S6.** ^1^H NMR spectrum of compound **6a**


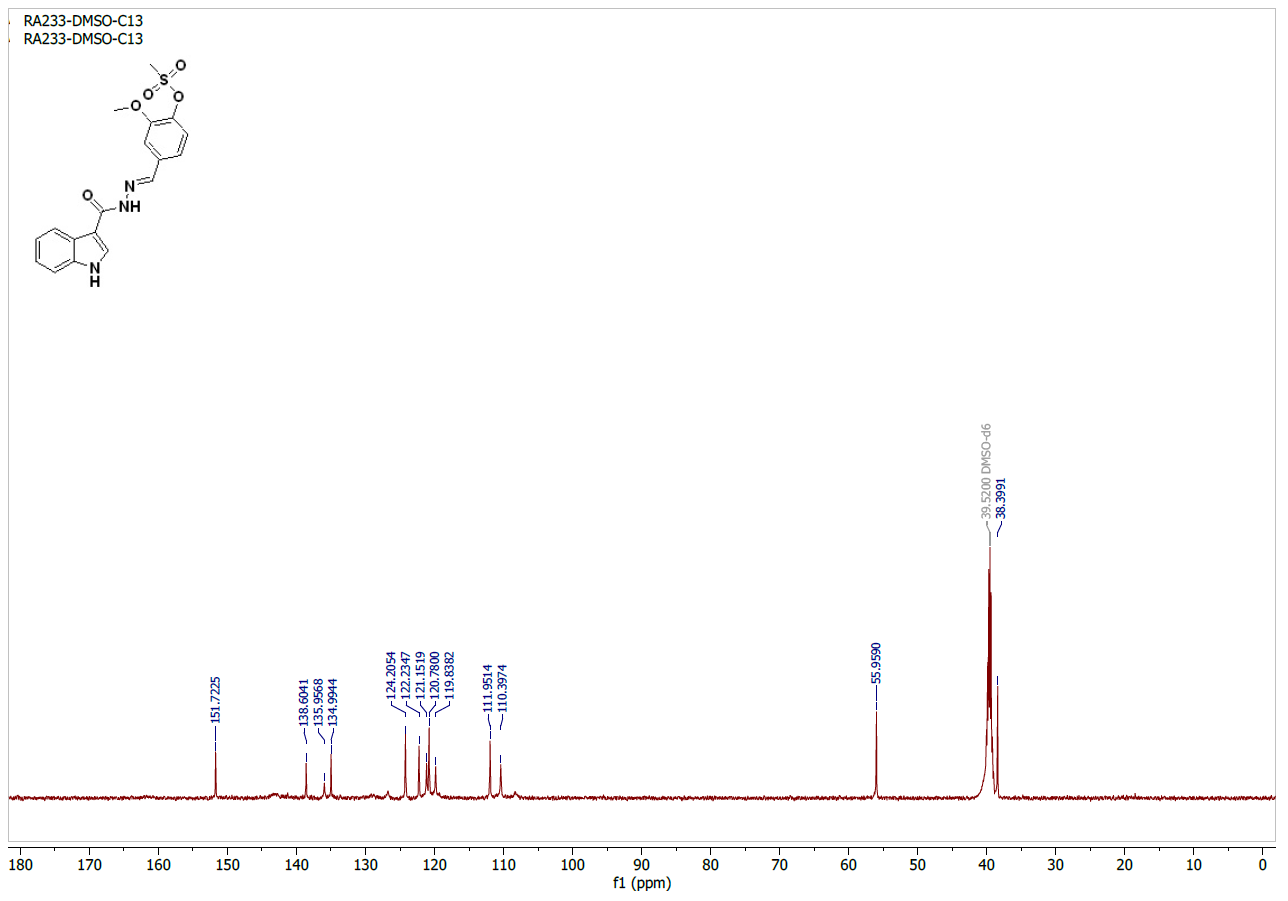


**Fig. S7.** ^13^C NMR spectrum of compound **6a**


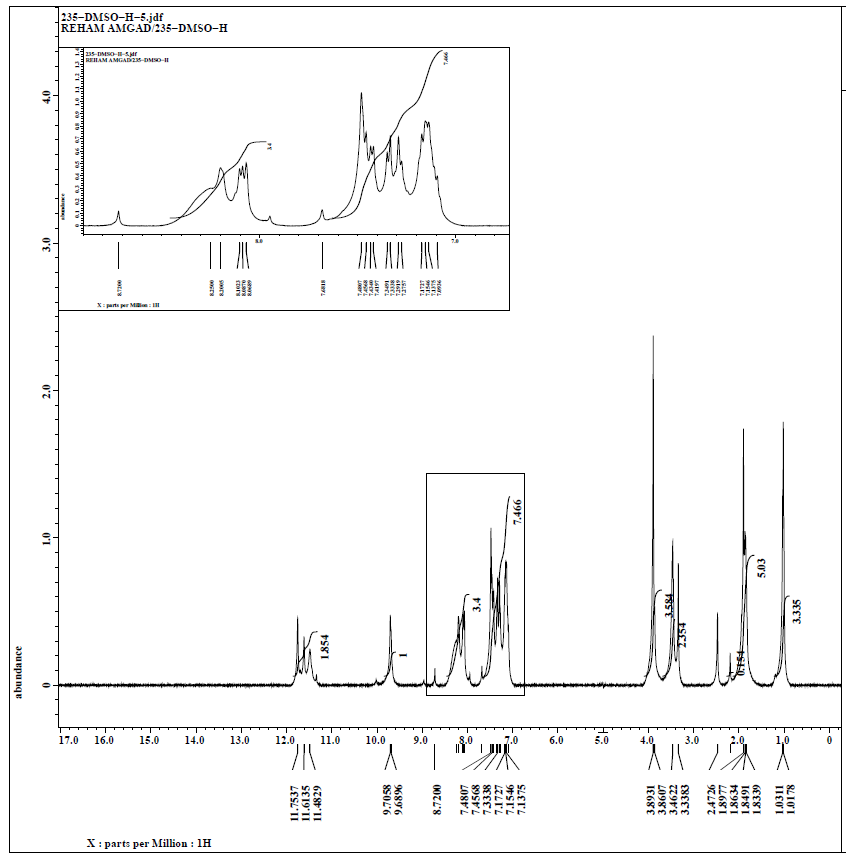


**Fig. S8.** ^13^C NMR spectrum of compound **6c**


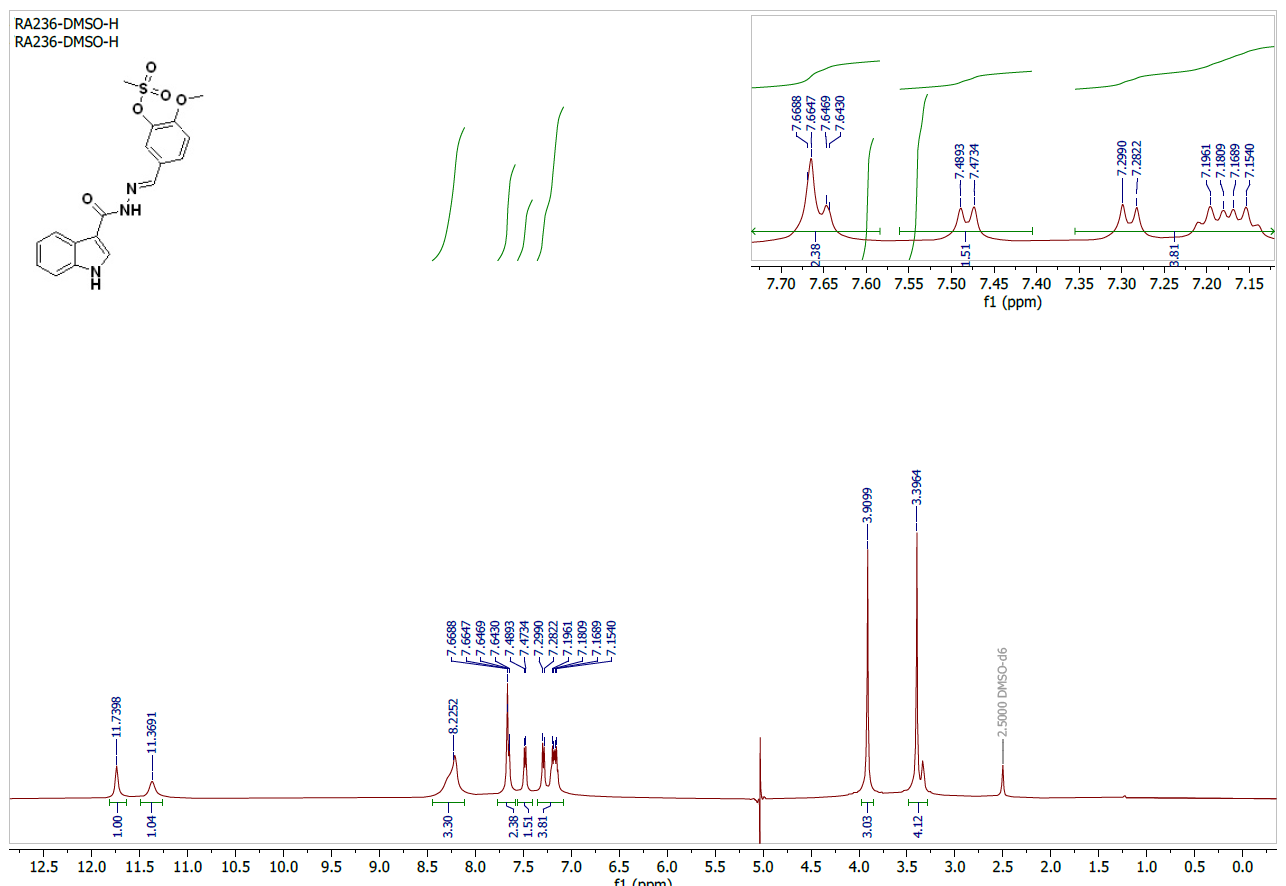


**Fig. S9.** ^1^H NMR spectrum of compound **8a**


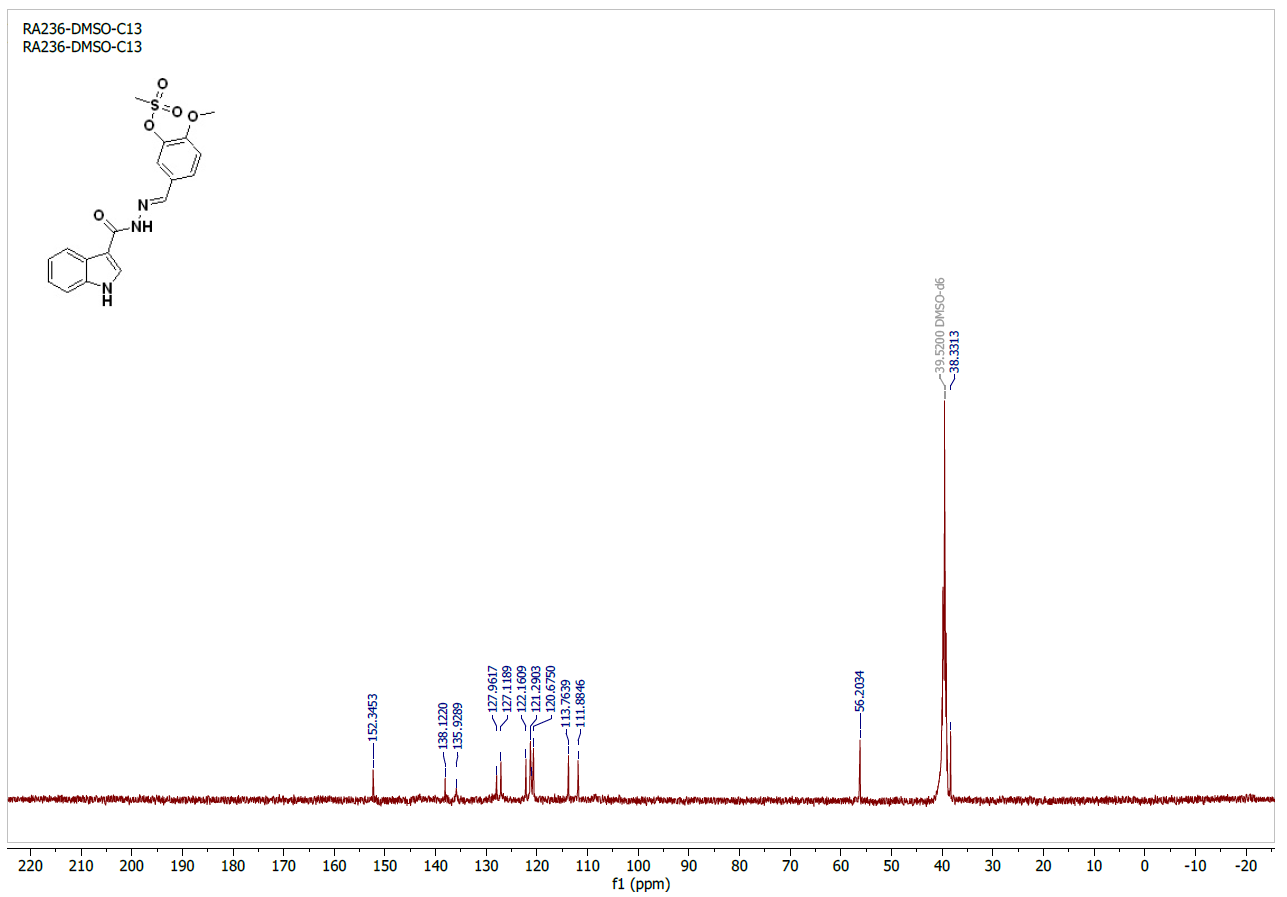


**Fig. S10.** ^13^C NMR spectrum of compound **8a**


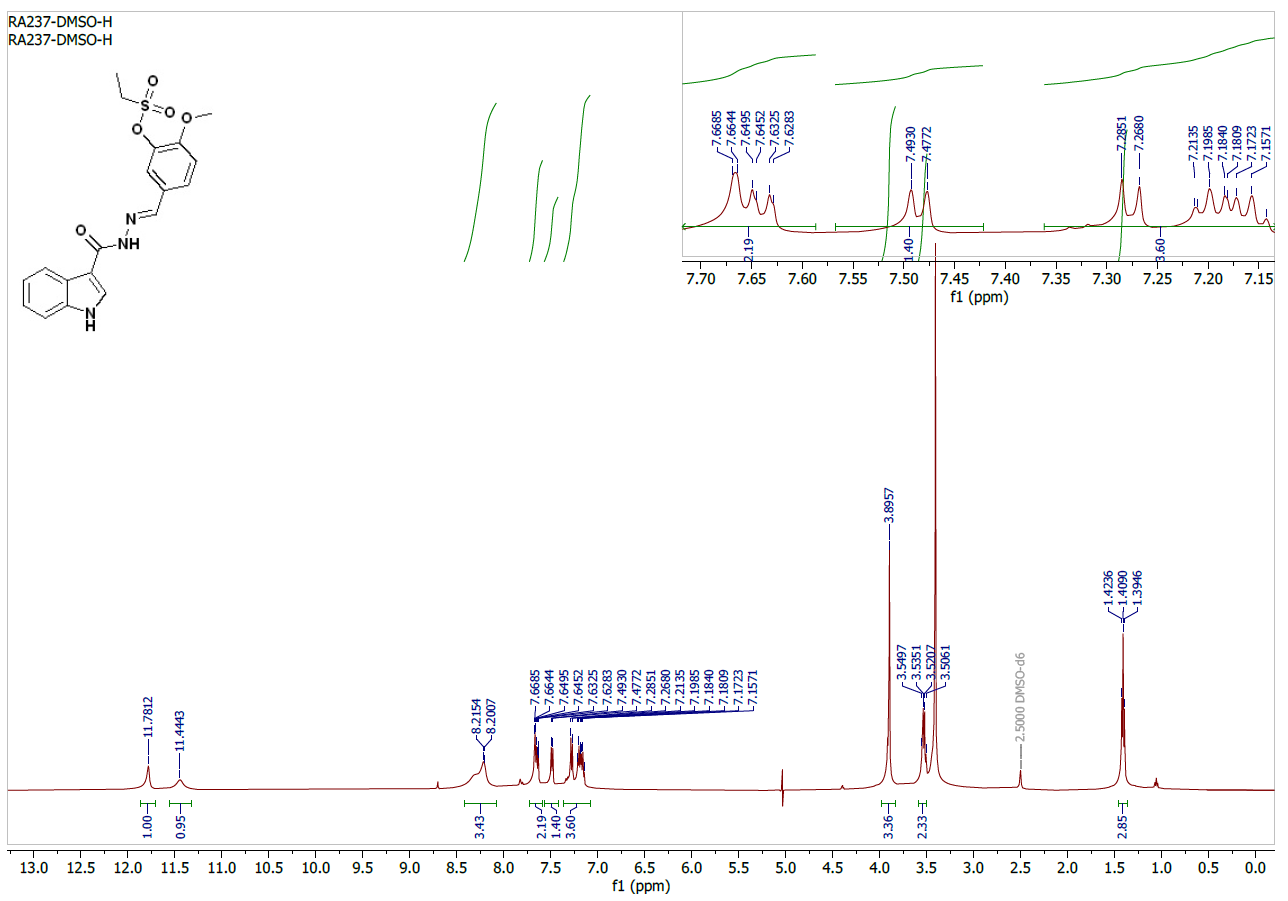


**Fig. S11.** ^1^H NMR spectrum of compound **8b**


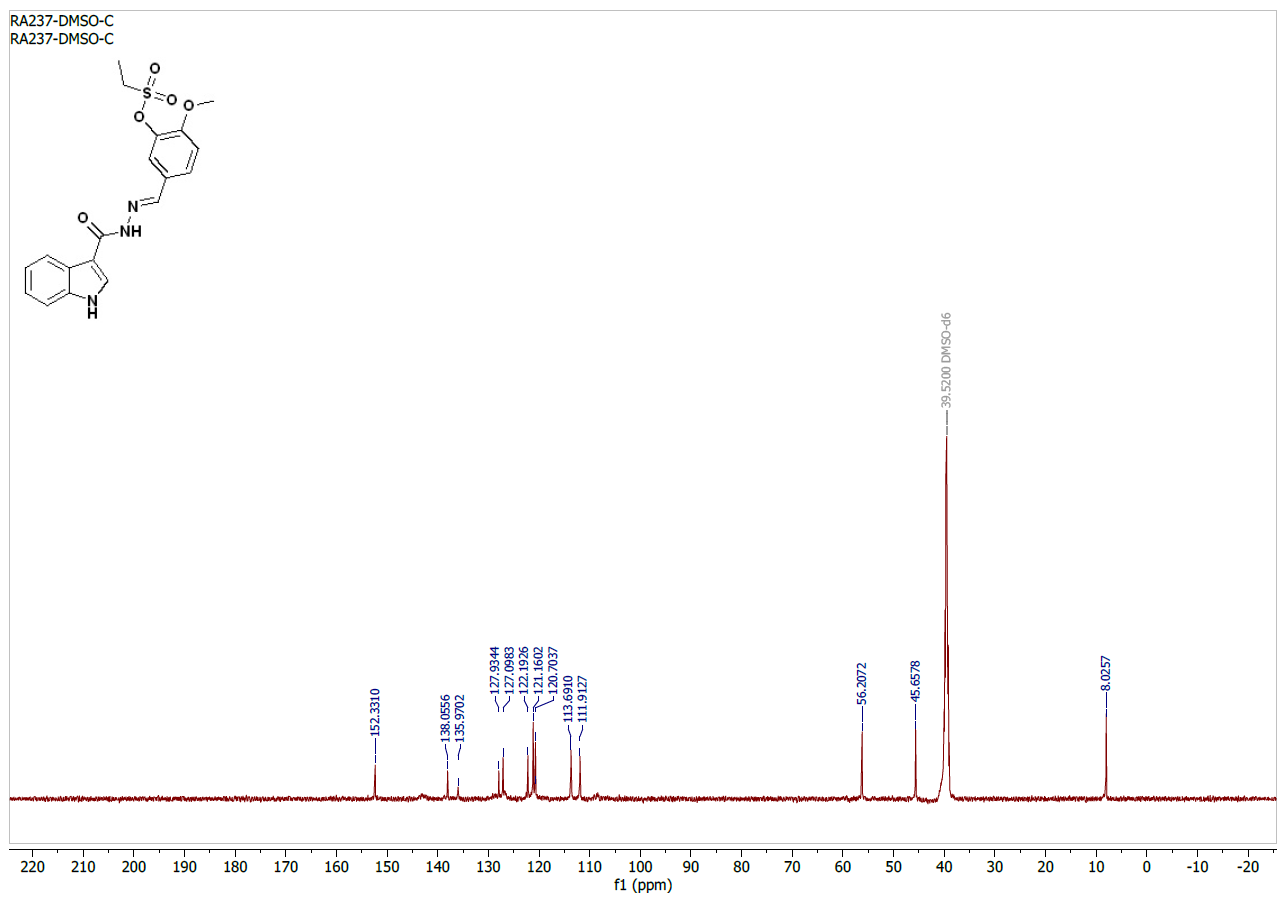


**Fig. S12.** ^13^C NMR spectrum of compound **8b**


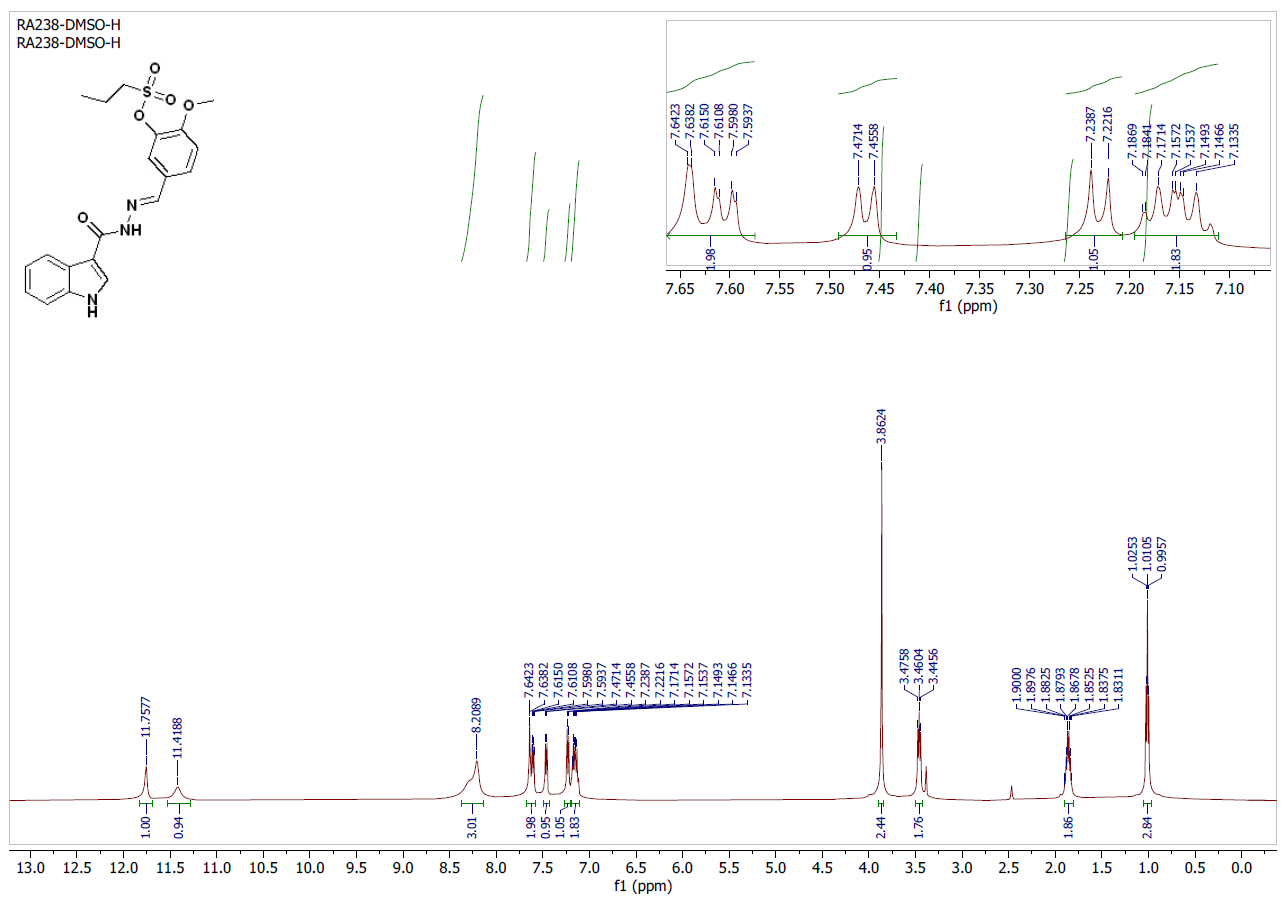


**Fig. S13.** ^1^H NMR spectrum of compound **8c**


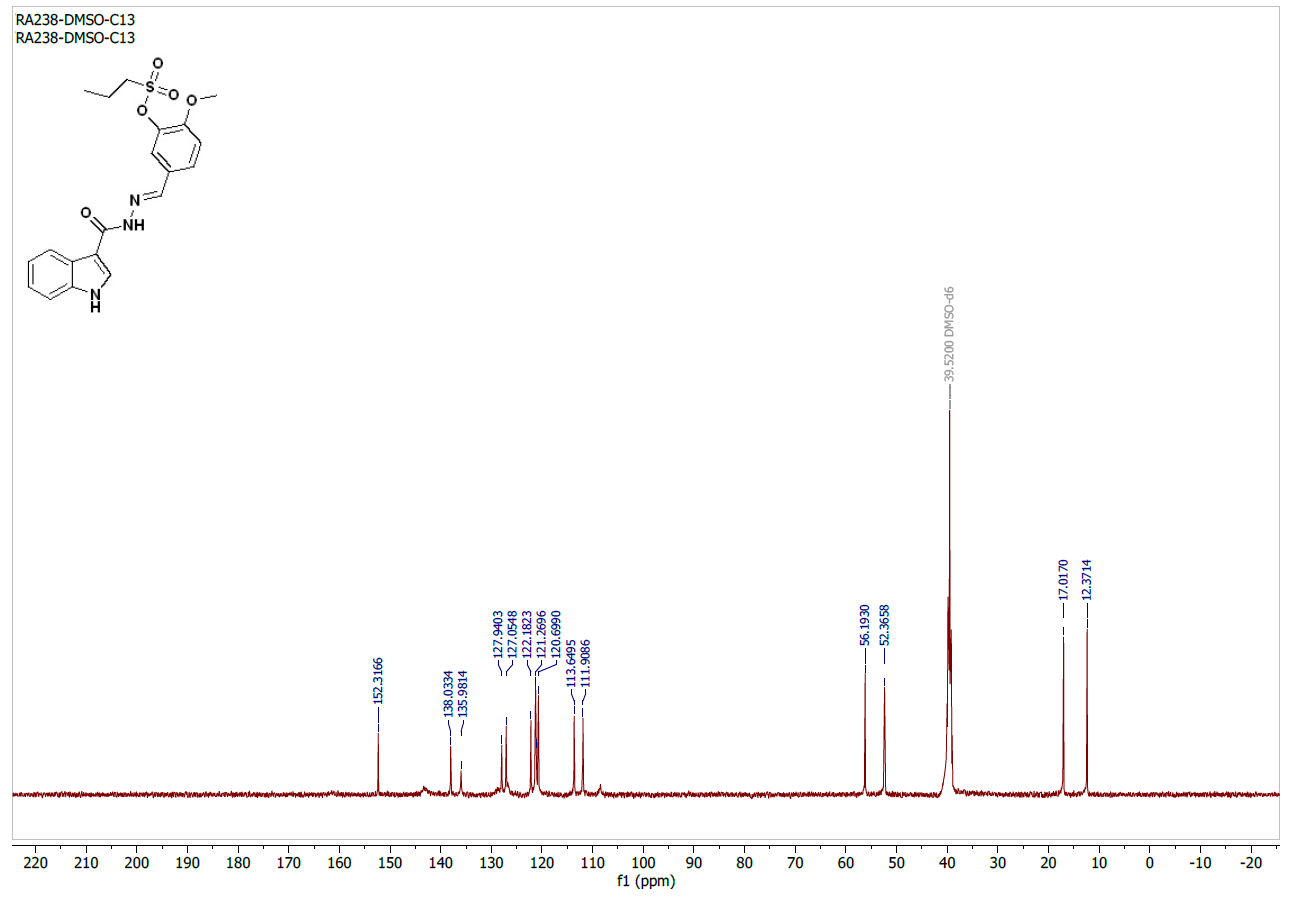


**Fig. S14.** ^13^C NMR spectrum of compound **8c**


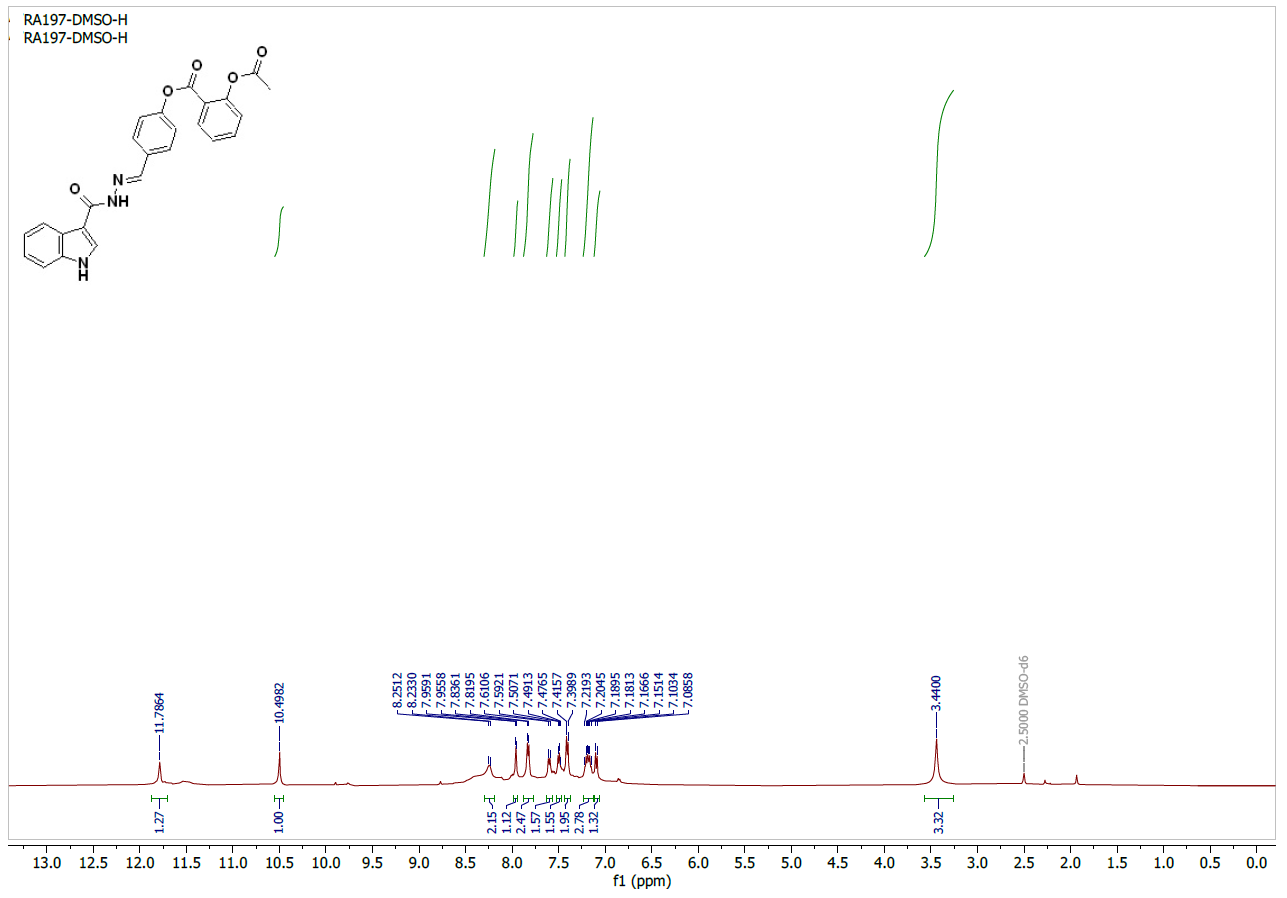


**Fig. S15.** ^1^H NMR spectrum of compound **10a**


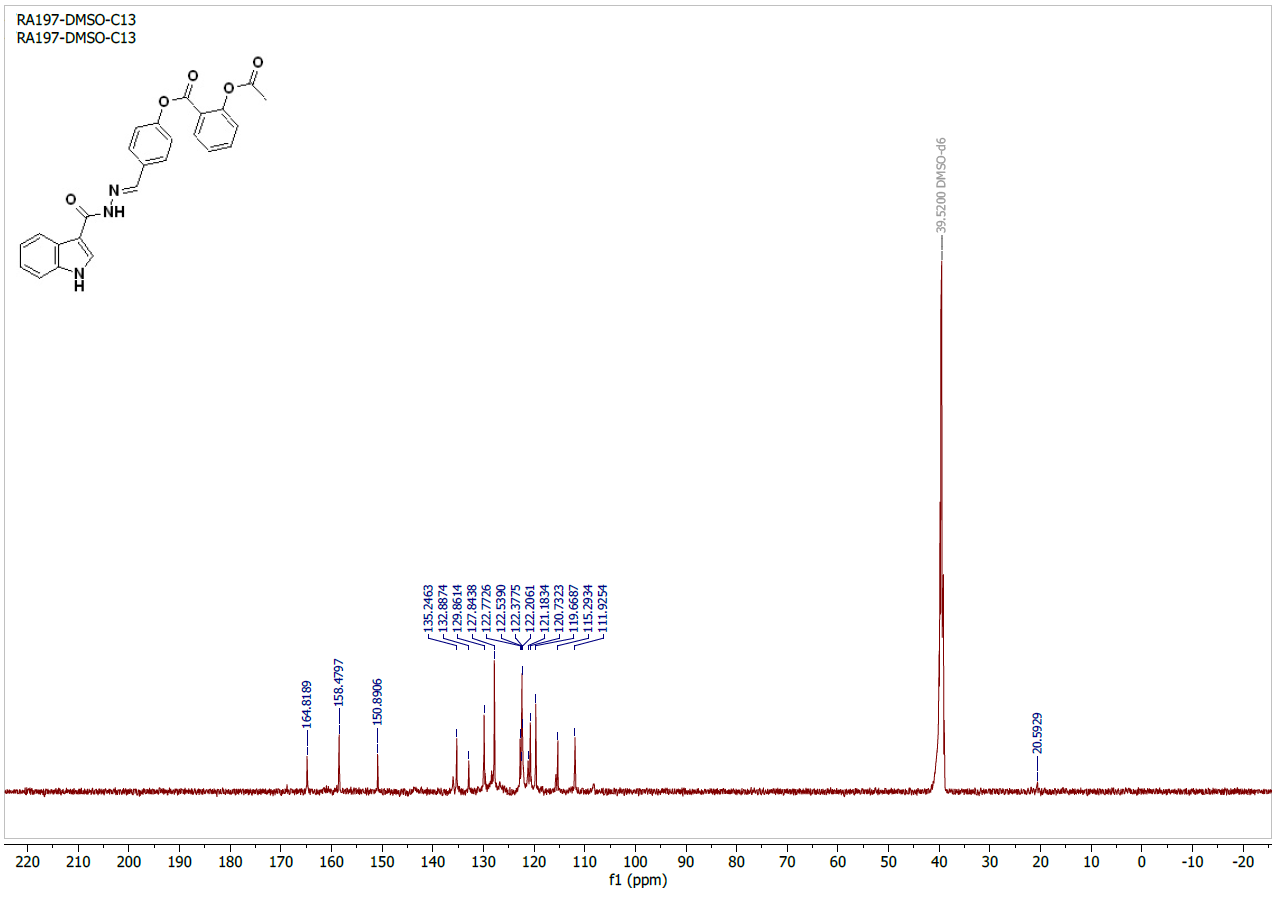


**Fig. S16.** ^13^C NMR spectrum of compound **10a**


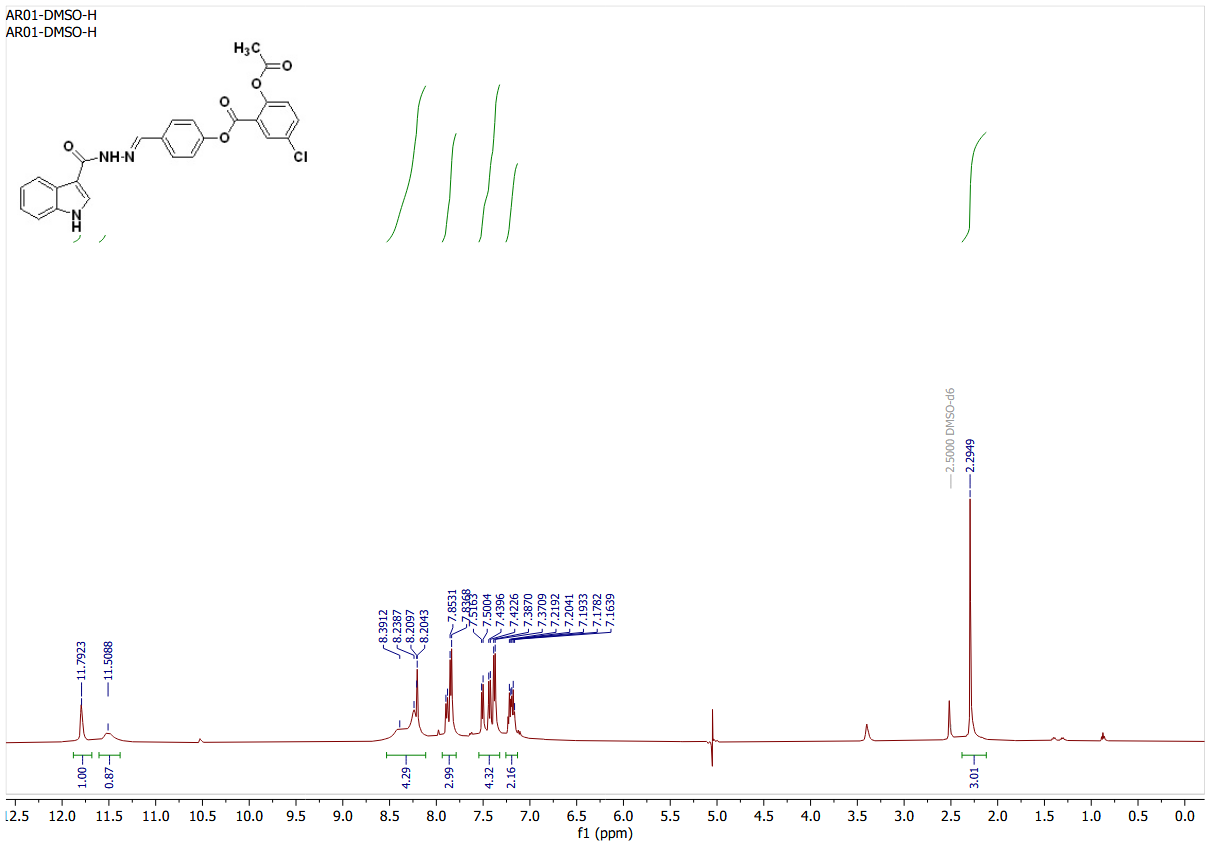


**Fig. S17.** ^1^H NMR spectrum of compound **10b**


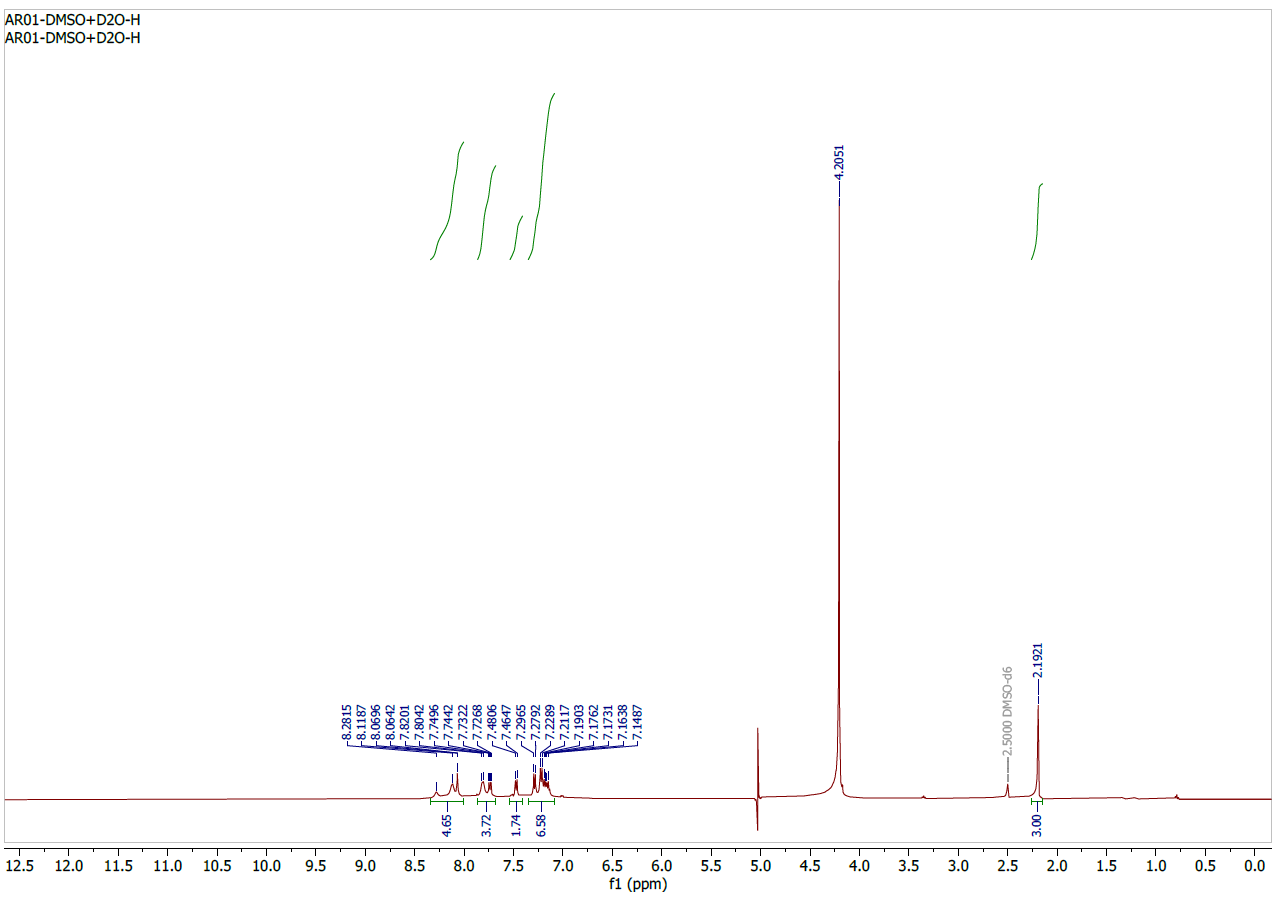


**Fig. S18.** ^1^H NMR spectrum of compound **10b**


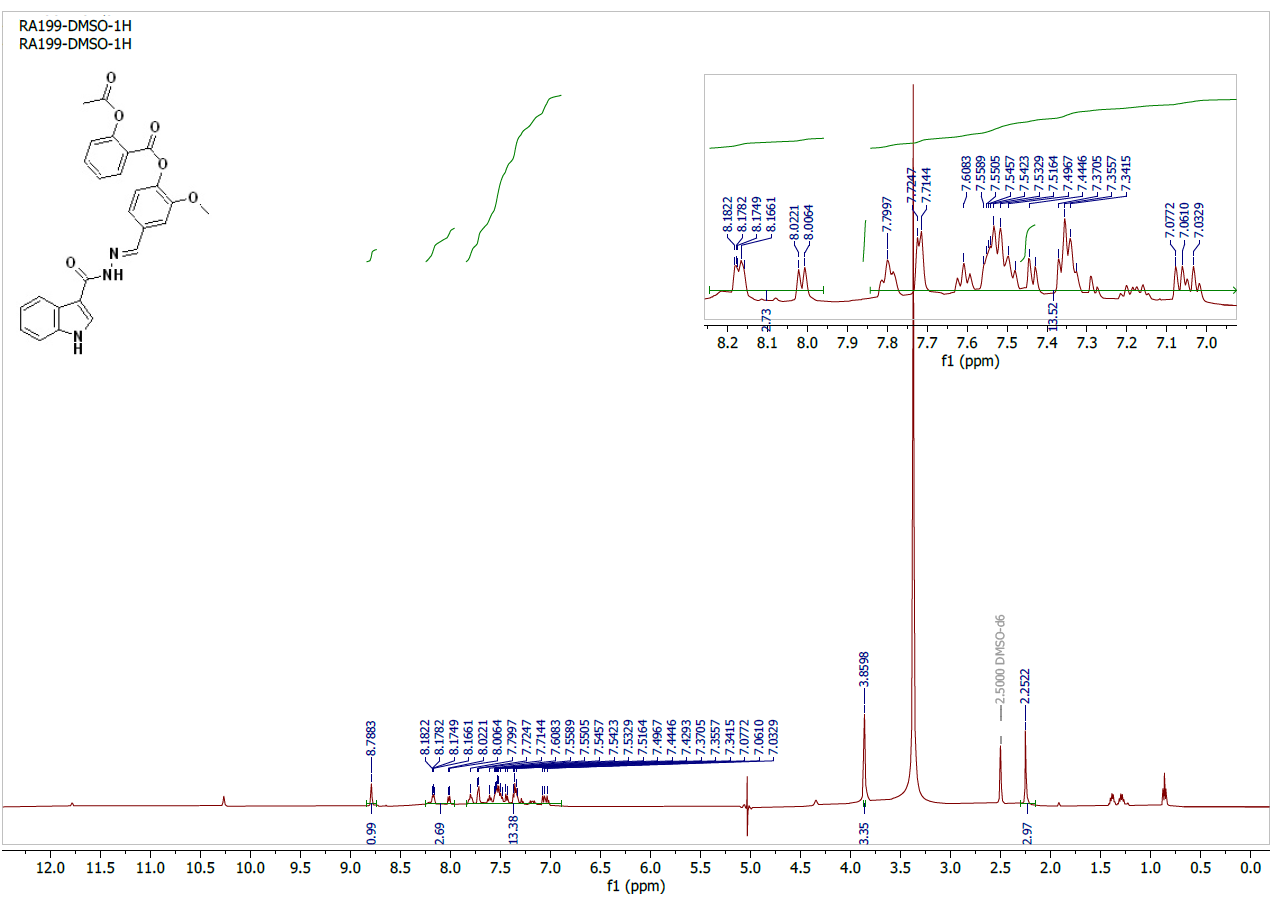


**Fig. S19.** ^1^H NMR spectrum of compound **12**


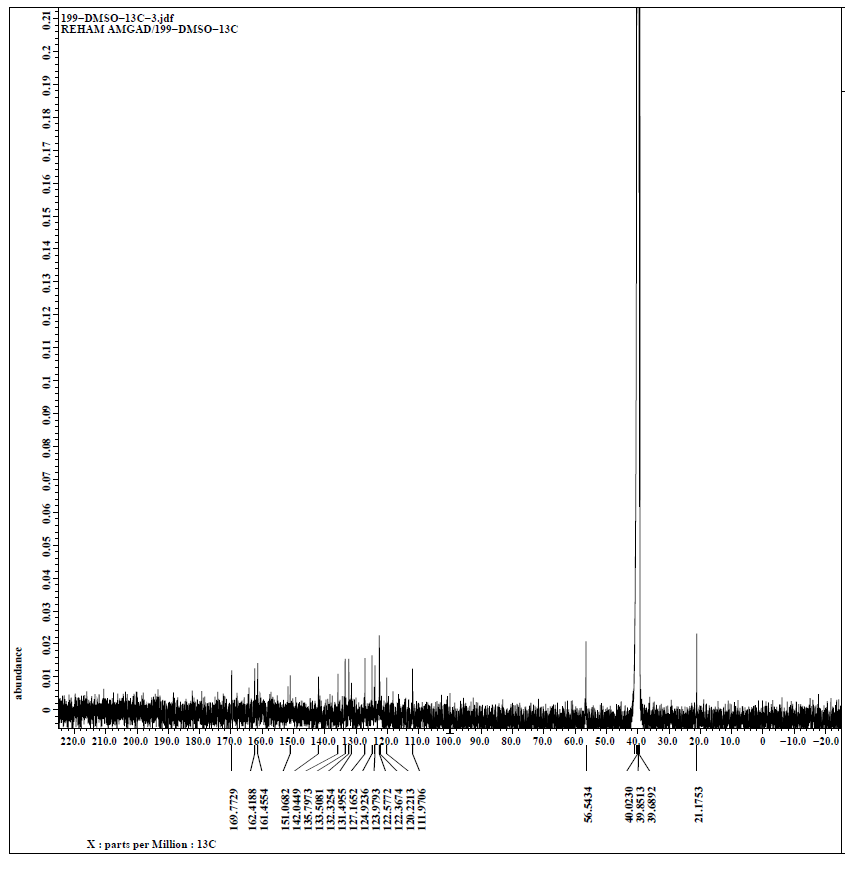


**Fig. S20.** ^13^C NMR spectrum of compound **12**


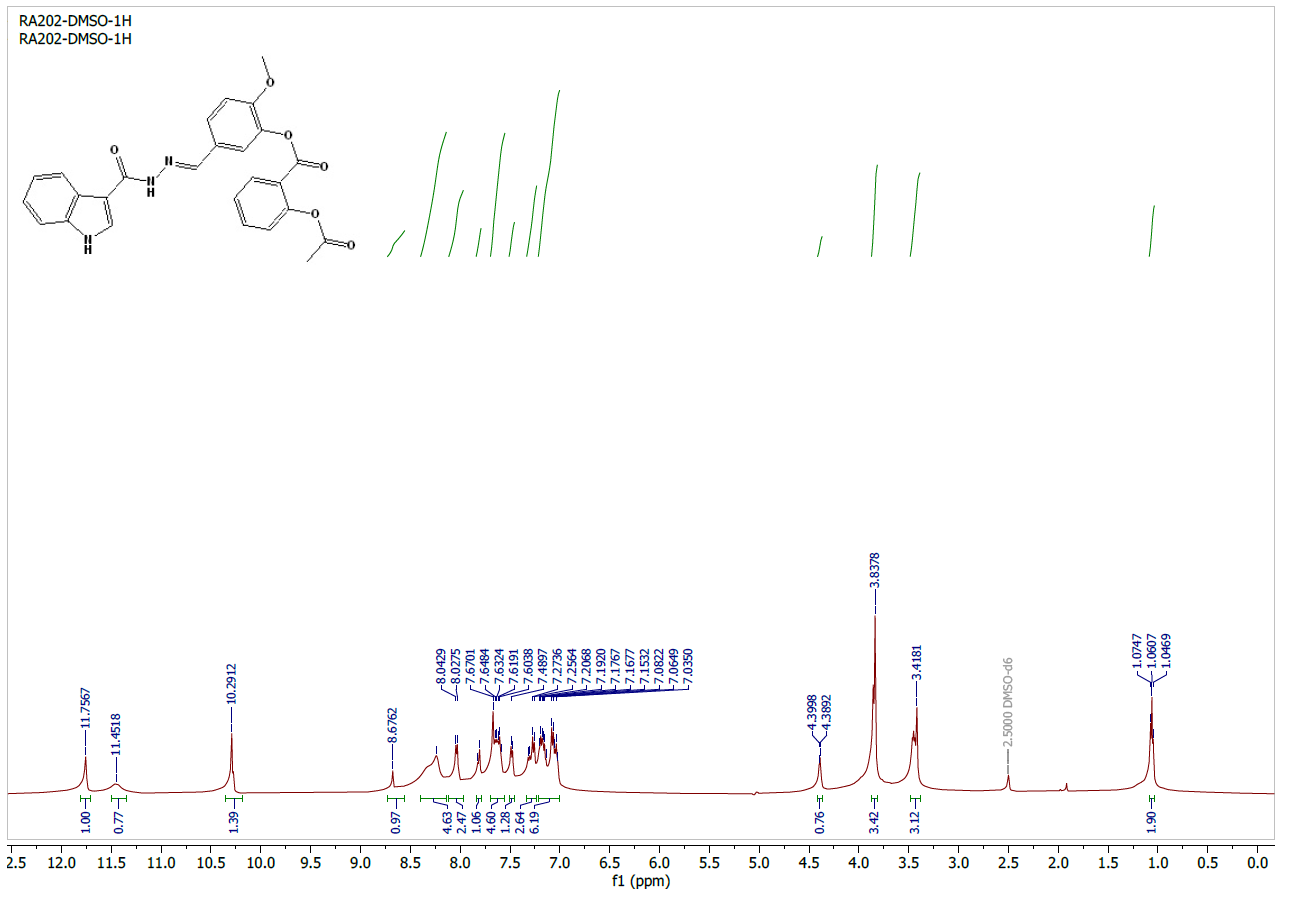


**Fig. S21.** ^1^H NMR spectrum of compound **14a**


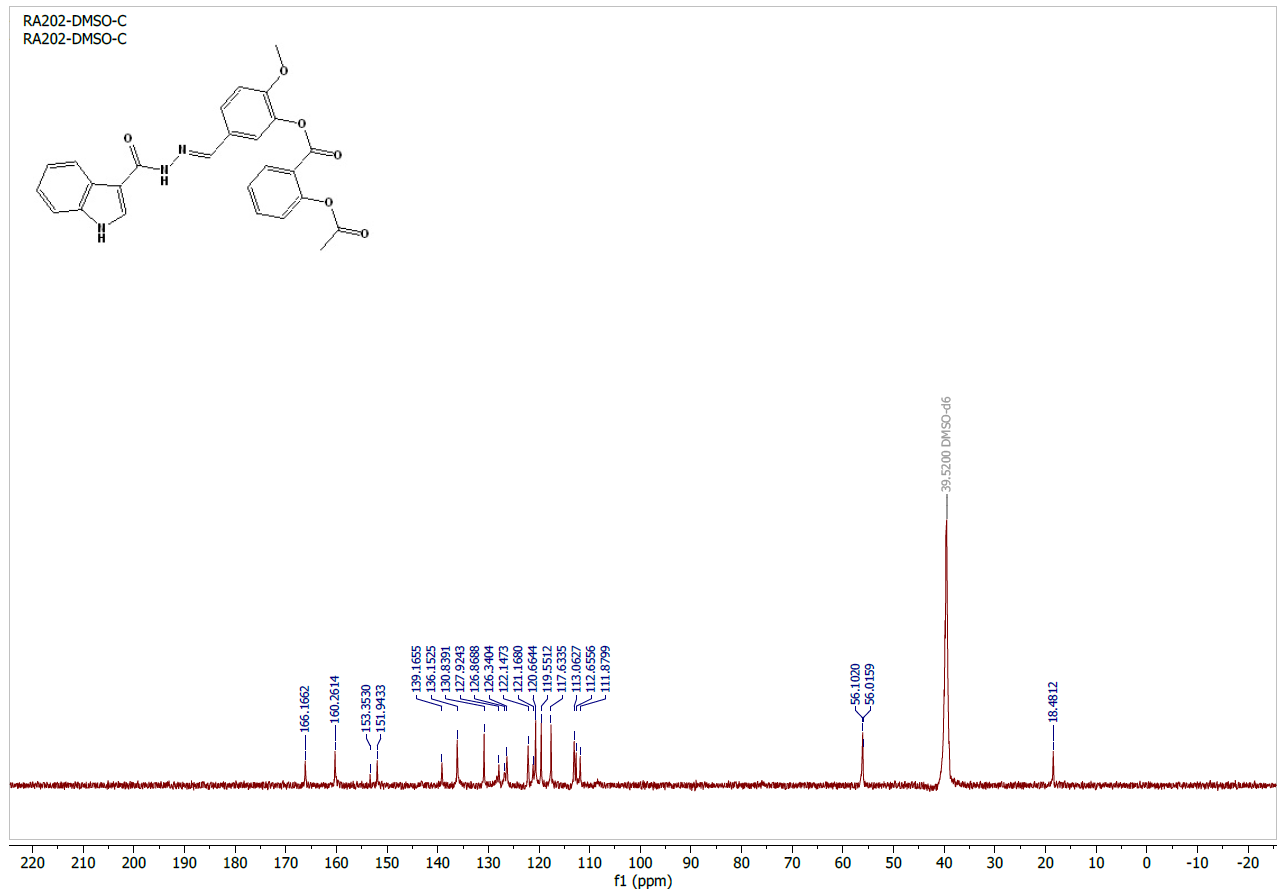


**Fig. S22.** ^13^C NMR spectrum of compound **14b**


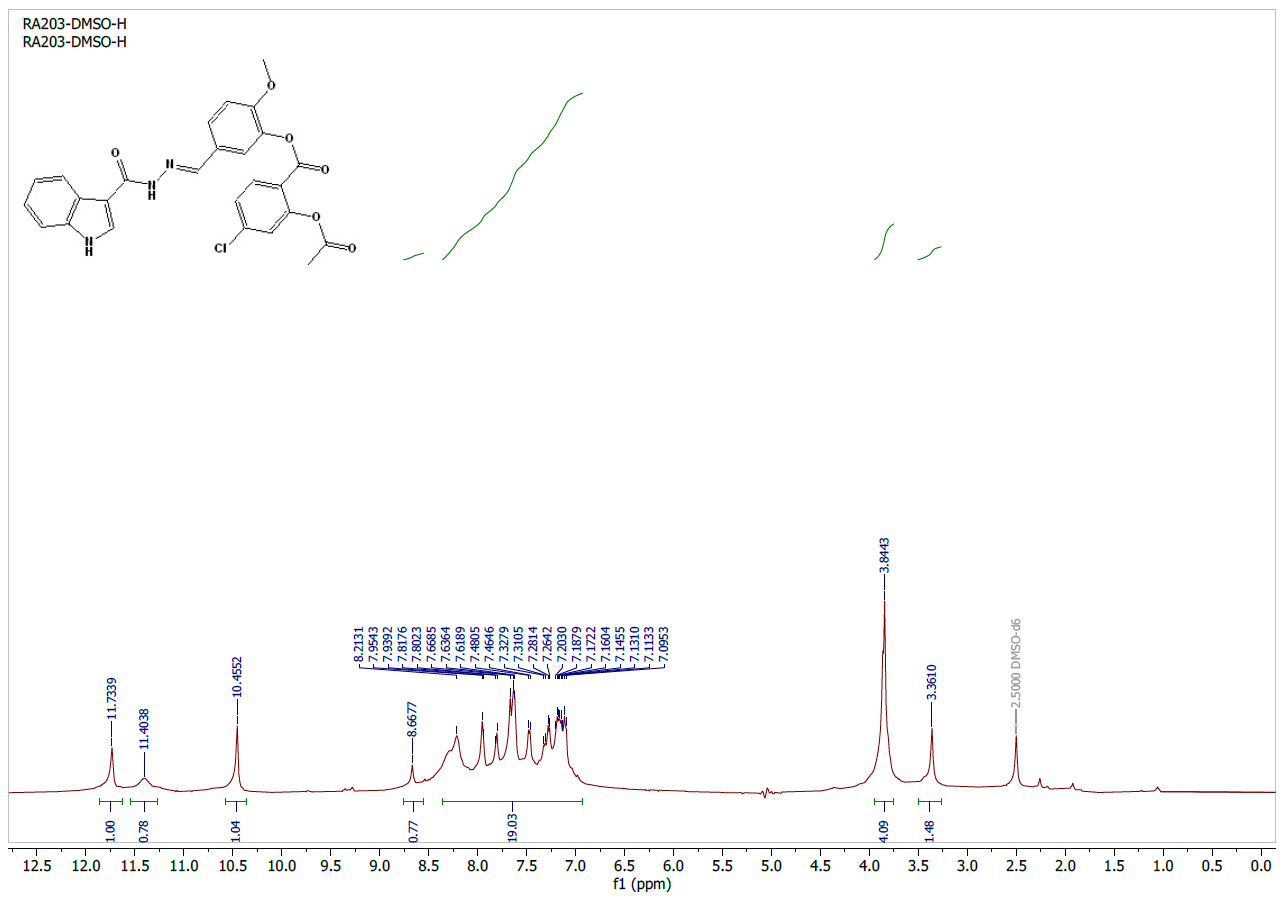


**Fig. S23.** ^1^H NMR spectrum of compound **14b**


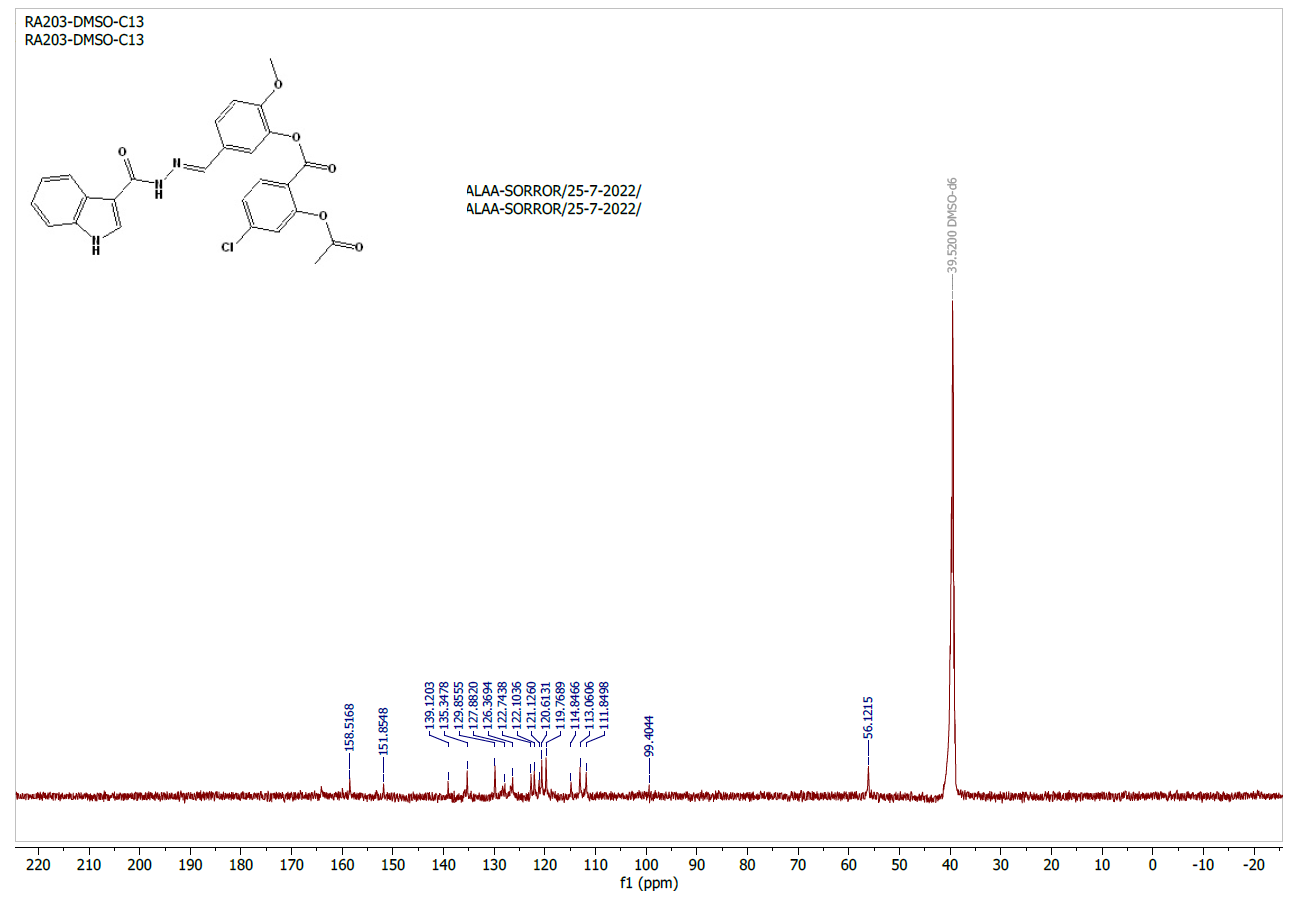


**Fig. S24.** ^13^C NMR spectrum of compound **14b**


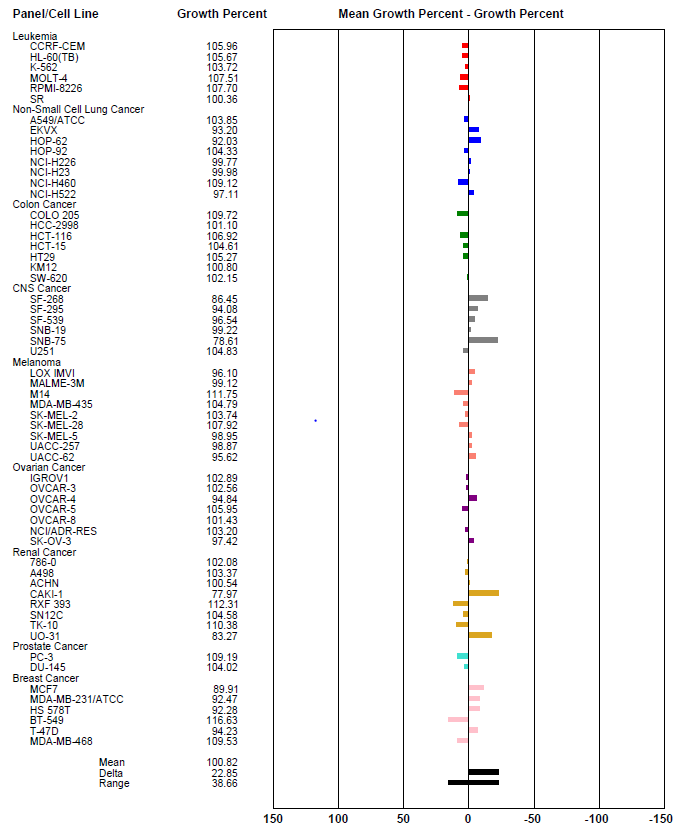


**Fig. S25.** Anticancer screening data of compound **4a** in concentration 10^−5^ M.


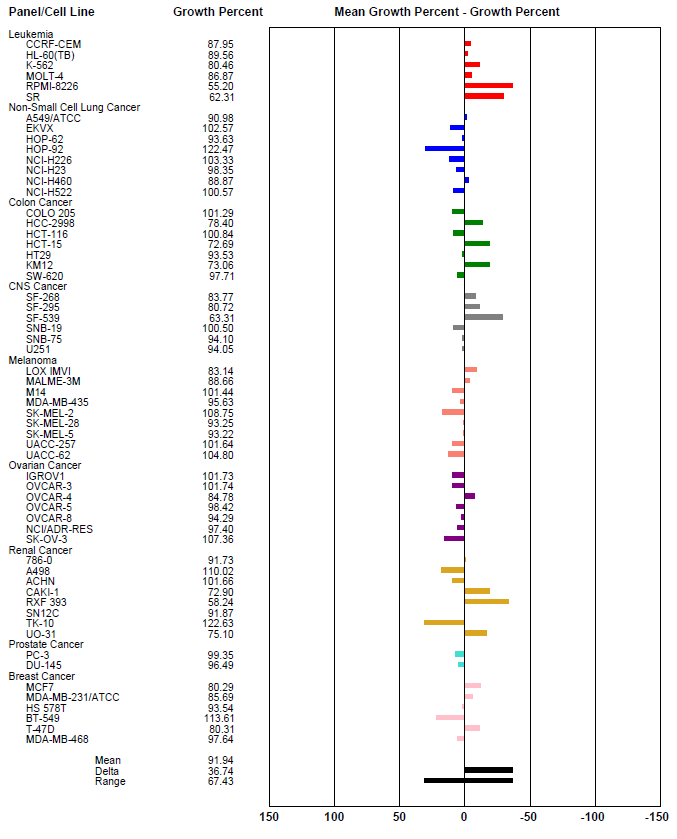


**Fig. S26.** Anticancer screening data of compound **4b** in concentration 10^−5^ M.


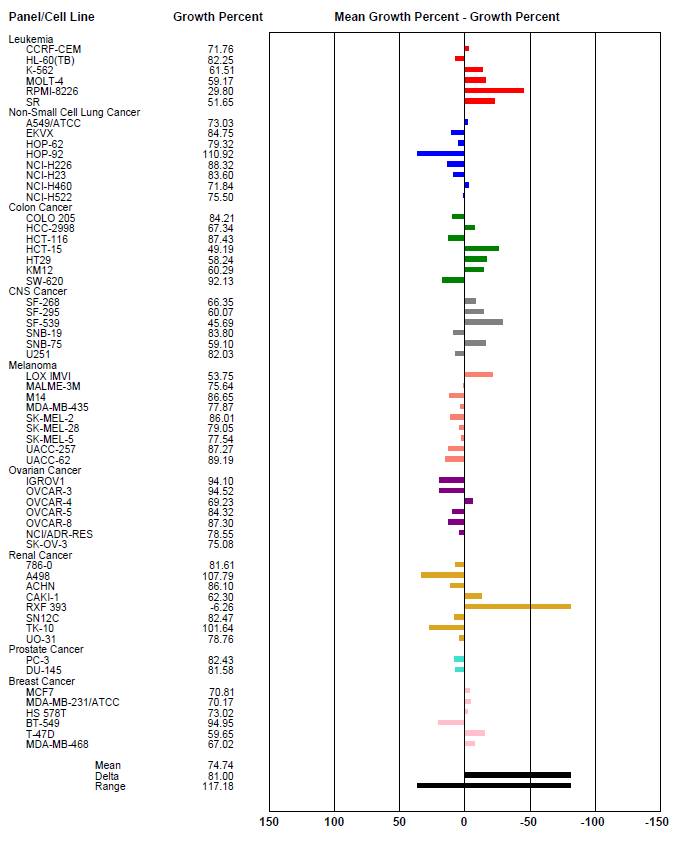


**Fig. S27.** Anticancer screening data of compound **4c** in concentration 10^−5^ M.


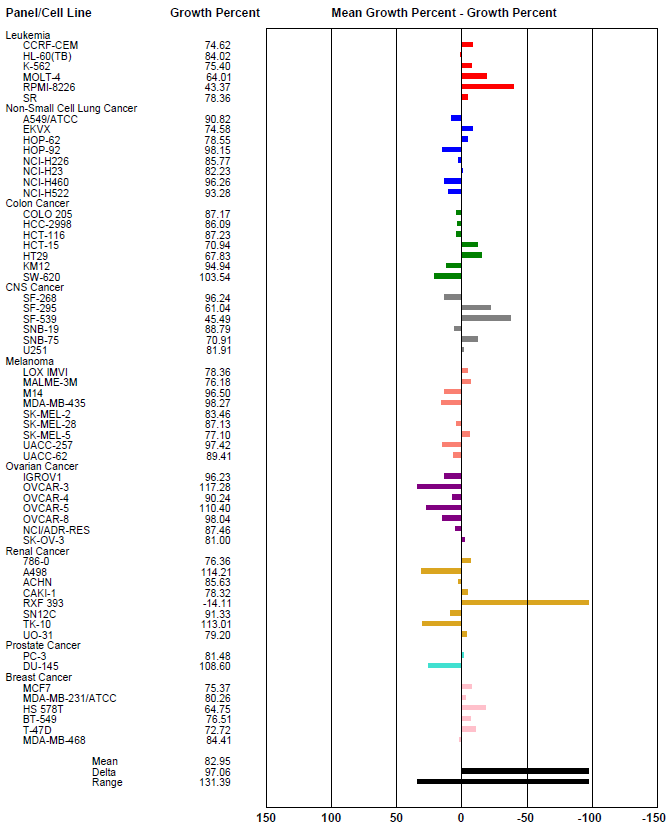


**Fig. S28.** Anticancer screening data of compound **6c** in concentration 10^−5^ M.


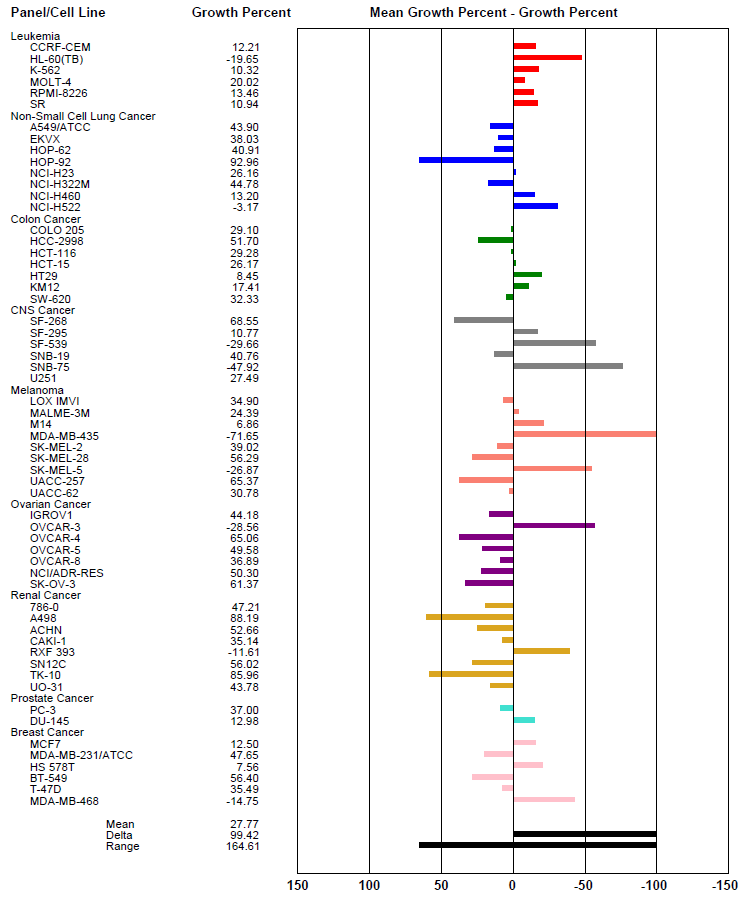


**Fig. S29.** Anticancer screening data of compound **8a** in concentration 10^−5^ M.


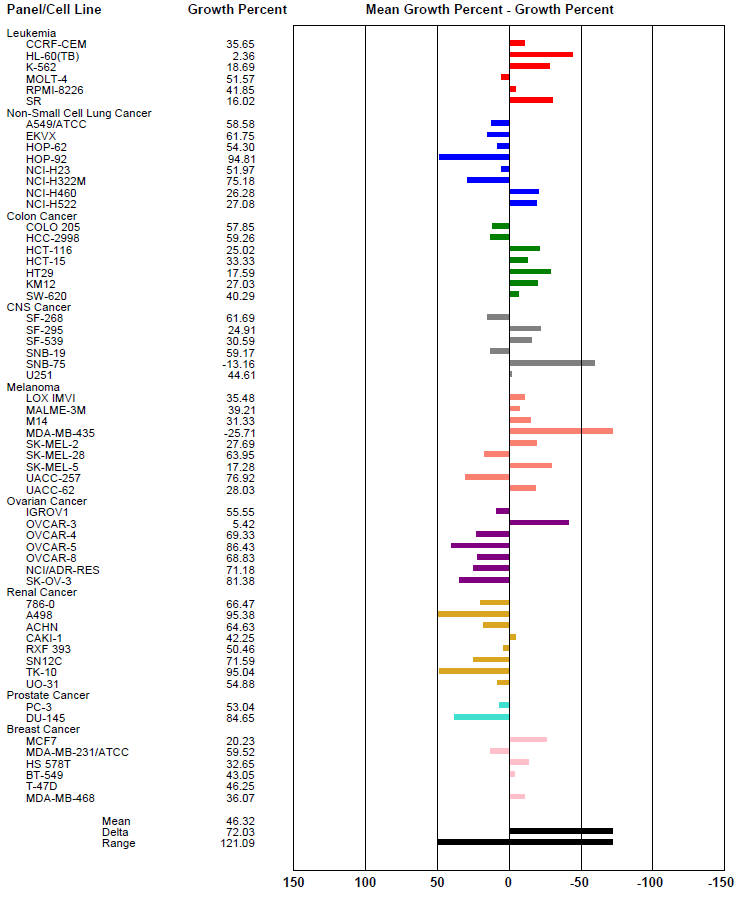


**Fig. S30.** Anticancer screening data of compound **8b** in concentration 10^−5^ M.


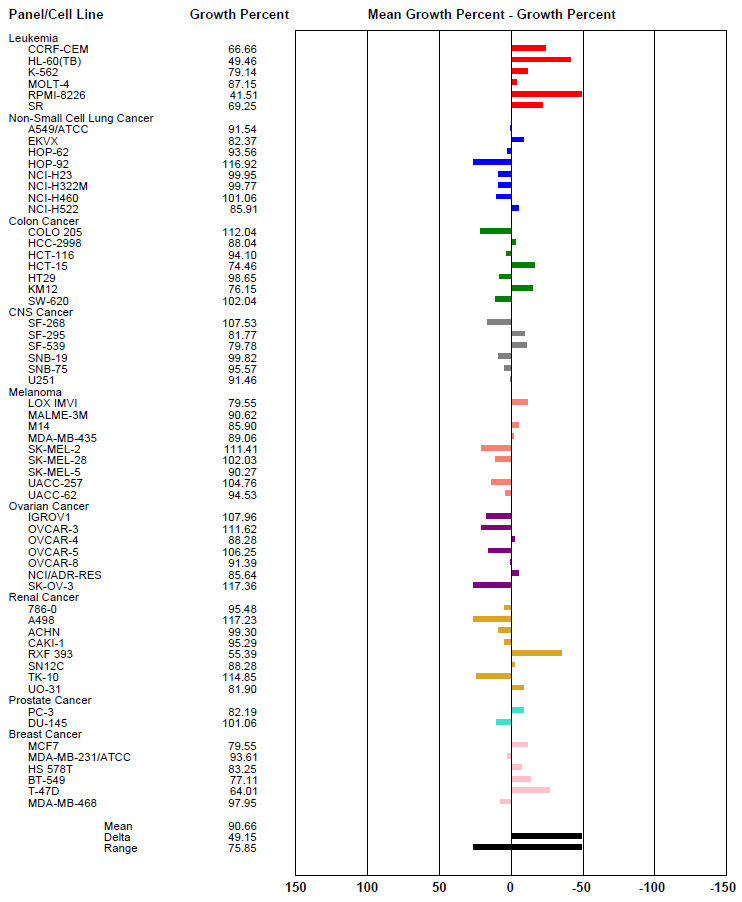


**Fig. S31.** Anticancer screening data of compound **8c** in concentration 10^−5^ M.


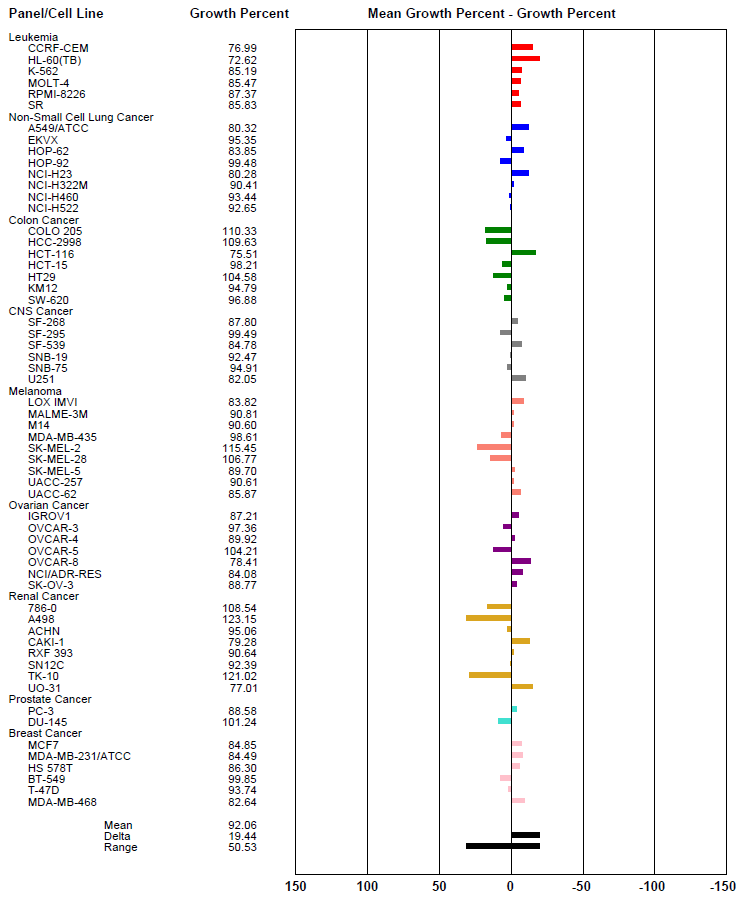


**Fig. S32.** Anticancer screening data of compound **10a** in concentration 10^−5^ M.


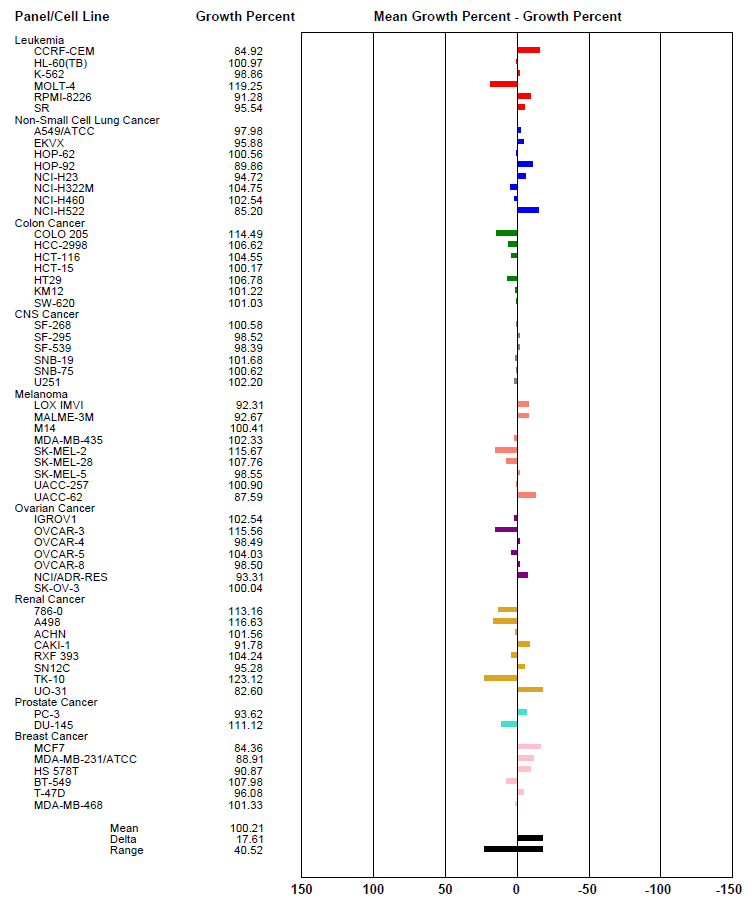


**Fig. S33.** Anticancer screening data of compound **12** in concentration 10^−5^ M.


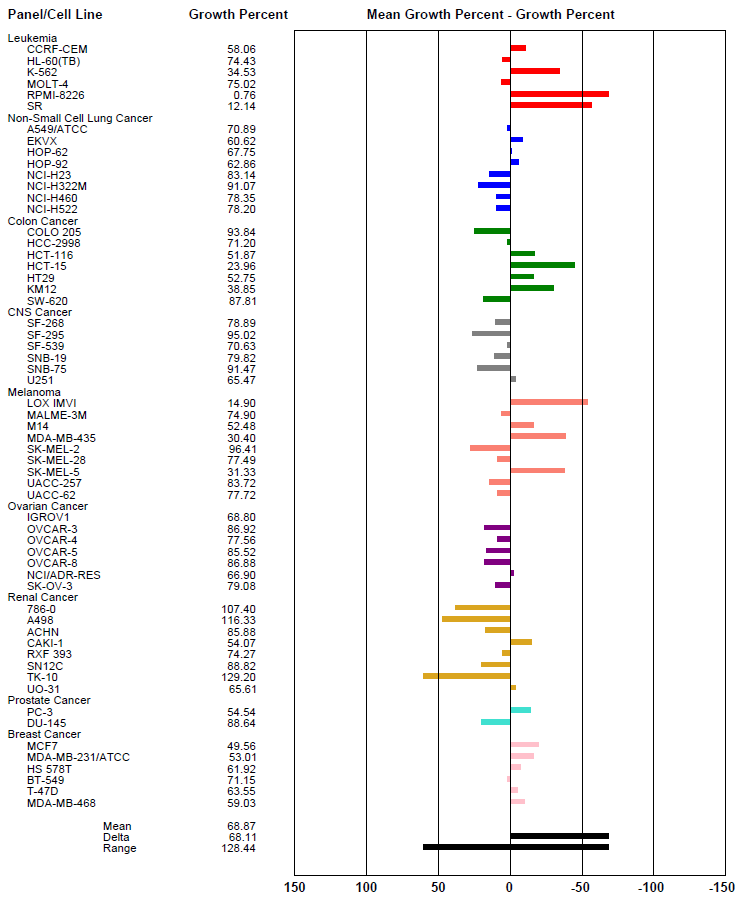


**Fig. S34.** Anticancer screening data of compound **14a** in concentration 10^−5^ M.


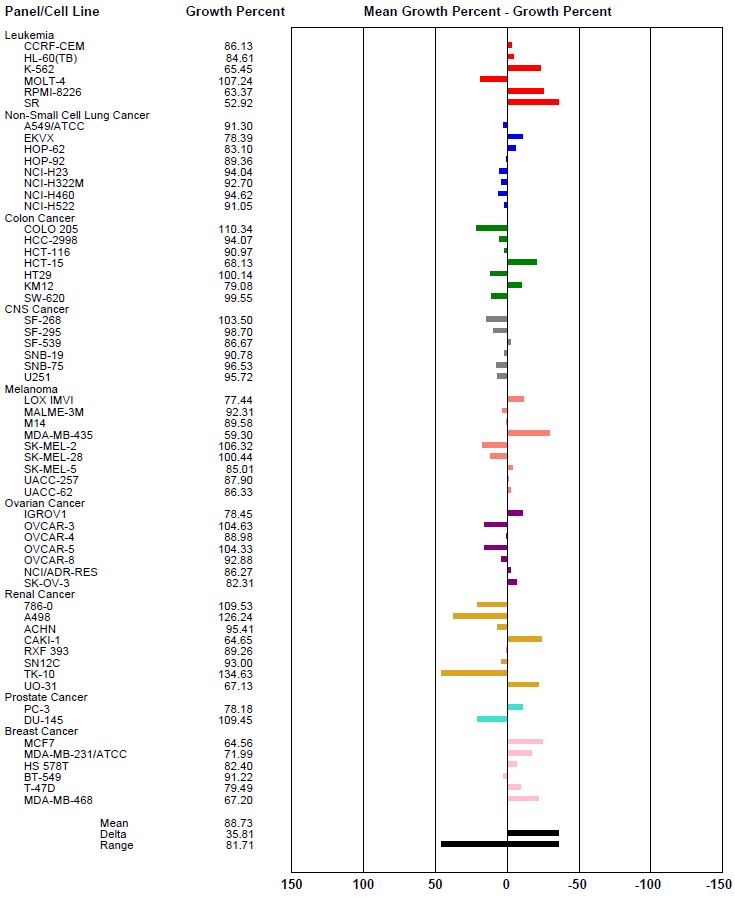


**Fig. S35.** Anticancer screening data of compound **14b** in concentration 10^−5^ M.


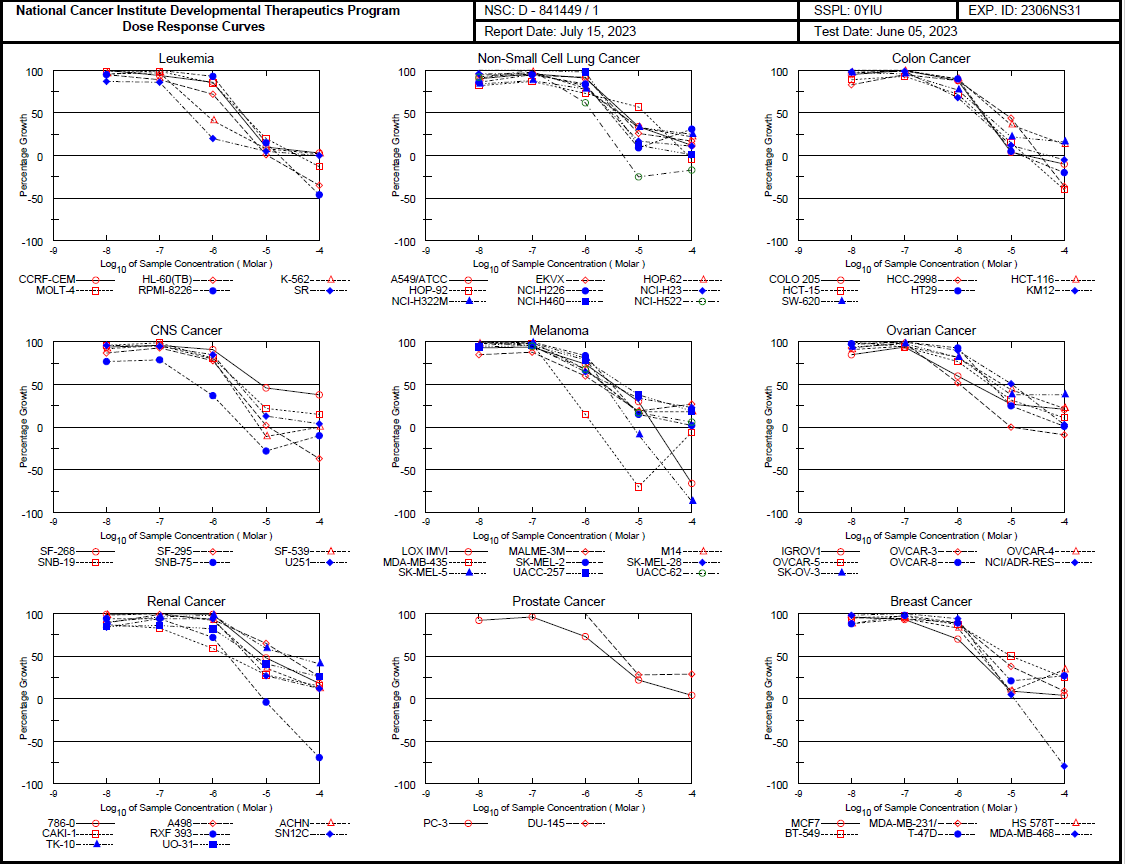


**Fig. S36.** Graphical presentation of growth inhibition of **8a** at five-dose concentrations (0.01, 0.1, 1, 10, & 100 *µ*M) after 48 hours using SRB assay at NCI.


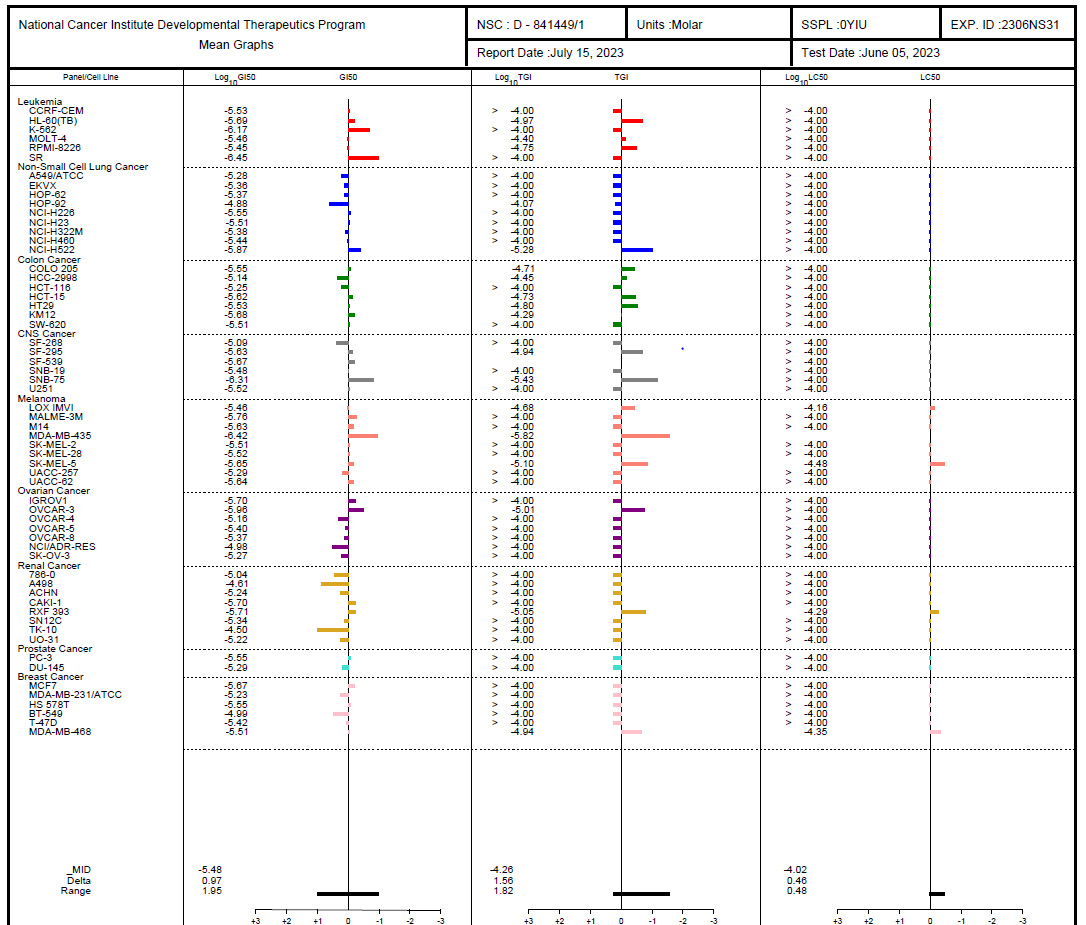


**Fig. S37.** GI_50_, TGI, & LC_50_ values of **8a** at five-dose level.


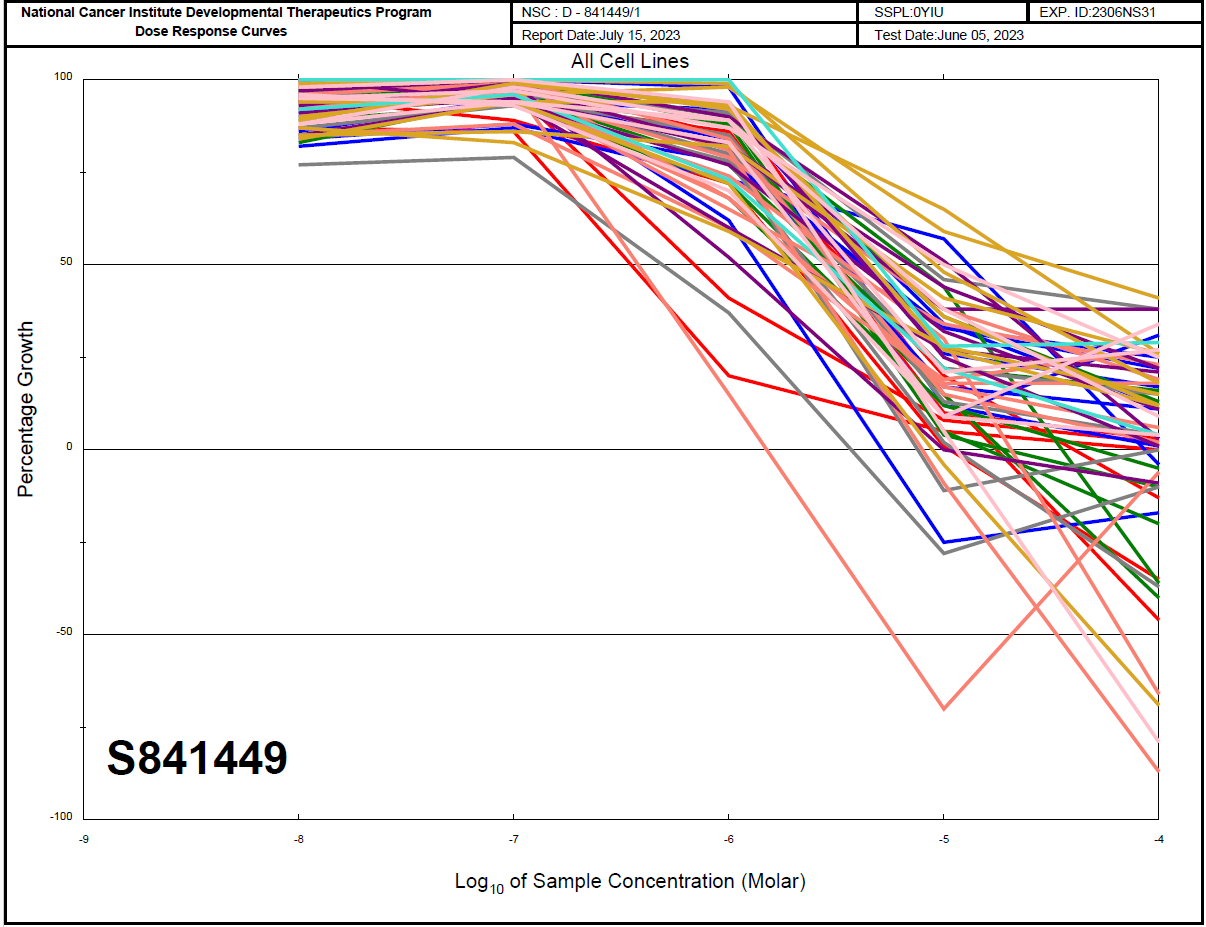


**Fig. S38.** Dose response curves of **8a** at five-dose concentrations (0.01, 0.1, 1, 10, & 100 *µ*M) after 48 hours using SRB assay at NCI.


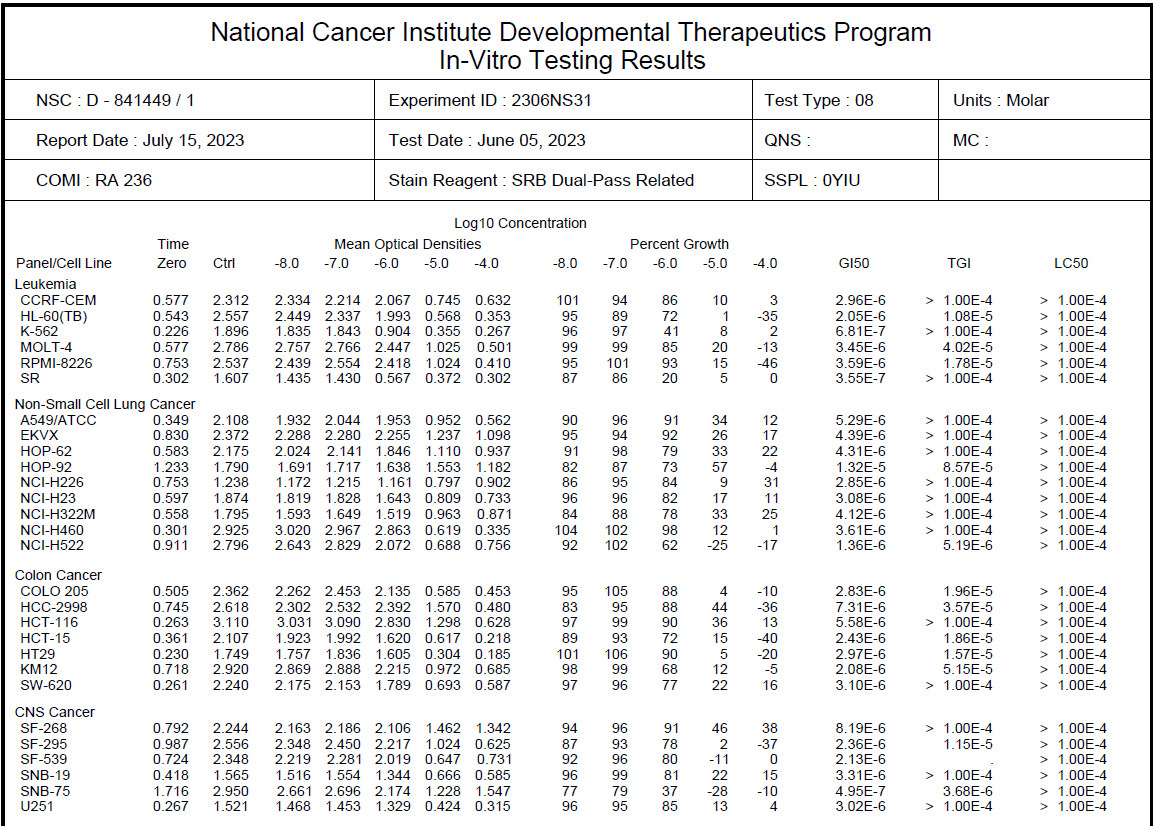


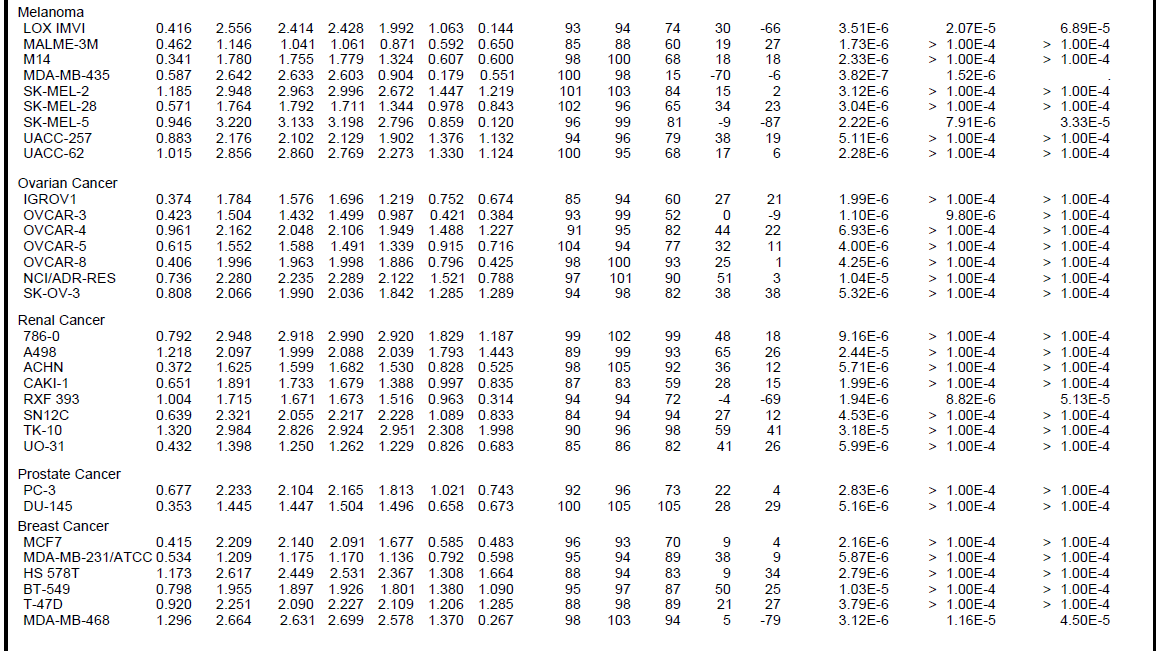


**Fig. S39.**


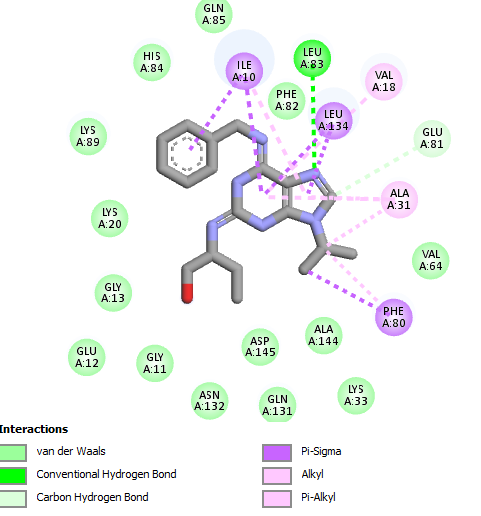


The predicted 2D-binding interactions of **Roscovitine** in the ATP-binding pocket (PDB code: 6GUH).

**Fig, S40**


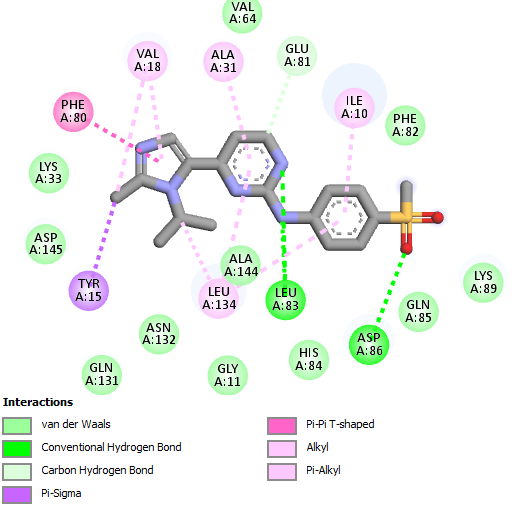


The predicted 2D-binding interactions of **AZD5438** in the ATP-binding pocket (PDB code: 6GUH).

***In vitro* enzymatic inhibitory evaluation against CDK-2**

**Principal**

The CDK2 assay kit purchased from Bioscience company was designed to measure CDK2/CyclinA2 activity using Kinase-Glo MAX as a detection reagent. The CDK2 assay Kit was performed by using white 96-well plate, with enough purified recombinant CDK2/CyclinA2 enzyme, CDK substrate peptide, ATP and kinase assay buffer. The Kinase-Glo MAX assay is a luminescent kinase assay that measures ADP formed from a kinase reaction; ADP is converted into ATP, which is converted into light that was detected spectrophotometrically.

**Procedure**

1-Prepare the master mixture: 6 μl of (5x Kinase assay buffer) + 1μl ATP (500 μM) + 5 μl (10XCDK substrate peptide) + 13 μl distilled water. 25 μl were added to every well.

2- Prepare the Inhibitor solution by dissolving the tested inhibitor compound in DTT and making different concentrations by using (1x Kinase assay buffer).

3-Add 5 μl of Inhibitor solution to the “Test Inhibitor” wells. At the same time, add 5 μl of without the tested inhibitor compound to the “Positive Control" and “Blank” wells.

4- The reaction was started by adding 20 μl of diluted CDK2/Cyclin A2 enzyme to the “Positive Control” and "Test Inhibitor" wells. On the other hand, add 20 μl of (1x Kinase assay buffer) to the "Blank" wells.

5- Incubate the micro-plate at 30°C for 45 minutes.

6-After the 45 minutes reaction, add 50 μl of Kinase-Glo Max reagent to each well. Cover plate with aluminum foil and incubate the plate at room temperature for 15 minutes.

7-Measure luminescence using the microplate reader. The Blank reading value must be subtracted for all wells.

8- A standard curve will be plotted by using the log of different dilutions of the enzyme (log [conc]) against the obtained inhibitory percent to obtain the IC50 value. Roscovetine was used as a standard CDK2/Cyclin A2 inhibitor.

**Docking Procedure:**

1. The three-dimensional crystal structure of the target CDK-2, co-crystallized with its respective native ligand inhibitor AZD 5438, was retrieved from the Protein Data Bank (PDB) <https://www.rcsb.org> under accession (PDB ID: 6GUH).
2. After the protein preparation, energy minimization was carried out using the YASARA energy minimization server.
3. The **AutoDock Vina** integrated into the **PyRx** computational platform <https://pyrx.sourceforge.io/> was used for the docking study, the protein file converted into macromolecule (pdbqt) and ligand files were converted Auto dock ligand pdbqt for the docking step.
4. The binding pocket dimensions used as attached in the following table, the docking study validation was reliable by re-docking of the native ligand AZD 5438 alongside the tested derivatives, yielding a root-mean-square deviation (RMSD) value of 0.3 Å.

| Grid  Box  dimension | CDK-2  (PDB code: 6GUH). |
| --- | --- |
| X | 24.06 A^º^ |
| Y | 21.37 A^º^ |
| Z | 25 A^º^ |
| Centre x | 3.35 |
| Centre y | -2.68 |
| Centre z | -29.57 |

1. Two-dimensional (2D) and three-dimensional (3D) binding interactions were generated by *Biovia Discovery Studio 2021* <https://discover.3ds.com>.

**References:**

[1] http:// dtp. nci. nih. gov.

[2] Monks A, Scudiero D, Skehan P, Shoemaker R, Paull K, Vistica D, Hose C, Langley J, Cronise P, Vaigro‑Wolff A. Feasibility of a high‑flux anticancer drug screen using a diverse panel of cultured human tumor cell lines. J Natl Cancer Inst. 1991; 83:757–66. https:// doi. org/ 10. 1093/ jnci/ 83. 11. 757.

[3] Boyd MR. In: Teicher BA, editor. Cancer Drug Discovery and Development, 2. USA: Humana Press; 1997. p. 23–43.

[4] Shoemaker RH. The NCI60 human tumor cell line anticancer drug screen. Nat Rev Cancer. 2006; 6:813–23. https:// doi. org/ 10. 1038/ nrc1951.
